# Supplementary material for: Threshold-Based Overlap of Breast Cancer High-Risk Classification Using Family History, Polygenic Risk Scores, and Traditional Risk Models in 180,398 Women
Source: Cancers (Basel). 2025 Nov 3;17(21):3561. doi: 10.3390/cancers17213561 (PMC12610763; doi:10.3390/cancers17213561)
Supplement: Supplementary file 1 [file cancers-17-03561-s001.zip › cancers-3892318-supplementary.pdf]

SUPPLEMENTARY MATERIAL

Supplementary Table S1. List of studies included. ... 3

Supplementary Table S2. Allele frequencies of the 313 variants in the breast cancer risk polygenic risk score. Chr: Chromosome. Position in b37. ... 8

Supplementary Table S3. Mean and standard deviation of polygenic risk score in non-breast cancer controls from Europeans and Asians, before exclusions of studies with high missing rates for Gail model risk factors. ... 37

Supplementary Table S4. Demographic and risk factors differences between included and excluded individuals. ... 38

Supplementary Table S5. Demographic and risk factors differences between included (Yes) and excluded (No) individuals of European-ancestry aged 30 to 49 years. ... 39

Supplementary Table S6. Demographic and risk factors differences between included (Yes) and excluded (No) individuals of European-ancestry aged 50 to 80 years. ... 40

Supplementary Table S7. Demographic and risk factors differences between included (Yes) and excluded (No) individuals of Asian-ancestry aged 30 to 49 years. No DCIS were excluded. ... 41

Supplementary Table S8. Demographic and risk factors differences between included (Yes) and excluded (No) individuals of Asian-ancestry aged 50 to 80 years. ... 42

Supplementary Table S9. Sensitivity analysis of the discriminatory ability of the Gail model, assuming that missing values correspond to the highest risk category rather than the reference category. Five-year absolute risk was estimated using the R package BCRA and applied to predict the risk of invasive breast cancer. AUC: Area under the receiver operator curve, CI: Confidence interval, AgeMen: Age at menarche, Age1st: Age at first live birth, N_Rels: Number of first-degree relatives with breast cancer, N _Biop: Number of prior biopsy. *Original model: the model using the conventional approach of ssigning missing values to the lowest risk group ... 43

Supplementary Table S10. Sensitivity analysis of the discriminatory ability of the Gail model, assuming that missing values correspond to the highest risk category rather than the reference category. Five-year absolute risk was estimated using the R package BCRA and applied to predict the risk of DCIS. AUC: Area under the receiver operator curve, CI: Confidence interval, AgeMen: Age at menarche, Age1st: Age at first live birth, N_Rels: Number of first-degree relatives with breast cancer, N_Biop: Number of prior biopsy. *Original model: the model using the conventional approach of assigning missing values to the lowest risk group ... 44

Supplementary Table S11. Association between five-year absolute breast cancer risk (modeled as a continuous variable, estimated using the polygenic risk score (PRS) versus the Gail model) and the development of invasive breast cancer or ductal carcinoma in situ (DCIS), stratified by ancestry and age groups, using only population-based controls. OR: odds ratios; CI: confidence intervals; AUC: area under the curve. ORs and AUCs are estimated separately in age-stratified logistic regression models (<50y and ≥50y). P-values for interaction (risk score × Age) were obtained from separate logistic regression models that included the risk score, age (treated as a continuous variable), and their interaction term; the interaction test assesses statistical heterogeneity of the risk-score effect by age. ... 45

Supplementary Table S12. Distribution of Venn diagram segments across ancestry groups, age groups, and risk thresholds. ... 46

Supplementary Table S13. P-values for model calibration performance from the predtools package in R, assessed using: (A) mean calibration (predicted vs outcome event rate), (B) model-based receiver operating characteristic (mROC)/ROC equality, and (U) unified calibration testing both A and B. p-

values were computed based on the reference population (European, ≥50y), for all groups (European, <50y; Asian, ≥50y; Asian, <50y), and for both polygenic risk score (PRS) and the Gail model. A small p-value for each of the test indicates: (A) miscalibration of average predicted risk, (B) miscalibration in distribution of predicted risk, and (U) overall miscalibration. 54

Supplementary Table S14. Discriminatory ability of risk factor combinations in the Gail model, corresponding to **Figure 3**. Five-year absolute risk was calculated using the R package “BCRA” and used to predict the risk of invasive breast cancer case-control. AUC: Area under the receiver operator curve, CI: Confidence interval, AgeMen: Age at menarche, Age1st: Age at first live birth, N_Rels: Number of first-degree relatives with breast cancer, N_Biop: Number of prior biopsies..................... 55

Supplementary Figure S1. Flowchart of individuals selected. 57

Supplementary Figure S2. Distribution of polygenic risk scores (scoresum) and five-year absolute risk by country, for individuals of European-ancestry. 58

Supplementary Figure S3. Distribution of polygenic risk scores (scoresum) and 5-year absolute risk by country, for individuals of Asian-ancestry... 59

Supplementary Figure S4. Sensitivity analysis of the discriminatory ability of the Gail model, assuming that missing values correspond to the highest risk category rather than the reference category. Five-year absolute risk was estimated using the R package BCRA and applied to predict the risk of invasive breast cancer in the case-control dataset. Dots represent models in which missing values for each risk factor were assigned to the highest risk group, while “X” denotes the model using the conventional approach of assigning missing values to the lowest risk group. European: women of European ancestry; Asian: women of Asian ancestry. 60

Supplementary Figure S5. Sensitivity analysis of the discriminatory ability of the Gail model, assuming that missing values correspond to the highest risk category rather than the reference category. Five-year absolute risk was estimated using the R package BCRA and applied to predict the risk of DCIS in the case-control dataset. Dots represent models in which missing values for each risk factor were assigned to the highest risk group, while “X” denotes the model that using the conventional approach of assigning missing values to the lowest risk group. European: women of European ancestry; Asian: women of Asian ancestry 61

Supplementary Figure S6. Proportion of individuals identified as high-risk by the polygenic risk score (PRS), the Gail model, and family history (FH) at varying absolute risk thresholds for PRS and Gail. Each line represents one identification method (black: PRS only; grey: Gail only; orange: FH only). . 62

Supplementary Figure S7. Proportion of individuals identified as high-risk by multiple factors across varying absolute-risk thresholds. PRS: polygenic risk score; Gail: the Gail model; FH: family history. These values match the overlapping regions from the corresponding Venn diagrams for each threshold. 63

Supplementary Figure S8. Proportion of individuals identified as at high risk by the breast cancer polygenic risk score (PRS) and the Gail model (GAIL), by country and age. The proportion of individuals identified as high risk by both criteria is indicated in grey. PRS: 5-year absolute risk using polygenic risk score ≥1·66%. GAIL: 5-year absolute risk using the Gail model ≥1.66%. Numbers adjacent to the bars represent the number of high-risk individuals identified by respective risk tools. European: women of European-ancestry; Asian: women of Asian-ancestry. 64

BCAC Funding and Acknowledgments 65

Supplementary Table S1. List of studies included.

Study	Acronym	Country	Type of control	Identifier for within study strata	Identifier for within study strata	Specify (other)	n
The Two Sister Study	2SISTER	USA	Not applicable (for cases)	Not applicable	Other	Population based familial	1067
Australian Breast Cancer Family Study	ABCFS	Australia	Population-based	Not applicable	Control		2065
Amsterdam Breast Cancer Study	ABCS	Netherlands	Not applicable (for cases)	ABCS-2	Sporadic (population or hospital based)		2477
Australian Breast Cancer Tissue Bank	ABCTB	Australia	Not applicable (for cases)	Not applicable	Sporadic (population or hospital based)		856
Asia Cancer Program	ACP	Thailand	Hospital-based	Not applicable	Control		1806
	AHS	USA	Nested case-control	Don't Know	Control		1624
Bavarian Breast Cancer Cases and Controls,	BBCC	Germany	Hospital-based	BBCC2	Control		1495
British Breast Cancer Study,	BBCS	UK	Not applicable (for cases)	NCRN	Other		106
Breast Cancer Employment and Environment Study	BCEES	Australia	Population-based	Not applicable	Control		1615
Philadelphia Breast Cancer Family Registry,	BCFR-PA	USA	Don't Know	Don't Know	Familial (clinical genetic centre based)		66
Breast Cancer in Northern Israel Study,	BCINIS	Israel	Population-based	Not applicable	Control		1920
Breast Oncology Galicia Network,	BREOGAN	Spain	Population-based	Don't Know	Control		2494
Canadian Breast Cancer Study-	CBCS	Canada	Population-based	Don't Know	Control		1995
Crete Cancer Genetics Program	CCGP	Greece	Not applicable (for cases)	Not applicable	Sporadic (population or hospital based)		658
CECILE Breast Cancer Study	CECILE	France	Population-based	Not applicable	Control		2010
Copenhagen General Population Study	CGPS	Denmark	Not applicable (for cases)	Copenhagen hospital	Sporadic (population or hospital based)		79
Cancer Prevention Study-II Nutrition Cohort	CPSII	USA	Not applicable (for cases)	NUTRITION COHORT	Sporadic (population or hospital based)		6240
California Teachers Study	CTS	USA	Don't Know	Not applicable	Control		1803
DietCompLyf Breast Cancer Survival Study	DIETCOMPLYF	UK	Not applicable (for cases)	Not applicable	Sporadic (population or hospital based)		711
European Prospective Investigation Into Cancer and Nutrition	EPIC	France	Nested case-control	EPIC France	Control		800

Study	Acronym	Country	Type of control	Identifier for within study strata	Identifier for within study strata	Specify (other)	n
European Prospective Investigation Into Cancer and Nutrition	EPIC	Germany	Nested case-control	EPIC Germany	Control		1307
European Prospective Investigation Into Cancer and Nutrition	EPIC	Italy	Nested case-control	EPIC Italy	Control		1601
European Prospective Investigation Into Cancer and Nutrition	EPIC	Netherlands	Not applicable (for cases)	EPIC The Netherlands	Sporadic (population or hospital based)		1364
European Prospective Investigation Into Cancer and Nutrition	EPIC	Spain	Nested case-control	EPIC Spain	Control		643
European Prospective Investigation Into Cancer and Nutrition	EPIC	UK	Nested case-control	EPIC United Kingdom	Control		1346
ESTHER Breast Cancer Study	ESTHER	Germany	Population-based	e1	Control		984
	FHRISK	UK	Not applicable (for cases)	Not applicable	Familial (clinical genetic centre based)		748
Gene Environment Interaction and Breast Cancer in Germany,	GENICA	Germany	Not applicable (for cases)	Not applicable	Sporadic (population or hospital based)		1626
Genetic Epidemiology Study of Breast Cancer by Age	GESBC	Germany	Not applicable (for cases)	Not applicable	Sporadic (population or hospital based)		504
Hannover Breast Cancer Study	HABCS	Germany	Not applicable (for cases)	HaBCS1	Sporadic (population or hospital based)		896
Hospital-based Epidemiologic Research Program at Aichi Cancer Center	HERPACC	Japan	Hospital-based	Not applicable	Control		2543
Hong Kong Breast Cancer Study,	HKBCS	Hong Kong	Population-based	Not applicable	Control		953
Hannover-Minsk Breast Cancer Study	HMBCS	Belarus	Not applicable (for cases)	Not applicable	Sporadic (population or hospital based)		689
	ICICLE	UK	Not applicable (for cases)	Not applicable	Sporadic (population or hospital based)		2823
Karolinska Breast Cancer Study,	KARBAC	Sweden	Not applicable (for cases)	Not applicable	Sporadic (population or hospital based)		760
Karolinska Mammography Project for Risk Prediction of Breast Cancer	KARMA	Sweden	Population-based	KARMA	Control		10100
Kuopio Breast Cancer Project,	KBCP	Finland	Not applicable (for cases)	Not applicable	Sporadic (population or hospital based)		945

Study	Acronym	Country	Type of control	Identifier for within study strata	Identifier for within study strata	Specify (other)	n
Kathleen Cuningham Foundation Consortium for Research into Familial Aspects of Breast Cancer (kConFab)/Australian Ovarian Cancer Study	KCONFAB/AOCS	Australia	Not applicable (for cases)	kConFab	Familial (clinical genetic centre based)		1401
Korean Hereditary Breast Cancer Study	KOHBRA	Korea	National 43 centers (for cases)	KOHBRA	Familial (National 43 genetic centre based) or Controls (population or center based control)	Controls includes carriers' family members or normal control from general population	1936
Leuven Multidisciplinary Breast Centre,	LMBC	Belgium	Not applicable (for cases)	retrospective	Sporadic (population or hospital based)		3364
Mammary Carcinoma Risk Factor Investigation	MARIE	Germany	Population-based	Not applicable	Control		3867
Milan Breast Cancer Study Group,	MBCSG	Italy	Not applicable (for cases)	IEO	Familial (clinical genetic centre based)		757
Mayo Clinic Breast Cancer Study	MCBCS	USA	Hospital-based	Not applicable	Control		4268
Melbourne Collaborative Cohort Study	MCCS	Australia	Nested case-control	Not applicable	Control		2163
Multi-ethnic Cohort	MEC	USA	Not applicable (for cases)	Don't Know	Sporadic (population or hospital based)		1576
Melanoma Inquiry of Southern Sweden	MISS	Sweden	Not applicable (for cases)	Don't Know	Sporadic (population or hospital based)		2136
Mayo Mammography Health Study	MMHS	USA	Nested case-control	Not applicable	Control		1954
	MSKCC	USA	Not applicable (for cases)	TNBCC	Familial (clinical genetic centre based)		135
Malaysian Breast Cancer Genetic Study	MYBRCA	Malaysia	Not applicable (for cases)	Not applicable	Sporadic (population or hospital based)		3152
Nashville Breast Health Study	NBHS	USA	Not applicable (for cases)	Not applicable	Sporadic (population or hospital based)		1387
Northern California Breast Cancer Family Registry	NC-BCFR	USA	Not applicable (for cases)	Not applicable	Other	Population based familial	1392
	NCBCS	USA	Not applicable (for cases)	Not applicable	Sporadic (population or hospital based)		3369
Nagano Breast Cancer Study	NGOBCS	Japan	Hospital-based	Not applicable	Control		731
Nurses Health Study	NHS	USA	Nested case-control	Not applicable	Control		3075
Nurses Health Study	NHS2	USA	Nested case-control	Not applicable	Control		3423

Study	Acronym	Country	Type of control	Identifier for within study strata	Identifier for within study strata	Specify (other)	n
Ontario Familial Breast Cancer Registry	OFBCR	Canada	Not applicable (for cases)	Not applicable	Control		3156
Leiden University Medical Centre Breast Cancer Study,	ORIGO	Netherlands	Not applicable (for cases)	Not applicable	Sporadic (population or hospital based)		1351
NCI Polish Breast Cancer Study	PBCS	Poland	Not applicable (for cases)	Not applicable	Sporadic (population or hospital based)		3953
	PKARMA	Sweden	Not applicable (for cases)	PKARMA	Sporadic (population or hospital based)		10923
The Prostate, Lung, Colorectal and Ovarian (PLCO) Cancer Screening Trial	PLCO	USA	Not applicable (for cases)	Don't Know	Sporadic (population or hospital based)		4808
	PREFACE	Germany	Not applicable (for cases)	Not applicable	Sporadic (population or hospital based)		2842
	PROCAS	UK	Population-based	Not applicable	Control		2123
Rotterdam Breast Cancer Study	RBCS	Netherlands	Not applicable (for cases)	Not applicable	Familial (clinical genetic centre based)		1038
	SASBAC	Sweden	Population-based	Not applicable	Control		2502
Shanghai Breast Cancer Genetic Study	SBCGS	China	Population-based	Don't Know	Control		3511
	SBCS	UK	Not applicable (for cases)	Not applicable	Sporadic (population or hospital based)		1743
Study of Epidemiology and Risk factors in Cancer Heredity	SEARCH	UK	Not applicable (for cases)	SEARCH	Sporadic (population or hospital based)		21332
Seoul Breast Cancer Study,	SEBCS	Korea	Not applicable (for cases)	Not applicable	Sporadic (population or hospital based)		135
Singapore Breast Cancer Cohort	SGBCC	Singapore	Not applicable (for cases)	SBCCP	Sporadic (population or hospital based)		894
The Sister Study	SISTER	USA	Not applicable (for cases)	Not applicable	Familial (clinical genetic centre based)		3548
Städtisches Klinikum Karlsruhe Deutsches Krebsforschungszentrum Study	SKKDKFZS	Germany	Not applicable (for cases)	Not applicable	Sporadic (population or hospital based)		1174
Swedish Mammography Cohort	SMC	Sweden	Nested case-control	Not applicable	Control		2086
	SUCCESSC	Germany	Not applicable (for cases)	NA	Familial (clinical genetic centre based)		2816
Taiwanese Breast Cancer Study,	TWBCS	Taiwan	Not applicable (for cases)	Not applicable	Sporadic (population or hospital based)		1889
	UBCS	USA	Not applicable (for cases)	Other	Sporadic (population or hospital based)		645
UCI Breast Cancer Study,	UCIBCS	USA	Not applicable (for cases)	Not applicable	Sporadic (population or hospital based)		726

Study	Acronym	Country	Type of control	Identifier for within study strata	Identifier for within study strata	Specify (other)	n
UK Breakthrough Generations Study	UKBGS	UK	Nested case-control	Not applicable	Control		2057
	USRT	USA	Not applicable (for cases)	Don't Know	Sporadic (population or hospital based)		3341

Supplementary Table S2. Allele frequencies of the 313 variants in the breast cancer risk polygenic risk score. Chr: Chromosome. Position in b37.

					Allele frequency of A0											
					Asian-ancestry											
					Control				Invasive				In-situ			
					Age <50		Age≥50		Age <50		Age≥50		Age <50		Age≥50	
Chr	Position	A0	A1	Beta	ICOGS	OncoArray	ICOGS	OncoArray	ICOGS	OncoArray	ICOGS	OncoArray	ICOGS	OncoArray	ICOGS	OncoArray
1	7917076	G	A	2.612	0.210	0.187	0.203	0.199	0.202	0.192	0.191	0.197	0.203	0.195	0.225	0.184
1	10566215	A	G	2.568	0.320	0.336	0.303	0.321	0.311	0.318	0.311	0.305	0.316	0.343	0.275	0.285
1	18807339	T	C	2.573	0.790	0.847	0.796	0.852	0.796	0.835	0.795	0.840	0.787	0.851	0.803	0.833
1	41380440	C	T	2.839	0.643	0.661	0.651	0.667	0.645	0.661	0.651	0.655	0.630	0.676	0.643	0.638
1	41389220	T	C	3.214	0.010	0.005	0.006	0.003	0.009	0.004	0.006	0.004	0.003	0.004	0.003	0.003
1	46670206	TC	T	2.845	0.013	0.009	0.011	0.009	0.021	0.009	0.013	0.011	0.015	0.011	0.017	0.014
1	51467096	CT	C	2.824	0.314	0.323	0.308	0.308	0.314	0.319	0.318	0.313	0.333	0.323	0.307	0.323
1	88156923	G	A	2.859	0.009	0.004	0.013	0.002	0.011	0.003	0.008	0.002	0.005	0.002	0.000	0.005
1	88428199	C	A	2.617	0.126	0.110	0.144	0.116	0.127	0.116	0.123	0.113	0.098	0.092	0.141	0.101
1	100880328	A	T	2.824	0.532	0.549	0.522	0.554	0.527	0.540	0.526	0.540	0.506	0.544	0.521	0.519
1	110198129	CAAA	C	2.849	0.915	0.930	0.917	0.940	0.926	0.929	0.916	0.932	0.932	0.948	0.936	0.942
1	114445880	G	A	2.898	0.538	0.595	0.523	0.605	0.561	0.597	0.560	0.596	0.581	0.606	0.610	0.622
1	118141492	A	C	2.847	0.414	0.413	0.394	0.424	0.415	0.412	0.427	0.439	0.391	0.429	0.452	0.433
1	120257110	T	C	2.827	0.906	0.922	0.905	0.930	0.905	0.920	0.927	0.918	0.902	0.926	0.922	0.932
1	121280613	A	G	2.981	0.041	0.026	0.038	0.032	0.045	0.036	0.038	0.037	0.029	0.041	0.026	0.026
1	121287994	A	G	2.547	0.176	0.154	0.171	0.148	0.170	0.143	0.154	0.147	0.141	0.148	0.113	0.135

1	145604302	C	CT	2.614	0.146	0.157	0.136	0.160	0.171	0.161	0.164	0.166	0.129	0.148	0.124	0.131
1	149906413	T	C	2.876	0.278	0.301	0.264	0.314	0.320	0.311	0.320	0.327	0.317	0.321	0.319	0.355
1	155556971	G	A	2.861	0.645	0.690	0.643	0.692	0.643	0.695	0.653	0.681	0.695	0.695	0.700	0.696
1	168171052	CA	C	2.545	0.008	0.002	0.008	0.001	0.007	0.001	0.006	0.001	0.005	0.000	0.004	0.000
1	172328767	T	TA	2.605	0.130	0.128	0.133	0.140	0.141	0.129	0.145	0.137	0.119	0.139	0.112	0.138
1	201437832	C	T	2.992	0.006	0.000	0.006	0.000	0.005	0.000	0.004	0.000	0.002	0.002	0.003	0.003
1	202184600	C	T	2.701	0.228	0.240	0.228	0.213	0.246	0.222	0.233	0.230	0.187	0.223	0.194	0.229
1	203770448	T	A	2.861	0.322	0.316	0.340	0.334	0.345	0.332	0.344	0.349	0.315	0.342	0.357	0.379
1	204502514	T	TTCTGAA ACAGGG	2.634	0.985	0.996	0.991	0.997	0.987	0.996	0.990	0.994	0.995	0.994	0.990	0.992
1	208076291	G	A	2.622	0.020	0.027	0.017	0.020	0.018	0.023	0.017	0.018	0.013	0.028	0.019	0.023
1	217053815	T	G	2.837	0.186	0.161	0.185	0.178	0.178	0.162	0.187	0.169	0.160	0.158	0.167	0.161
1	217220574	G	A	2.604	0.060	0.043	0.063	0.050	0.057	0.049	0.057	0.050	0.048	0.054	0.051	0.041
1	220671050	C	T	2.837	0.329	0.346	0.346	0.357	0.326	0.346	0.340	0.343	0.370	0.380	0.356	0.364
1	242034263	A	G	3.169	0.001	0.000	0.002	0.000	0.002	0.000	0.002	0.000	1.43097 e-05	0.000	0.000	0.000
2	10138983	T	C	2.893	0.006	0.002	0.011	0.002	0.008	0.003	0.006	0.002	0.004	0.004	0.003	7.67626e-05
2	19315675	T	A	2.631	0.567	0.589	0.558	0.593	0.571	0.570	0.574	0.581	0.533	0.550	0.553	0.570
2	25129473	A	G	2.607	0.417	0.424	0.409	0.422	0.402	0.406	0.407	0.412	0.369	0.428	0.431	0.411
2	29179452	G	C	2.700	0.740	0.783	0.724	0.780	0.759	0.787	0.757	0.779	0.813	0.788	0.805	0.804
2	29615233	T	C	2.607	0.039	0.020	0.045	0.023	0.038	0.021	0.037	0.024	0.028	0.024	0.020	0.016
2	39699510	C	CT	2.613	0.786	0.821	0.784	0.828	0.787	0.816	0.790	0.823	0.787	0.827	0.811	0.821
2	70172587	G	A	2.611	0.307	0.279	0.299	0.296	0.292	0.278	0.285	0.284	0.313	0.293	0.293	0.267

2	88358825	G	C	2.853	0.245	0.275	0.259	0.281	0.261	0.286	0.252	0.287	0.228	0.265	0.291	0.265
2	121058254	A	G	2.630	0.446	0.424	0.436	0.389	0.439	0.395	0.413	0.400	0.371	0.409	0.390	0.406
2	121089731	T	C	2.607	0.079	0.084	0.091	0.083	0.082	0.093	0.075	0.077	0.084	0.114	0.061	0.078
2	121159205	G	A	2.604	0.154	0.129	0.152	0.147	0.164	0.129	0.154	0.141	0.118	0.136	0.156	0.142
2	121246568	T	C	3.017	0.780	0.774	0.800	0.773	0.761	0.790	0.781	0.793	0.858	0.792	0.803	0.753
2	172974566	C	G	2.596	0.205	0.207	0.219	0.211	0.212	0.201	0.203	0.208	0.201	0.180	0.167	0.210
2	174212910	A	G	2.890	0.978	0.991	0.977	0.991	0.987	0.992	0.987	0.991	1.000	0.998	0.994	0.997
2	192381934	C	T	2.807	0.940	0.934	0.938	0.931	0.940	0.932	0.941	0.935	0.928	0.926	0.945	0.911
2	202204741	T	C	2.591	0.709	0.716	0.716	0.711	0.715	0.703	0.707	0.689	0.658	0.708	0.708	0.677
2	217920769	G	T	2.403	0.866	0.887	0.853	0.888	0.858	0.880	0.865	0.879	0.880	0.884	0.870	0.886
2	217955896	GA	G	2.265	0.001	0.000	0.002	0.000	0.001	0.000	0.001	0.000	4.80396e-05	0.002	1.94203e-05	0.000
2	218292158	C	G	2.527	0.349	0.340	0.371	0.374	0.340	0.334	0.332	0.343	0.344	0.362	0.320	0.387
2	218714845	G	A	2.606	0.255	0.254	0.246	0.251	0.256	0.245	0.265	0.253	0.269	0.235	0.255	0.295
2	241388857	C	A	2.421	0.999	1.000	0.999	1.000	0.999	1.000	0.999	1.000	0.999	0.998	1.000	1.000
3	4742251	A	G	2.897	0.090	0.081	0.081	0.090	0.087	0.091	0.099	0.090	0.096	0.088	0.061	0.090
3	27353716	C	A	2.938	0.291	0.260	0.275	0.252	0.301	0.269	0.286	0.263	0.297	0.266	0.264	0.256
3	27388664	C	G	2.862	0.155	0.152	0.146	0.144	0.164	0.156	0.165	0.153	0.153	0.166	0.136	0.159
3	29294845	C	T	2.410	0.000	0.000	0.001	0.000	0.000	0.000	0.000	0.000	0.003	4.85029e-05	2.37799e-05	0.000
3	30684907	C	T	2.889	0.635	0.673	0.627	0.680	0.659	0.674	0.674	0.683	0.653	0.671	0.684	0.674
3	46888198	T	C	2.516	0.163	0.146	0.161	0.148	0.160	0.142	0.147	0.137	0.146	0.123	0.132	0.110
3	49709912	C	CT	2.622	0.068	0.058	0.066	0.051	0.058	0.060	0.054	0.052	0.065	0.065	0.066	0.054

3	55970777	A	AT	2.429	0.001	0.000	0.001	0.000	0.001	2.70471e-05	0.001	0.000	0.000	9.37531e-06	8.42866e-05	4.94739e-05
3	59373745	C	T	2.615	0.641	0.659	0.632	0.677	0.657	0.667	0.646	0.674	0.625	0.637	0.658	0.652
3	63887449	T	TTG	2.906	0.117	0.139	0.122	0.143	0.144	0.143	0.130	0.135	0.182	0.149	0.132	0.140
3	71620370	T	G	2.620	0.061	0.035	0.068	0.038	0.062	0.034	0.059	0.035	0.047	0.030	0.039	0.043
3	87037543	A	G	2.535	0.010	0.005	0.010	0.004	0.009	0.005	0.006	0.005	0.002	0.005	0.002	0.006
3	99403877	G	A	2.620	0.455	0.490	0.469	0.495	0.469	0.479	0.487	0.476	0.484	0.488	0.480	0.462
3	141112859	CTT	C	2.877	0.076	0.059	0.076	0.057	0.071	0.059	0.077	0.062	0.041	0.056	0.056	0.039
3	172285237	G	A	2.838	0.315	0.325	0.309	0.345	0.331	0.332	0.334	0.338	0.332	0.349	0.314	0.305
3	189774456	C	T	2.594	0.124	0.090	0.117	0.087	0.113	0.086	0.116	0.087	0.103	0.080	0.107	0.085
4	38784633	G	T	2.858	0.471	0.471	0.505	0.470	0.460	0.479	0.474	0.467	0.507	0.480	0.478	0.454
4	84370124	TAA	TA	2.598	0.679	0.688	0.674	0.680	0.687	0.676	0.680	0.680	0.689	0.698	0.694	0.691
4	89240476	G	A	2.817	0.030	0.013	0.038	0.013	0.028	0.014	0.025	0.016	0.014	0.007	0.017	0.012
4	92594859	TTCTTTC	T	2.612	0.591	0.603	0.573	0.599	0.570	0.589	0.560	0.602	0.582	0.580	0.574	0.617
4	106069013	G	T	2.853	0.603	0.618	0.580	0.613	0.583	0.615	0.595	0.618	0.660	0.646	0.635	0.616
4	126752992	A	AAT	2.620	0.372	0.375	0.385	0.380	0.366	0.383	0.374	0.373	0.385	0.371	0.361	0.360
4	143467195	C	T	2.572	0.043	0.029	0.038	0.024	0.039	0.029	0.040	0.037	0.040	0.030	0.031	0.038
4	151218296	CATATTT	C	2.828	0.267	0.258	0.275	0.252	0.252	0.259	0.267	0.248	0.305	0.246	0.287	0.222
4	175842495	G	A	2.495	0.237	0.248	0.232	0.260	0.245	0.247	0.255	0.247	0.239	0.251	0.223	0.235
4	175847436	C	A	2.816	0.050	0.023	0.042	0.021	0.036	0.020	0.037	0.022	0.018	0.022	0.022	0.022
4	187503758	A	T	2.819	0.380	0.414	0.398	0.418	0.401	0.398	0.405	0.416	0.404	0.382	0.382	0.400
5	345109	T	C	2.967	0.008	0.003	0.009	0.003	0.008	0.004	0.004	0.005	9.63713e-06	0.009	8.5872e-06	0.003
5	1279790	C	T	2.897	0.208	0.185	0.193	0.177	0.188	0.209	0.199	0.192	0.199	0.176	0.216	0.206

5	1296255	A	AG	2.577	0.359	0.365	0.344	0.357	0.368	0.344	0.368	0.357	0.309	0.353	0.323	0.334
5	1353077	T	C	3.215	0.000	0.000	0.000	0.000	0.001	0.000	0.000	0.000	0.000	0.000	4.54461 e-05	0.000
5	2777029	G	A	2.829	0.743	0.755	0.730	0.769	0.746	0.755	0.760	0.779	0.728	0.742	0.726	0.758
5	16231194	G	C	2.607	0.172	0.160	0.191	0.158	0.182	0.156	0.163	0.164	0.151	0.163	0.128	0.140
5	32579616	TCA	T	2.821	0.136	0.112	0.144	0.116	0.123	0.113	0.123	0.122	0.120	0.124	0.170	0.110
5	44508264	G	GT	2.433	0.013	0.005	0.010	0.004	0.011	0.004	0.008	0.005	0.004	0.004	0.004	0.003
5	44619502	A	G	2.449	0.003	0.001	0.004	0.001	0.002	0.001	0.002	0.001	0.002	0.002	0.002	0.000
5	44649944	C	T	2.859	0.586	0.574	0.580	0.557	0.606	0.593	0.592	0.588	0.634	0.610	0.608	0.638
5	44706498	A	G	2.860	0.503	0.502	0.490	0.490	0.530	0.521	0.515	0.517	0.553	0.549	0.541	0.561
5	44853593	G	C	2.630	0.065	0.066	0.076	0.060	0.052	0.064	0.055	0.047	0.074	0.058	0.043	0.046
5	52679539	C	CA	2.883	0.193	0.203	0.197	0.218	0.192	0.200	0.208	0.197	0.195	0.202	0.193	0.200
5	55662540	C	CT	2.599	0.354	0.387	0.365	0.395	0.362	0.389	0.369	0.380	0.395	0.391	0.380	0.383
5	55965167	C	T	2.830	0.622	0.569	0.626	0.566	0.625	0.589	0.621	0.586	0.587	0.560	0.602	0.599
5	56023083	T	G	3.147	0.374	0.357	0.350	0.367	0.379	0.374	0.365	0.361	0.371	0.373	0.387	0.374
5	56042972	C	T	2.975	0.103	0.109	0.096	0.111	0.113	0.131	0.120	0.116	0.151	0.141	0.126	0.142
5	56045081	T	C	2.573	0.049	0.041	0.057	0.041	0.041	0.041	0.046	0.041	0.036	0.028	0.048	0.051
5	58241712	C	T	2.605	0.660	0.671	0.652	0.679	0.669	0.678	0.676	0.682	0.658	0.679	0.655	0.633
5	71965007	G	A	2.611	0.141	0.139	0.152	0.126	0.154	0.129	0.137	0.138	0.098	0.120	0.114	0.132
5	73234583	T	C	2.623	0.386	0.430	0.410	0.441	0.376	0.444	0.397	0.436	0.433	0.445	0.429	0.435
5	77155397	GT	G	2.612	0.190	0.187	0.178	0.200	0.185	0.174	0.193	0.186	0.182	0.177	0.195	0.191
5	79180995	G	GA	2.810	0.365	0.367	0.361	0.371	0.375	0.379	0.377	0.382	0.363	0.402	0.396	0.411
5	81512947	TA	T	2.565	0.012	0.001	0.010	0.000	0.005	0.002	0.007	0.002	0.000	0.000	3.03855 e-05	0.002

5	90789470	G	A	2.573	0.470	0.476	0.494	0.494	0.464	0.476	0.457	0.457	0.481	0.494	0.453	0.447
5	104300273	G	T	2.592	0.083	0.069	0.081	0.066	0.066	0.069	0.072	0.065	0.083	0.090	0.075	0.058
5	122478676	C	A	2.617	0.603	0.603	0.615	0.610	0.604	0.621	0.598	0.610	0.632	0.631	0.596	0.636
5	122705244	C	T	3.001	0.097	0.100	0.102	0.096	0.108	0.107	0.104	0.100	0.115	0.085	0.107	0.097
5	131640536	A	G	2.829	0.285	0.270	0.283	0.267	0.261	0.257	0.256	0.276	0.266	0.288	0.239	0.272
5	132407058	C	T	2.617	0.132	0.116	0.133	0.114	0.130	0.112	0.131	0.100	0.125	0.100	0.116	0.118
5	158244083	C	T	2.546	0.387	0.374	0.383	0.355	0.387	0.339	0.355	0.352	0.323	0.357	0.316	0.341
5	169591460	T	C	2.835	0.487	0.458	0.476	0.459	0.474	0.461	0.484	0.486	0.497	0.489	0.501	0.507
5	173358154	G	A	2.821	0.326	0.298	0.311	0.278	0.300	0.293	0.286	0.295	0.265	0.320	0.275	0.295
5	176134882	T	C	2.821	0.491	0.474	0.474	0.491	0.477	0.488	0.449	0.473	0.451	0.483	0.492	0.524
6	13713366	G	C	2.576	0.450	0.393	0.434	0.399	0.401	0.381	0.416	0.392	0.417	0.376	0.430	0.403
6	16399557	C	T	2.621	0.336	0.343	0.330	0.336	0.342	0.338	0.354	0.323	0.294	0.313	0.336	0.343
6	18783140	G	A	2.810	0.712	0.735	0.726	0.722	0.711	0.741	0.712	0.727	0.731	0.707	0.725	0.745
6	20537845	CA	C	2.616	0.310	0.330	0.313	0.332	0.331	0.320	0.329	0.330	0.312	0.341	0.286	0.324
6	21923810	T	C	2.634	0.307	0.290	0.302	0.316	0.311	0.305	0.299	0.300	0.299	0.312	0.296	0.335
6	27425644	G	C	2.532	6.17532 e-06	0.000	5.01147 e-05	0.000	9.36314e -06	0.000	4.5166 e-06	0.000	4.81856 e-06	0.000	0.000	0.000
6	43227141	G	A	2.555	0.009	0.007	0.015	0.006	0.010	0.006	0.011	0.008	0.011	0.005	0.004	0.003
6	82263549	AAT	A	2.854	0.412	0.405	0.423	0.395	0.388	0.396	0.396	0.388	0.462	0.390	0.445	0.401
6	85912194	CAA	C	2.942	0.002	0.000	0.003	0.000	0.002	0.000	0.003	0.001	0.002	0.000	0.001	0.002
6	87803819	T	C	2.827	0.255	0.275	0.267	0.264	0.245	0.276	0.252	0.258	0.265	0.286	0.252	0.301
6	130341728	C	CT	2.853	0.893	0.918	0.887	0.927	0.897	0.919	0.907	0.922	0.932	0.929	0.927	0.919
6	149595505	T	C	2.595	0.416	0.414	0.425	0.428	0.380	0.415	0.396	0.415	0.422	0.421	0.448	0.421
6	151949806	A	C	2.924	0.322	0.341	0.311	0.337	0.390	0.378	0.386	0.379	0.352	0.375	0.431	0.398

6	151955914	A	G	3.177	0.276	0.316	0.267	0.314	0.345	0.349	0.343	0.352	0.330	0.351	0.390	0.369
6	152022664	CAAAAAAA	C	2.756	0.411	0.396	0.414	0.406	0.405	0.410	0.415	0.417	0.423	0.421	0.439	0.425
6	152023191	G	A	2.900	0.119	0.114	0.115	0.127	0.133	0.126	0.142	0.136	0.136	0.124	0.139	0.114
6	152055978	A	T	2.935	0.075	0.075	0.068	0.085	0.082	0.083	0.090	0.093	0.103	0.078	0.097	0.080
6	152432902	C	T	2.907	0.574	0.589	0.580	0.569	0.579	0.590	0.584	0.588	0.607	0.574	0.632	0.590
6	169006947	C	G	2.637	0.382	0.382	0.394	0.374	0.385	0.389	0.385	0.375	0.350	0.373	0.390	0.353
6	170332621	T	C	2.824	0.177	0.166	0.195	0.162	0.178	0.175	0.189	0.178	0.201	0.147	0.169	0.194
7	21940960	A	G	2.597	0.188	0.187	0.189	0.177	0.189	0.189	0.193	0.186	0.179	0.201	0.160	0.143
7	25569548	C	T	2.592	0.227	0.221	0.221	0.223	0.231	0.221	0.221	0.230	0.259	0.232	0.239	0.217
7	28869017	G	A	2.571	0.003	0.001	0.006	0.001	0.004	0.001	0.003	0.002	0.002	0.005	0.000	0.000
7	55192256	A	C	2.627	0.691	0.706	0.693	0.693	0.687	0.693	0.696	0.708	0.718	0.707	0.671	0.705
7	91459189	A	ATT	2.847	0.196	0.161	0.182	0.153	0.176	0.168	0.180	0.167	0.155	0.164	0.170	0.154
7	94113799	T	C	2.846	0.246	0.246	0.250	0.233	0.259	0.252	0.260	0.250	0.241	0.223	0.268	0.237
7	98005235	G	A	2.597	0.160	0.179	0.170	0.179	0.165	0.170	0.177	0.182	0.179	0.174	0.173	0.215
7	99948655	T	G	2.837	0.047	0.035	0.047	0.035	0.046	0.030	0.048	0.038	0.028	0.040	0.033	0.036
7	101552440	G	A	2.572	0.060	0.061	0.054	0.057	0.062	0.060	0.055	0.061	0.043	0.044	0.038	0.047
7	102481842	T	C	2.837	0.585	0.592	0.584	0.590	0.580	0.605	0.591	0.610	0.596	0.608	0.634	0.586
7	130656911	C	T	2.595	0.276	0.259	0.275	0.260	0.269	0.267	0.277	0.254	0.238	0.237	0.264	0.284
7	130674481	G	A	2.836	0.286	0.282	0.294	0.282	0.296	0.293	0.286	0.289	0.294	0.277	0.306	0.309
7	139943702	CT	C	2.886	0.546	0.539	0.544	0.524	0.546	0.537	0.558	0.556	0.503	0.547	0.574	0.577
7	144048902	G	T	2.573	0.045	0.037	0.043	0.034	0.043	0.033	0.042	0.040	0.018	0.024	0.034	0.048
8	170692	T	C	2.854	0.204	0.201	0.204	0.198	0.199	0.206	0.205	0.203	0.186	0.177	0.174	0.201
8	17787610	CT	C	2.620	0.333	0.342	0.345	0.347	0.346	0.338	0.344	0.337	0.328	0.342	0.343	0.354

8	23447496	A	G	2.617	0.844	0.864	0.851	0.866	0.838	0.865	0.845	0.861	0.866	0.850	0.881	0.845
8	23663653	C	A	2.812	0.314	0.301	0.307	0.310	0.342	0.310	0.326	0.320	0.297	0.296	0.348	0.367
8	29509616	A	C	2.564	0.715	0.699	0.710	0.710	0.715	0.695	0.695	0.692	0.656	0.674	0.669	0.659
8	36858483	A	G	2.526	0.327	0.332	0.337	0.350	0.299	0.325	0.335	0.320	0.306	0.348	0.294	0.346
8	76230943	A	G	2.940	0.971	0.975	0.975	0.972	0.978	0.982	0.979	0.981	0.995	0.977	0.985	0.985
8	76333056	C	T	3.063	0.004	0.000	0.002	0.001	0.001	0.001	0.002	0.001	0.002	0.000	0.000	0.000
8	76378165	G	T	2.616	0.257	0.251	0.246	0.237	0.252	0.231	0.246	0.242	0.268	0.263	0.217	0.234
8	102483100	T	C	2.890	0.009	0.004	0.007	0.003	0.008	0.003	0.004	0.005	0.004	0.003	0.000	0.005
8	106358620	A	T	2.530	0.118	0.105	0.126	0.105	0.122	0.099	0.119	0.107	0.113	0.077	0.111	0.115
8	117209548	A	G	2.609	0.448	0.452	0.420	0.455	0.457	0.435	0.438	0.457	0.410	0.425	0.469	0.449
8	120862186	A	G	2.869	0.031	0.026	0.031	0.025	0.033	0.025	0.036	0.030	0.029	0.023	0.023	0.032
8	124563705	T	C	2.854	0.167	0.187	0.174	0.183	0.177	0.186	0.181	0.193	0.191	0.175	0.189	0.192
8	124571581	G	A	2.814	0.370	0.364	0.364	0.381	0.362	0.364	0.374	0.373	0.366	0.355	0.330	0.378
8	124739913	T	G	2.851	0.264	0.242	0.255	0.237	0.265	0.257	0.250	0.255	0.233	0.259	0.275	0.286
8	128213561	C	CA	2.606	0.102	0.129	0.117	0.132	0.123	0.125	0.118	0.120	0.125	0.124	0.108	0.121
8	128370949	C	G	2.905	0.529	0.519	0.538	0.521	0.530	0.546	0.512	0.519	0.553	0.551	0.533	0.561
8	128372172	A	G	2.891	0.779	0.771	0.766	0.764	0.768	0.784	0.770	0.778	0.787	0.787	0.781	0.801
8	129199566	G	A	2.896	0.196	0.208	0.213	0.212	0.200	0.194	0.213	0.200	0.201	0.219	0.201	0.189
8	143669254	A	G	2.627	0.755	0.815	0.758	0.816	0.784	0.808	0.786	0.827	0.759	0.821	0.792	0.839
9	6880263	A	G	2.816	0.006	0.002	0.005	0.000	0.007	0.001	0.007	0.002	0.003	0.002	0.003	0.007
9	21964882	CAAAA	C	2.876	0.216	0.205	0.194	0.210	0.226	0.212	0.229	0.218	0.195	0.203	0.228	0.233
9	22041998	C	G	2.799	0.020	0.022	0.018	0.020	0.024	0.020	0.022	0.024	0.024	0.024	0.022	0.024
9	36928288	T	C	2.788	0.702	0.734	0.698	0.735	0.714	0.732	0.716	0.727	0.734	0.750	0.738	0.758

9	87782211	T	C	2.820	0.775	0.780	0.756	0.785	0.772	0.768	0.793	0.789	0.782	0.785	0.788	0.775
9	98362587	T	C	2.884	0.333	0.302	0.321	0.321	0.324	0.327	0.309	0.323	0.308	0.290	0.352	0.315
9	110303808	TAA	T	2.953	0.404	0.432	0.401	0.430	0.427	0.443	0.396	0.446	0.420	0.445	0.429	0.441
9	110837073	A	G	3.073	0.353	0.409	0.376	0.419	0.404	0.436	0.410	0.420	0.400	0.423	0.424	0.410
9	110837176	C	T	2.908	0.029	0.020	0.034	0.017	0.025	0.026	0.024	0.019	0.024	0.020	0.026	0.026
9	110849525	G	T	2.761	0.985	0.995	0.986	0.997	0.986	0.996	0.986	0.996	0.992	0.995	0.996	0.995
9	110885479	C	T	2.979	0.919	0.927	0.926	0.929	0.928	0.936	0.927	0.925	0.947	0.936	0.911	0.939
9	119313486	A	G	2.598	0.323	0.300	0.317	0.299	0.318	0.312	0.293	0.298	0.300	0.293	0.292	0.298
9	129424719	A	G	2.618	0.569	0.570	0.549	0.559	0.566	0.545	0.554	0.549	0.526	0.549	0.545	0.568
9	136146597	C	T	2.832	0.223	0.209	0.222	0.213	0.202	0.220	0.213	0.213	0.220	0.216	0.254	0.219
10	5794652	A	G	2.852	0.139	0.166	0.170	0.173	0.152	0.176	0.165	0.161	0.172	0.155	0.169	0.179
10	13892298	G	A	2.823	0.577	0.588	0.572	0.610	0.607	0.596	0.625	0.616	0.595	0.596	0.617	0.619
10	22032942	A	G	2.569	0.932	0.949	0.941	0.951	0.940	0.947	0.939	0.946	0.969	0.948	0.946	0.945
10	22477776	ACC	A	3.267	0.009	0.006	0.011	0.007	0.010	0.005	0.008	0.010	0.005	0.009	0.006	0.010
10	22861490	A	C	2.979	0.948	0.943	0.957	0.939	0.939	0.938	0.935	0.932	0.945	0.946	0.933	0.953
10	38523626	C	A	2.833	0.523	0.563	0.521	0.531	0.530	0.552	0.532	0.551	0.571	0.561	0.515	0.523
10	64299890	A	G	2.397	0.024	0.021	0.025	0.023	0.027	0.017	0.025	0.019	0.026	0.016	0.022	0.010
10	64819996	G	T	2.853	0.171	0.176	0.180	0.184	0.178	0.176	0.180	0.179	0.203	0.172	0.199	0.167
10	71335574	C	T	2.613	0.121	0.095	0.112	0.094	0.124	0.090	0.118	0.099	0.095	0.087	0.104	0.085
10	80851257	G	T	2.516	0.647	0.634	0.663	0.652	0.615	0.625	0.638	0.632	0.658	0.647	0.636	0.606
10	80886726	A	G	2.942	0.452	0.469	0.445	0.477	0.466	0.493	0.464	0.468	0.513	0.500	0.474	0.507
10	95292187	CAA	C	2.586	0.678	0.647	0.656	0.656	0.658	0.646	0.658	0.651	0.708	0.668	0.679	0.656
10	114777670	C	T	2.853	0.067	0.030	0.070	0.030	0.056	0.030	0.052	0.034	0.038	0.030	0.019	0.031

10	115128491	T	C	2.566	0.269	0.251	0.303	0.258	0.231	0.247	0.250	0.239	0.266	0.230	0.264	0.200
10	123095209	G	A	2.580	0.383	0.399	0.394	0.416	0.391	0.400	0.408	0.393	0.407	0.379	0.375	0.381
10	123340107	A	G	3.199	0.101	0.112	0.096	0.105	0.112	0.101	0.112	0.107	0.085	0.096	0.092	0.124
10	123340431	GC	G	2.195	0.541	0.569	0.568	0.563	0.508	0.503	0.519	0.510	0.540	0.525	0.531	0.536
10	123349324	A	T	2.161	0.012	0.008	0.011	0.005	0.012	0.006	0.010	0.005	0.008	0.004	0.009	0.010
11	433617	T	C	2.605	0.995	0.997	0.996	0.998	0.996	0.998	0.994	0.999	0.995	0.997	0.995	0.998
11	803017	A	G	2.848	0.256	0.249	0.277	0.275	0.254	0.262	0.283	0.272	0.316	0.239	0.331	0.260
11	1895708	C	A	2.526	0.689	0.704	0.705	0.711	0.665	0.702	0.689	0.697	0.765	0.710	0.690	0.689
11	18664241	T	G	2.850	0.507	0.487	0.510	0.490	0.529	0.481	0.513	0.498	0.502	0.488	0.532	0.504
11	42844441	C	T	2.630	0.222	0.219	0.200	0.201	0.235	0.214	0.224	0.215	0.189	0.249	0.178	0.224
11	44368892	G	A	2.824	0.298	0.310	0.319	0.315	0.324	0.336	0.324	0.333	0.347	0.335	0.310	0.365
11	46318032	C	G	2.529	0.006	0.001	0.006	0.000	0.006	0.001	0.005	0.000	0.005	7.84464e-05	0.009	0.000
11	65553492	C	A	2.839	0.101	0.098	0.108	0.090	0.107	0.102	0.103	0.108	0.093	0.131	0.096	0.128
11	65572431	G	A	2.627	0.237	0.224	0.234	0.196	0.225	0.200	0.222	0.207	0.172	0.202	0.193	0.162
11	69328130	A	T	2.608	0.257	0.239	0.268	0.248	0.209	0.235	0.223	0.234	0.227	0.230	0.239	0.237
11	69330983	G	A	3.027	0.020	0.010	0.023	0.008	0.028	0.013	0.020	0.014	0.007	0.019	0.009	0.009
11	69331418	C	T	3.304	0.011	0.004	0.015	0.004	0.017	0.006	0.012	0.008	0.005	0.009	5.20516 e-05	0.006
11	103614438	T	G	2.759	0.663	0.653	0.674	0.649	0.649	0.656	0.653	0.656	0.706	0.662	0.652	0.659
11	108267402	C	CA	2.712	0.422	0.407	0.419	0.403	0.421	0.404	0.415	0.398	0.450	0.402	0.414	0.409
11	111696440	T	C	2.615	0.770	0.764	0.763	0.775	0.756	0.766	0.757	0.760	0.773	0.786	0.747	0.738
11	116727936	A	T	2.608	0.189	0.175	0.187	0.188	0.176	0.183	0.197	0.168	0.177	0.194	0.188	0.192
11	122966626	A	G	2.618	0.381	0.374	0.388	0.393	0.381	0.370	0.384	0.371	0.438	0.349	0.415	0.361
11	129243417	T	G	2.578	0.972	0.975	0.973	0.978	0.971	0.971	0.971	0.975	0.986	0.982	0.980	0.980

11	129461016	A	G	2.847	0.561	0.533	0.557	0.532	0.543	0.540	0.546	0.546	0.546	0.556	0.603	0.572
12	293626	A	G	2.832	0.426	0.434	0.425	0.427	0.429	0.438	0.421	0.431	0.418	0.429	0.418	0.409
12	14413931	G	C	2.856	0.289	0.281	0.290	0.276	0.298	0.295	0.301	0.294	0.282	0.292	0.294	0.285
12	28149568	C	T	2.560	0.181	0.186	0.177	0.191	0.165	0.167	0.167	0.170	0.175	0.157	0.177	0.186
12	28174817	C	T	2.504	0.239	0.224	0.231	0.237	0.206	0.207	0.208	0.213	0.220	0.212	0.210	0.210
12	28347382	C	T	2.584	0.009	0.004	0.012	0.003	0.008	0.003	0.007	0.003	0.002	5.01292e-05	0.003	0.002
12	29140260	G	A	2.906	0.760	0.735	0.757	0.742	0.755	0.756	0.758	0.760	0.793	0.784	0.747	0.752
12	57146069	T	G	2.570	0.191	0.204	0.204	0.223	0.181	0.203	0.194	0.203	0.196	0.183	0.193	0.203
12	70798355	A	T	2.852	0.325	0.339	0.337	0.333	0.338	0.343	0.339	0.343	0.326	0.372	0.399	0.363
12	83064195	G	GA	2.914	0.137	0.151	0.131	0.157	0.136	0.157	0.139	0.158	0.130	0.142	0.164	0.172
12	85004551	C	T	2.816	0.140	0.138	0.155	0.137	0.127	0.134	0.135	0.142	0.170	0.158	0.115	0.132
12	96027759	A	G	2.502	0.280	0.246	0.250	0.251	0.248	0.229	0.232	0.228	0.201	0.227	0.223	0.222
12	103097887	C	T	2.875	0.005	0.000	0.007	0.001	0.006	0.001	0.006	0.002	0.008	0.003	0.000	0.002
12	111600134	G	T	2.603	0.003	0.002	0.006	0.001	0.005	0.001	0.004	0.001	0.001	0.000	0.000	0.000
12	115108136	T	C	2.851	0.414	0.439	0.396	0.426	0.448	0.439	0.442	0.441	0.465	0.431	0.420	0.442
12	115796577	A	G	2.607	0.315	0.292	0.317	0.274	0.290	0.275	0.286	0.264	0.276	0.272	0.274	0.263
12	115835836	T	C	2.514	0.286	0.274	0.315	0.272	0.263	0.262	0.264	0.240	0.280	0.276	0.281	0.220
12	120832146	C	T	2.866	0.076	0.071	0.077	0.066	0.079	0.069	0.074	0.068	0.076	0.052	0.073	0.077
13	32839990	G	A	2.839	0.006	0.000	0.006	0.000	0.005	0.000	0.005	0.000	0.007	0.000	0.002	0.000
13	32972626	A	T	3.700	0.000	0.000	0.001	0.000	0.001	0.000	5e-04	0.000	0.000	0.000	0.000	0.000
13	43501356	A	G	2.866	0.624	0.605	0.618	0.594	0.620	0.608	0.599	0.591	0.581	0.614	0.556	0.580
13	73806982	T	C	2.815	0.230	0.267	0.224	0.257	0.250	0.260	0.252	0.260	0.268	0.265	0.235	0.282
13	73960952	A	G	2.831	0.987	0.993	0.988	0.995	0.988	0.997	0.988	0.996	0.998	0.987	0.998	0.990

14	37128564	C	A	2.533	0.275	0.283	0.278	0.280	0.284	0.268	0.271	0.267	0.236	0.263	0.308	0.256
14	37228504	C	T	2.829	0.157	0.121	0.169	0.108	0.157	0.126	0.159	0.123	0.147	0.111	0.155	0.106
14	68660428	T	C	2.595	0.962	0.976	0.959	0.978	0.963	0.970	0.971	0.978	0.976	0.973	0.976	0.973
14	68979835	T	C	2.492	0.064	0.060	0.074	0.057	0.058	0.066	0.062	0.058	0.072	0.069	0.074	0.067
14	91751788	TC	T	2.826	0.489	0.495	0.499	0.479	0.477	0.510	0.465	0.468	0.491	0.516	0.482	0.495
14	91841069	A	G	2.865	0.150	0.136	0.139	0.126	0.156	0.139	0.146	0.133	0.134	0.149	0.115	0.121
14	93070286	C	T	2.570	0.021	0.004	0.018	0.003	0.016	0.004	0.015	0.006	0.008	0.003	0.011	0.003
14	105213978	T	G	2.831	0.644	0.678	0.639	0.656	0.661	0.665	0.655	0.660	0.599	0.681	0.610	0.682
15	46680811	C	A	2.273	0.000	4.14475e-05	0.001	0.000	0.000	5.59237e-05	0.000	5.08556e-05	2.40928e-05	2.19076e-05	1.0701e-05	0.000
15	50694306	A	G	2.609	0.467	0.477	0.461	0.499	0.469	0.497	0.487	0.501	0.525	0.481	0.477	0.493
15	66630569	G	A	2.622	0.794	0.799	0.811	0.828	0.784	0.818	0.811	0.805	0.849	0.834	0.839	0.802
15	67457698	A	G	2.949	0.001	0.000	0.002	0.001	0.002	0.001	0.003	0.001	0.002	0.000	0.002	0.003
15	75750383	T	C	2.610	0.411	0.393	0.435	0.407	0.382	0.402	0.381	0.397	0.396	0.379	0.389	0.400
15	91512267	G	T	2.567	0.484	0.488	0.480	0.508	0.460	0.468	0.465	0.475	0.522	0.458	0.470	0.480
15	100905819	A	C	2.563	0.211	0.231	0.204	0.228	0.216	0.238	0.216	0.232	0.228	0.256	0.219	0.226
16	4008542	CAAAAA	C	2.632	0.907	0.933	0.908	0.942	0.903	0.930	0.908	0.932	0.915	0.934	0.910	0.933
16	4106788	C	A	2.639	0.132	0.092	0.137	0.081	0.135	0.079	0.133	0.090	0.119	0.093	0.133	0.129
16	6963972	C	G	2.818	0.883	0.889	0.877	0.897	0.877	0.885	0.877	0.883	0.902	0.893	0.878	0.893
16	10706580	G	A	2.531	0.217	0.253	0.202	0.244	0.216	0.234	0.221	0.260	0.196	0.251	0.192	0.250
16	23007047	G	T	3.094	0.023	0.031	0.021	0.029	0.025	0.027	0.024	0.032	0.013	0.030	0.028	0.023
16	52538825	C	A	3.070	0.192	0.194	0.193	0.193	0.224	0.237	0.218	0.218	0.213	0.211	0.236	0.228
16	52599188	C	T	3.043	0.248	0.246	0.244	0.247	0.293	0.300	0.285	0.290	0.280	0.265	0.316	0.314
16	53809123	C	T	2.540	0.207	0.153	0.183	0.149	0.178	0.134	0.164	0.150	0.134	0.130	0.128	0.125

16	53861139	C	T	2.629	0.698	0.684	0.707	0.695	0.691	0.679	0.681	0.680	0.684	0.691	0.658	0.669
16	53861592	G	A	2.630	0.202	0.173	0.184	0.168	0.193	0.163	0.171	0.170	0.161	0.160	0.152	0.176
16	54682064	G	A	2.854	0.588	0.574	0.599	0.580	0.601	0.581	0.588	0.603	0.562	0.611	0.586	0.604
16	80648296	A	G	2.967	0.258	0.285	0.260	0.262	0.250	0.276	0.263	0.277	0.276	0.288	0.277	0.259
16	85145977	T	C	2.662	0.169	0.135	0.167	0.139	0.177	0.131	0.175	0.150	0.119	0.143	0.161	0.140
16	87086492	T	C	2.597	0.186	0.185	0.172	0.179	0.172	0.173	0.181	0.175	0.146	0.194	0.169	0.155
17	29168077	G	T	2.572	0.076	0.092	0.075	0.092	0.095	0.086	0.093	0.091	0.083	0.112	0.082	0.086
17	39251123	T	C	2.954	0.006	0.002	0.006	0.002	0.004	0.001	0.004	0.001	0.002	0.001	0.003	0.002
17	40127060	T	C	2.766	0.005	0.000	0.004	0.000	0.004	0.000	0.004	0.000	0.003	0.000	0.004	0.000
17	40485239	G	T	2.572	0.008	0.004	0.010	0.003	0.009	0.004	0.008	0.004	0.006	0.004	0.013	0.010
17	40744470	G	A	3.399	0.000	0.000	0.001	0.000	0.001	2.00873e-06	0.001	0.000	9.63713e-06	0.000	6.06388e-05	0.000
17	43212339	C	CT	2.843	0.113	0.118	0.117	0.124	0.114	0.123	0.113	0.116	0.117	0.126	0.113	0.134
17	44283858	G	A	2.579	0.003	0.001	0.002	0.002	0.004	0.001	0.003	0.001	0.000	0.000	0.000	0.000
17	53209774	A	C	2.519	0.287	0.293	0.269	0.292	0.269	0.272	0.282	0.289	0.251	0.293	0.253	0.261
17	77781725	A	G	2.614	0.404	0.408	0.390	0.412	0.410	0.396	0.406	0.408	0.370	0.409	0.373	0.423
18	11696613	C	T	2.619	0.047	0.016	0.046	0.016	0.040	0.016	0.035	0.017	0.029	0.016	0.037	0.020
18	20634253	C	T	2.610	0.463	0.445	0.456	0.457	0.457	0.460	0.446	0.445	0.458	0.474	0.492	0.446
18	24125857	T	C	2.816	0.644	0.669	0.646	0.670	0.657	0.675	0.643	0.665	0.619	0.666	0.667	0.679
18	24337424	C	G	2.848	0.737	0.724	0.730	0.707	0.740	0.729	0.736	0.728	0.713	0.730	0.716	0.722
18	24518050	AT	A	2.565	0.430	0.478	0.444	0.486	0.458	0.459	0.457	0.470	0.491	0.450	0.447	0.491
18	25407513	C	G	2.831	0.484	0.473	0.481	0.466	0.481	0.459	0.473	0.469	0.432	0.482	0.482	0.475
18	29981526	G	A	2.459	0.013	0.011	0.011	0.009	0.011	0.009	0.011	0.011	0.008	0.009	0.006	0.012
18	42411803	G	C	2.499	0.113	0.117	0.117	0.121	0.115	0.119	0.112	0.121	0.096	0.124	0.114	0.111

18	42888797	T	C	2.579	0.336	0.337	0.335	0.326	0.330	0.325	0.311	0.313	0.309	0.296	0.314	0.329
19	13249921	G	T	3.005	0.003	0.000	0.001	0.000	0.003	0.000	0.001	0.000	0.002	0.000	0.002	0.000
19	17393925	C	A	2.825	0.011	0.004	0.012	0.002	0.010	0.004	0.007	0.003	0.000	0.003	0.000	0.000
19	18569492	C	T	2.536	0.269	0.251	0.262	0.243	0.250	0.229	0.240	0.222	0.218	0.232	0.240	0.218
19	19517054	C	CGGGCG	2.842	0.429	0.389	0.424	0.387	0.417	0.392	0.413	0.393	0.454	0.418	0.374	0.386
19	44283031	T	C	2.898	0.163	0.128	0.171	0.122	0.145	0.133	0.146	0.129	0.102	0.114	0.127	0.137
19	46166073	T	C	2.624	0.410	0.369	0.418	0.368	0.431	0.364	0.425	0.368	0.395	0.388	0.366	0.385
19	55816678	C	T	2.624	0.107	0.086	0.112	0.085	0.115	0.087	0.110	0.093	0.065	0.091	0.069	0.101
20	5948227	G	A	2.942	0.001	0.000	0.001	0.000	0.002	0.000	0.001	0.000	0.000	0.002	0.000	0.000
20	11379842	T	C	2.969	0.998	1.000	0.997	1.000	0.998	1.000	0.997	1.000	0.998	1.000	0.998	1.000
20	41613706	C	G	2.807	0.846	0.838	0.837	0.840	0.832	0.839	0.824	0.828	0.818	0.848	0.850	0.839
20	52296849	G	A	2.843	0.320	0.315	0.309	0.309	0.338	0.317	0.334	0.332	0.304	0.307	0.325	0.339
21	16364756	T	G	2.906	0.141	0.179	0.143	0.176	0.147	0.178	0.150	0.169	0.157	0.166	0.175	0.155
21	16566350	A	G	2.890	0.142	0.106	0.144	0.103	0.135	0.116	0.134	0.106	0.115	0.122	0.129	0.101
21	16574455	C	A	2.539	0.126	0.104	0.129	0.099	0.119	0.103	0.107	0.106	0.104	0.107	0.128	0.101
21	47762932	G	A	3.002	0.015	0.002	0.016	0.005	0.017	0.005	0.020	0.006	0.020	0.003	0.017	0.008
22	19766137	C	T	2.622	0.573	0.563	0.570	0.596	0.561	0.557	0.582	0.563	0.620	0.603	0.613	0.535
22	29121087	A	G	3.326	4.1109e-06	0.000	0.000	0.000	0.000	0.000	8.75854e-06	0.000	0.000	0.000	0.000	0.000
22	29135543	G	A	2.908	0.089	0.089	0.085	0.081	0.090	0.083	0.092	0.091	0.082	0.073	0.095	0.111
22	29203724	C	T	3.161	0.001	0.000	0.001	0.000	0.001	0.000	0.001	0.000	2.16105e-05	0.000	0.000	0.000
22	29551872	A	G	2.322	1.000	1.000	0.999	1.000	1.000	1.000	1.000	1.000	1.000	1.000	1.000	1.000

22	38583315	AAAAG	AAAAGAAAG	2.596	0.267	0.252	0.266	0.251	0.267	0.259	0.252	0.253	0.222	0.236	0.234	0.285
22	39343916	T	A	2.834	0.452	0.489	0.445	0.479	0.504	0.498	0.492	0.499	0.465	0.519	0.450	0.486
22	40904707	CT	C	3.070	0.229	0.229	0.242	0.238	0.224	0.249	0.235	0.249	0.270	0.253	0.291	0.247
22	43433100	C	T	2.565	0.005	0.001	0.004	0.001	0.005	0.001	0.003	0.001	0.000	0.002	0.000	0.002
22	45319953	G	A	2.682	0.265	0.252	0.266	0.255	0.257	0.242	0.260	0.239	0.256	0.237	0.228	0.223
22	46283297	G	A	2.934	0.064	0.039	0.051	0.040	0.056	0.045	0.051	0.045	0.051	0.047	0.059	0.042
					**European-ancestry**											
					**Control**				**Invasive**				**In-situ**			
					**Age <50**		**Age≥50**		**Age <50**		**Age≥50**		**Age <50**		**Age≥50**	
**Chr**	**Position**	**A0**	**A1**	**Beta**	**ICOGS**	**OncoArray**	**ICOGS**	**OncoArray**	**ICOGS**	**OncoArray**	**ICOGS**	**OncoArray**	**ICOGS**	**OncoArray**	**ICOGS**	**OncoArray**
1	7917076	G	A	2.612	0.408	0.388	0.402	0.391	0.390	0.376	0.393	0.382	0.410	0.385	0.399	0.377
1	10566215	A	G	2.568	0.339	0.329	0.335	0.330	0.318	0.312	0.325	0.320	0.305	0.309	0.333	0.321
1	18807339	T	C	2.573	0.526	0.511	0.531	0.513	0.518	0.493	0.521	0.499	0.527	0.522	0.516	0.503
1	41380440	C	T	2.839	0.667	0.648	0.664	0.647	0.669	0.656	0.670	0.651	0.674	0.663	0.671	0.651
1	41389220	T	C	3.214	0.014	0.016	0.014	0.017	0.017	0.018	0.016	0.018	0.016	0.021	0.014	0.019
1	46670206	TC	T	2.845	0.308	0.299	0.303	0.300	0.305	0.304	0.312	0.305	0.288	0.303	0.308	0.305
1	51467096	CT	C	2.824	0.471	0.481	0.472	0.478	0.475	0.492	0.476	0.490	0.488	0.506	0.483	0.492
1	88156923	G	A	2.859	0.151	0.148	0.152	0.151	0.158	0.154	0.156	0.156	0.176	0.152	0.157	0.153
1	88428199	C	A	2.617	0.251	0.248	0.249	0.248	0.236	0.235	0.240	0.238	0.238	0.221	0.245	0.249
1	100880328	A	T	2.824	0.404	0.411	0.400	0.410	0.409	0.412	0.408	0.412	0.392	0.409	0.398	0.400
1	110198129	CAAA	C	2.849	0.775	0.777	0.772	0.777	0.782	0.781	0.783	0.781	0.789	0.783	0.789	0.787

1	114445880	G	A	2.898	0.173	0.168	0.164	0.169	0.177	0.173	0.173	0.172	0.172	0.168	0.168	0.167
1	118141492	A	C	2.847	0.257	0.267	0.261	0.263	0.276	0.279	0.267	0.273	0.294	0.272	0.272	0.271
1	120257110	T	C	2.827	0.534	0.533	0.529	0.530	0.544	0.537	0.538	0.537	0.530	0.537	0.537	0.537
1	121280613	A	G	2.981	0.402	0.406	0.405	0.405	0.430	0.430	0.428	0.437	0.421	0.421	0.410	0.430
1	121287994	A	G	2.547	0.100	0.103	0.101	0.104	0.097	0.102	0.094	0.100	0.097	0.104	0.100	0.102
1	145604302	C	CT	2.614	0.352	0.352	0.355	0.351	0.343	0.337	0.345	0.340	0.338	0.348	0.349	0.340
1	149906413	T	C	2.876	0.400	0.401	0.402	0.402	0.415	0.411	0.410	0.412	0.421	0.421	0.408	0.413
1	155556971	G	A	2.861	0.231	0.229	0.232	0.231	0.235	0.233	0.241	0.236	0.236	0.225	0.245	0.241
1	168171052	CA	C	2.545	0.097	0.110	0.096	0.107	0.092	0.107	0.092	0.105	0.085	0.109	0.095	0.108
1	172328767	T	TA	2.605	0.340	0.331	0.332	0.329	0.318	0.318	0.327	0.325	0.322	0.326	0.323	0.325
1	201437832	C	T	2.992	0.054	0.060	0.053	0.056	0.057	0.064	0.056	0.063	0.059	0.065	0.054	0.061
1	202184600	C	T	2.701	0.414	0.405	0.413	0.403	0.406	0.396	0.407	0.396	0.406	0.390	0.403	0.398
1	203770448	T	A	2.861	0.270	0.271	0.270	0.272	0.282	0.279	0.279	0.280	0.275	0.277	0.279	0.280
1	204502514	T	TTCTGAA ACAGGG	2.634	0.817	0.794	0.812	0.804	0.805	0.792	0.809	0.798	0.794	0.786	0.793	0.796
1	208076291	G	A	2.622	0.338	0.338	0.333	0.334	0.326	0.328	0.327	0.329	0.330	0.317	0.329	0.318
1	217053815	T	G	2.837	0.320	0.333	0.324	0.329	0.334	0.330	0.331	0.329	0.315	0.332	0.323	0.329
1	217220574	G	A	2.604	0.207	0.215	0.206	0.209	0.208	0.208	0.202	0.204	0.208	0.211	0.208	0.210
1	220671050	C	T	2.837	0.224	0.242	0.222	0.241	0.230	0.250	0.232	0.248	0.220	0.248	0.218	0.238
1	242034263	A	G	3.169	0.031	0.030	0.031	0.032	0.038	0.036	0.035	0.034	0.034	0.035	0.034	0.037
2	10138983	T	C	2.893	0.113	0.115	0.115	0.117	0.115	0.125	0.118	0.122	0.127	0.117	0.119	0.123
2	19315675	T	A	2.631	0.567	0.559	0.564	0.563	0.545	0.547	0.556	0.551	0.519	0.531	0.550	0.537
2	25129473	A	G	2.607	0.411	0.413	0.415	0.410	0.400	0.403	0.405	0.400	0.412	0.405	0.415	0.400
2	29179452	G	C	2.700	0.242	0.229	0.236	0.230	0.231	0.229	0.234	0.229	0.233	0.227	0.240	0.224

2	29615233	T	C	2.607	0.253	0.260	0.261	0.257	0.261	0.258	0.256	0.258	0.249	0.276	0.246	0.254
2	39699510	C	CT	2.613	0.484	0.463	0.482	0.465	0.477	0.460	0.480	0.463	0.480	0.468	0.486	0.461
2	70172587	G	A	2.611	0.306	0.277	0.304	0.282	0.291	0.269	0.298	0.268	0.279	0.267	0.292	0.275
2	88358825	G	C	2.853	0.330	0.311	0.326	0.309	0.334	0.309	0.332	0.312	0.331	0.312	0.335	0.312
2	121058254	A	G	2.630	0.685	0.703	0.693	0.703	0.682	0.698	0.684	0.699	0.693	0.707	0.683	0.704
2	121089731	T	C	2.607	0.197	0.197	0.196	0.192	0.192	0.191	0.191	0.191	0.189	0.193	0.190	0.188
2	121159205	G	A	2.604	0.333	0.350	0.341	0.356	0.329	0.341	0.329	0.341	0.326	0.328	0.322	0.332
2	121246568	T	C	3.017	0.902	0.898	0.901	0.897	0.907	0.904	0.908	0.904	0.913	0.902	0.912	0.907
2	172974566	C	G	2.596	0.475	0.472	0.479	0.478	0.461	0.459	0.465	0.461	0.467	0.471	0.464	0.466
2	174212910	A	G	2.890	0.849	0.845	0.840	0.842	0.853	0.852	0.847	0.851	0.849	0.843	0.844	0.843
2	192381934	C	T	2.807	0.872	0.855	0.874	0.862	0.876	0.862	0.872	0.861	0.868	0.856	0.878	0.866
2	202204741	T	C	2.591	0.723	0.724	0.725	0.723	0.706	0.704	0.714	0.710	0.716	0.710	0.723	0.704
2	217920769	G	T	2.403	0.514	0.496	0.507	0.504	0.477	0.469	0.469	0.465	0.497	0.472	0.481	0.470
2	217955896	GA	G	2.265	0.042	0.035	0.040	0.038	0.033	0.031	0.034	0.030	0.026	0.031	0.035	0.034
2	218292158	C	G	2.527	0.726	0.730	0.727	0.731	0.709	0.720	0.712	0.716	0.692	0.702	0.715	0.717
2	218714845	G	A	2.606	0.394	0.395	0.393	0.395	0.375	0.379	0.385	0.383	0.385	0.378	0.388	0.381
2	241388857	C	A	2.421	0.980	0.978	0.978	0.976	0.975	0.977	0.978	0.975	0.975	0.974	0.977	0.974
3	4742251	A	G	2.897	0.397	0.385	0.397	0.388	0.412	0.389	0.406	0.390	0.421	0.382	0.412	0.389
3	27353716	C	A	2.938	0.523	0.531	0.521	0.524	0.558	0.559	0.547	0.552	0.566	0.571	0.550	0.547
3	27388664	C	G	2.862	0.273	0.278	0.265	0.271	0.290	0.302	0.285	0.296	0.291	0.307	0.284	0.290
3	29294845	C	T	2.410	0.016	0.015	0.015	0.017	0.014	0.015	0.013	0.015	0.015	0.020	0.014	0.015
3	30684907	C	T	2.889	0.300	0.299	0.295	0.298	0.302	0.306	0.310	0.308	0.293	0.290	0.292	0.290
3	46888198	T	C	2.516	0.103	0.103	0.097	0.103	0.091	0.097	0.091	0.096	0.090	0.101	0.091	0.098

3	49709912	C	CT	2.622	0.295	0.290	0.286	0.284	0.284	0.280	0.283	0.286	0.284	0.280	0.286	0.283
3	55970777	A	AT	2.429	0.021	0.029	0.020	0.032	0.017	0.026	0.019	0.028	0.015	0.022	0.019	0.028
3	59373745	C	T	2.615	0.417	0.431	0.419	0.429	0.409	0.418	0.414	0.419	0.403	0.410	0.415	0.415
3	63887449	T	TTG	2.906	0.128	0.130	0.131	0.130	0.135	0.139	0.136	0.136	0.152	0.139	0.144	0.141
3	71620370	T	G	2.620	0.663	0.641	0.660	0.643	0.639	0.628	0.652	0.632	0.643	0.633	0.657	0.627
3	87037543	A	G	2.535	0.096	0.090	0.094	0.093	0.085	0.086	0.088	0.086	0.085	0.093	0.094	0.089
3	99403877	G	A	2.620	0.476	0.484	0.486	0.485	0.469	0.478	0.480	0.478	0.476	0.488	0.481	0.481
3	141112859	CTT	C	2.877	0.439	0.415	0.430	0.422	0.439	0.421	0.440	0.425	0.420	0.433	0.445	0.417
3	172285237	G	A	2.838	0.221	0.216	0.221	0.214	0.225	0.221	0.225	0.222	0.215	0.223	0.222	0.216
3	189774456	C	T	2.594	0.221	0.225	0.219	0.222	0.213	0.215	0.213	0.218	0.214	0.214	0.204	0.217
4	38784633	G	T	2.858	0.248	0.249	0.240	0.246	0.245	0.256	0.249	0.255	0.255	0.269	0.243	0.270
4	84370124	TAA	TA	2.598	0.536	0.533	0.532	0.535	0.514	0.517	0.525	0.521	0.537	0.524	0.524	0.515
4	89240476	G	A	2.817	0.438	0.446	0.439	0.440	0.447	0.454	0.448	0.449	0.443	0.449	0.447	0.440
4	92594859	TTCTTTC	T	2.612	0.449	0.444	0.455	0.440	0.450	0.436	0.449	0.440	0.463	0.468	0.450	0.436
4	106069013	G	T	2.853	0.224	0.225	0.223	0.229	0.232	0.233	0.233	0.233	0.229	0.228	0.211	0.228
4	126752992	A	AAT	2.620	0.513	0.518	0.512	0.515	0.502	0.505	0.503	0.508	0.493	0.514	0.500	0.504
4	143467195	C	T	2.572	0.118	0.114	0.112	0.113	0.109	0.106	0.108	0.107	0.113	0.105	0.114	0.109
4	151218296	CATATTT	C	2.828	0.654	0.648	0.651	0.654	0.661	0.657	0.658	0.655	0.647	0.659	0.648	0.653
4	175842495	G	A	2.495	0.115	0.115	0.120	0.117	0.107	0.106	0.108	0.107	0.098	0.110	0.112	0.105
4	175847436	C	A	2.816	0.339	0.337	0.339	0.344	0.345	0.350	0.354	0.351	0.356	0.353	0.348	0.351
4	187503758	A	T	2.819	0.444	0.448	0.442	0.447	0.452	0.453	0.452	0.456	0.445	0.461	0.450	0.446
5	345109	T	C	2.967	0.050	0.058	0.055	0.054	0.063	0.059	0.059	0.060	0.064	0.055	0.058	0.057
5	1279790	C	T	2.897	0.262	0.266	0.256	0.259	0.277	0.275	0.268	0.265	0.275	0.265	0.275	0.259

5	1296255	A	AG	2.577	0.301	0.301	0.308	0.310	0.292	0.293	0.289	0.295	0.308	0.281	0.295	0.290
5	1353077	T	C	3.215	0.012	0.012	0.012	0.012	0.011	0.014	0.013	0.014	0.011	0.013	0.013	0.015
5	2777029	G	A	2.829	0.419	0.413	0.417	0.416	0.415	0.422	0.426	0.422	0.405	0.416	0.421	0.417
5	16231194	G	C	2.607	0.558	0.555	0.562	0.560	0.543	0.545	0.552	0.547	0.557	0.550	0.553	0.550
5	32579616	TCA	T	2.821	0.464	0.481	0.462	0.484	0.470	0.490	0.473	0.491	0.473	0.496	0.463	0.499
5	44508264	G	GT	2.433	0.114	0.122	0.115	0.124	0.114	0.120	0.111	0.117	0.112	0.111	0.113	0.121
5	44619502	A	G	2.449	0.152	0.161	0.158	0.153	0.146	0.149	0.144	0.145	0.135	0.154	0.144	0.139
5	44649944	C	T	2.859	0.601	0.603	0.602	0.599	0.607	0.617	0.609	0.613	0.604	0.610	0.618	0.614
5	44706498	A	G	2.860	0.257	0.248	0.251	0.248	0.273	0.268	0.277	0.276	0.288	0.276	0.279	0.281
5	44853593	G	C	2.630	0.317	0.309	0.314	0.310	0.303	0.298	0.303	0.300	0.318	0.292	0.307	0.305
5	52679539	C	CA	2.883	0.104	0.102	0.099	0.099	0.106	0.107	0.105	0.105	0.111	0.110	0.102	0.101
5	55662540	C	CT	2.599	0.353	0.362	0.355	0.362	0.349	0.360	0.348	0.358	0.342	0.349	0.345	0.358
5	55965167	C	T	2.830	0.549	0.559	0.557	0.557	0.566	0.573	0.563	0.571	0.570	0.589	0.559	0.559
5	56023083	T	G	3.147	0.163	0.160	0.159	0.159	0.188	0.182	0.183	0.183	0.191	0.191	0.198	0.185
5	56042972	C	T	2.975	0.051	0.054	0.051	0.052	0.066	0.065	0.063	0.061	0.067	0.075	0.073	0.065
5	56045081	T	C	2.573	0.163	0.163	0.165	0.166	0.153	0.149	0.156	0.152	0.132	0.148	0.144	0.147
5	58241712	C	T	2.605	0.578	0.578	0.583	0.576	0.571	0.571	0.575	0.574	0.570	0.579	0.582	0.569
5	71965007	G	A	2.611	0.242	0.257	0.247	0.258	0.237	0.250	0.237	0.249	0.244	0.257	0.245	0.246
5	73234583	T	C	2.623	0.337	0.322	0.337	0.321	0.326	0.314	0.332	0.315	0.351	0.318	0.333	0.324
5	77155397	GT	G	2.612	0.335	0.344	0.336	0.346	0.328	0.346	0.328	0.344	0.320	0.361	0.331	0.349
5	79180995	G	GA	2.810	0.177	0.173	0.180	0.177	0.185	0.174	0.186	0.174	0.196	0.173	0.180	0.175
5	81512947	TA	T	2.565	0.237	0.246	0.246	0.250	0.231	0.239	0.231	0.243	0.233	0.240	0.237	0.244
5	90789470	G	A	2.573	0.167	0.156	0.170	0.159	0.158	0.147	0.162	0.149	0.162	0.162	0.159	0.152

5	104300273	G	T	2.592	0.176	0.179	0.175	0.175	0.174	0.177	0.169	0.178	0.146	0.188	0.173	0.180
5	122478676	C	A	2.617	0.740	0.741	0.741	0.745	0.736	0.733	0.735	0.742	0.729	0.759	0.749	0.739
5	122705244	C	T	3.001	0.032	0.032	0.028	0.030	0.030	0.032	0.032	0.032	0.029	0.024	0.026	0.028
5	131640536	A	G	2.829	0.541	0.548	0.535	0.540	0.554	0.553	0.547	0.550	0.569	0.566	0.556	0.552
5	132407058	C	T	2.617	0.240	0.241	0.240	0.246	0.238	0.235	0.232	0.234	0.223	0.236	0.230	0.238
5	158244083	C	T	2.546	0.563	0.562	0.565	0.568	0.549	0.548	0.549	0.551	0.549	0.538	0.547	0.548
5	169591460	T	C	2.835	0.333	0.340	0.331	0.333	0.342	0.345	0.336	0.347	0.353	0.355	0.334	0.360
5	173358154	G	A	2.821	0.419	0.409	0.413	0.406	0.425	0.423	0.413	0.418	0.418	0.415	0.419	0.413
5	176134882	T	C	2.821	0.536	0.548	0.534	0.541	0.538	0.548	0.541	0.547	0.517	0.535	0.531	0.547
6	13713366	G	C	2.576	0.569	0.568	0.569	0.570	0.556	0.553	0.556	0.559	0.557	0.549	0.565	0.560
6	16399557	C	T	2.621	0.375	0.337	0.371	0.327	0.365	0.323	0.367	0.320	0.366	0.313	0.365	0.327
6	18783140	G	A	2.810	0.614	0.624	0.619	0.621	0.613	0.619	0.623	0.623	0.598	0.625	0.616	0.622
6	20537845	CA	C	2.616	0.472	0.479	0.474	0.471	0.460	0.470	0.464	0.465	0.473	0.486	0.464	0.461
6	21923810	T	C	2.634	0.437	0.429	0.426	0.431	0.419	0.430	0.423	0.421	0.416	0.429	0.428	0.424
6	27425644	G	C	2.532	0.091	0.085	0.088	0.083	0.081	0.072	0.079	0.075	0.090	0.073	0.089	0.075
6	43227141	G	A	2.555	0.101	0.095	0.098	0.099	0.094	0.089	0.097	0.093	0.094	0.087	0.095	0.093
6	82263549	AAT	A	2.854	0.425	0.424	0.424	0.425	0.427	0.437	0.437	0.437	0.419	0.434	0.435	0.436
6	85912194	CAA	C	2.942	0.055	0.061	0.059	0.060	0.062	0.062	0.064	0.065	0.067	0.066	0.062	0.061
6	87803819	T	C	2.827	0.284	0.280	0.282	0.281	0.283	0.280	0.290	0.281	0.284	0.264	0.286	0.285
6	130341728	C	CT	2.853	0.705	0.714	0.710	0.711	0.722	0.719	0.718	0.721	0.706	0.729	0.720	0.720
6	149595505	T	C	2.595	0.201	0.204	0.200	0.204	0.190	0.201	0.193	0.199	0.195	0.199	0.189	0.198
6	151949806	A	C	2.924	0.310	0.311	0.311	0.308	0.335	0.337	0.326	0.326	0.342	0.336	0.332	0.321
6	151955914	A	G	3.177	0.066	0.072	0.068	0.070	0.085	0.091	0.078	0.082	0.095	0.082	0.081	0.083

6	152022664	CAAAAAAA	C	2.756	0.607	0.613	0.611	0.610	0.630	0.634	0.619	0.625	0.622	0.615	0.622	0.628
6	152023191	G	A	2.900	0.402	0.398	0.400	0.396	0.415	0.410	0.411	0.406	0.394	0.401	0.401	0.411
6	152055978	A	T	2.935	0.069	0.062	0.062	0.063	0.072	0.072	0.071	0.068	0.067	0.068	0.069	0.067
6	152432902	C	T	2.907	0.530	0.514	0.521	0.517	0.534	0.530	0.540	0.528	0.533	0.528	0.545	0.526
6	169006947	C	G	2.637	0.509	0.517	0.508	0.517	0.501	0.519	0.505	0.517	0.506	0.518	0.515	0.522
6	170332621	T	C	2.824	0.627	0.622	0.618	0.618	0.628	0.625	0.626	0.620	0.643	0.629	0.621	0.629
7	21940960	A	G	2.597	0.357	0.354	0.358	0.350	0.353	0.341	0.349	0.346	0.327	0.351	0.358	0.366
7	25569548	C	T	2.592	0.155	0.167	0.152	0.164	0.147	0.161	0.148	0.163	0.143	0.155	0.148	0.157
7	28869017	G	A	2.571	0.102	0.108	0.105	0.106	0.100	0.106	0.104	0.107	0.127	0.109	0.108	0.103
7	55192256	A	C	2.627	0.561	0.548	0.561	0.555	0.544	0.536	0.555	0.543	0.564	0.557	0.563	0.537
7	91459189	A	ATT	2.847	0.339	0.326	0.341	0.330	0.355	0.338	0.353	0.338	0.347	0.355	0.360	0.350
7	94113799	T	C	2.846	0.281	0.276	0.283	0.282	0.298	0.290	0.288	0.288	0.299	0.288	0.294	0.300
7	98005235	G	A	2.597	0.171	0.165	0.165	0.164	0.159	0.155	0.162	0.156	0.150	0.152	0.169	0.165
7	99948655	T	G	2.837	0.199	0.214	0.203	0.210	0.212	0.214	0.208	0.217	0.205	0.211	0.197	0.218
7	101552440	G	A	2.572	0.119	0.122	0.117	0.127	0.110	0.120	0.110	0.120	0.107	0.121	0.118	0.114
7	102481842	T	C	2.837	0.345	0.341	0.338	0.340	0.343	0.350	0.351	0.349	0.331	0.341	0.353	0.342
7	130656911	C	T	2.595	0.372	0.374	0.379	0.375	0.361	0.357	0.363	0.360	0.356	0.362	0.371	0.370
7	130674481	G	A	2.836	0.301	0.297	0.312	0.296	0.319	0.306	0.314	0.306	0.303	0.294	0.315	0.304
7	139943702	CT	C	2.886	0.522	0.535	0.519	0.535	0.528	0.554	0.528	0.552	0.542	0.559	0.520	0.544
7	144048902	G	T	2.573	0.231	0.231	0.229	0.228	0.219	0.221	0.217	0.225	0.225	0.214	0.222	0.231
8	170692	T	C	2.854	0.232	0.222	0.234	0.225	0.244	0.225	0.240	0.227	0.238	0.223	0.243	0.215
8	17787610	CT	C	2.620	0.636	0.622	0.635	0.625	0.621	0.615	0.628	0.616	0.629	0.627	0.620	0.611
8	23447496	A	G	2.617	0.650	0.645	0.640	0.645	0.637	0.642	0.640	0.639	0.626	0.645	0.633	0.640

8	23663653	C	A	2.812	0.413	0.410	0.412	0.407	0.410	0.407	0.417	0.409	0.410	0.427	0.423	0.412
8	29509616	A	C	2.564	0.675	0.673	0.677	0.678	0.665	0.662	0.661	0.661	0.646	0.659	0.665	0.654
8	36858483	A	G	2.526	0.169	0.181	0.169	0.177	0.167	0.169	0.163	0.173	0.170	0.161	0.163	0.177
8	76230943	A	G	2.940	0.822	0.825	0.825	0.827	0.845	0.844	0.833	0.837	0.842	0.854	0.839	0.839
8	76333056	C	T	3.063	0.080	0.088	0.084	0.087	0.097	0.101	0.093	0.095	0.106	0.099	0.095	0.098
8	76378165	G	T	2.616	0.361	0.357	0.362	0.358	0.350	0.357	0.350	0.356	0.362	0.355	0.357	0.345
8	102483100	T	C	2.890	0.097	0.098	0.098	0.095	0.107	0.106	0.106	0.102	0.103	0.093	0.105	0.104
8	106358620	A	T	2.530	0.102	0.099	0.102	0.102	0.099	0.097	0.096	0.094	0.104	0.101	0.098	0.091
8	117209548	A	G	2.609	0.648	0.644	0.656	0.650	0.637	0.635	0.640	0.637	0.643	0.641	0.653	0.647
8	120862186	A	G	2.869	0.133	0.131	0.129	0.134	0.137	0.133	0.136	0.134	0.147	0.137	0.137	0.129
8	124563705	T	C	2.854	0.162	0.145	0.161	0.147	0.165	0.150	0.165	0.155	0.174	0.167	0.168	0.153
8	124571581	G	A	2.814	0.414	0.413	0.415	0.418	0.424	0.426	0.426	0.427	0.426	0.429	0.420	0.419
8	124739913	T	G	2.851	0.400	0.401	0.401	0.399	0.414	0.407	0.410	0.409	0.437	0.415	0.419	0.405
8	128213561	C	CA	2.606	0.403	0.424	0.408	0.417	0.392	0.398	0.403	0.405	0.377	0.402	0.396	0.399
8	128370949	C	G	2.905	0.399	0.411	0.396	0.398	0.429	0.434	0.422	0.424	0.431	0.454	0.407	0.427
8	128372172	A	G	2.891	0.540	0.551	0.541	0.539	0.574	0.578	0.568	0.569	0.569	0.583	0.546	0.563
8	129199566	G	A	2.896	0.157	0.172	0.163	0.170	0.179	0.191	0.173	0.183	0.190	0.191	0.181	0.181
8	143669254	A	G	2.627	0.370	0.338	0.375	0.336	0.364	0.336	0.365	0.333	0.383	0.350	0.377	0.335
9	6880263	A	G	2.816	0.265	0.286	0.271	0.284	0.276	0.289	0.275	0.294	0.287	0.311	0.267	0.281
9	21964882	CAAAA	C	2.876	0.308	0.321	0.321	0.319	0.335	0.338	0.328	0.337	0.350	0.351	0.327	0.340
9	22041998	C	G	2.799	0.140	0.137	0.140	0.138	0.152	0.147	0.143	0.142	0.146	0.142	0.148	0.147
9	36928288	T	C	2.788	0.537	0.542	0.540	0.536	0.540	0.538	0.542	0.543	0.561	0.561	0.536	0.539
9	87782211	T	C	2.820	0.513	0.510	0.505	0.512	0.517	0.516	0.517	0.512	0.521	0.510	0.518	0.504

9	98362587	T	C	2.884	0.093	0.091	0.095	0.095	0.098	0.094	0.100	0.098	0.092	0.085	0.096	0.097
9	110303808	TAA	T	2.953	0.189	0.205	0.190	0.202	0.206	0.227	0.204	0.222	0.196	0.222	0.188	0.212
9	110837073	A	G	3.073	0.060	0.063	0.061	0.062	0.071	0.072	0.066	0.068	0.074	0.072	0.072	0.067
9	110837176	C	T	2.908	0.175	0.181	0.177	0.177	0.185	0.181	0.188	0.183	0.176	0.178	0.184	0.181
9	110849525	G	T	2.761	0.600	0.599	0.602	0.597	0.624	0.622	0.622	0.615	0.626	0.615	0.620	0.616
9	110885479	C	T	2.979	0.613	0.625	0.614	0.616	0.647	0.646	0.642	0.643	0.636	0.634	0.629	0.646
9	119313486	A	G	2.598	0.407	0.409	0.401	0.406	0.389	0.393	0.396	0.396	0.393	0.381	0.391	0.400
9	129424719	A	G	2.618	0.458	0.459	0.456	0.457	0.449	0.453	0.446	0.448	0.457	0.453	0.454	0.449
9	136146597	C	T	2.832	0.280	0.279	0.277	0.272	0.276	0.272	0.288	0.279	0.266	0.275	0.278	0.274
10	5794652	A	G	2.852	0.222	0.216	0.221	0.217	0.227	0.220	0.227	0.220	0.238	0.217	0.219	0.215
10	13892298	G	A	2.823	0.442	0.438	0.438	0.439	0.449	0.446	0.444	0.445	0.434	0.457	0.442	0.441
10	22032942	A	G	2.569	0.718	0.707	0.716	0.712	0.697	0.696	0.699	0.696	0.714	0.698	0.709	0.702
10	22477776	ACC	A	3.267	0.020	0.020	0.019	0.020	0.022	0.023	0.023	0.023	0.022	0.022	0.021	0.022
10	22861490	A	C	2.979	0.942	0.935	0.941	0.939	0.943	0.940	0.944	0.939	0.950	0.941	0.945	0.937
10	38523626	C	A	2.833	0.370	0.368	0.368	0.370	0.375	0.379	0.372	0.377	0.393	0.386	0.375	0.382
10	64299890	A	G	2.397	0.162	0.158	0.159	0.158	0.144	0.147	0.142	0.146	0.132	0.141	0.135	0.144
10	64819996	G	T	2.853	0.200	0.195	0.197	0.198	0.201	0.200	0.204	0.199	0.206	0.200	0.204	0.190
10	71335574	C	T	2.613	0.309	0.317	0.310	0.319	0.302	0.306	0.302	0.313	0.320	0.309	0.297	0.314
10	80851257	G	T	2.516	0.612	0.611	0.610	0.616	0.594	0.594	0.596	0.597	0.584	0.590	0.597	0.594
10	80886726	A	G	2.942	0.168	0.163	0.160	0.162	0.174	0.177	0.172	0.176	0.170	0.180	0.168	0.180
10	95292187	CAA	C	2.586	0.825	0.821	0.822	0.821	0.819	0.817	0.818	0.819	0.820	0.821	0.809	0.823
10	114777670	C	T	2.853	0.456	0.470	0.455	0.460	0.472	0.479	0.468	0.472	0.485	0.469	0.469	0.472
10	115128491	T	C	2.566	0.781	0.784	0.789	0.787	0.770	0.772	0.775	0.775	0.780	0.771	0.789	0.783

10	123095209	G	A	2.580	0.318	0.330	0.328	0.325	0.314	0.316	0.320	0.317	0.314	0.297	0.318	0.331
10	123340107	A	G	3.199	0.062	0.064	0.061	0.066	0.056	0.061	0.056	0.058	0.058	0.062	0.056	0.060
10	123340431	GC	G	2.195	0.593	0.592	0.600	0.600	0.533	0.530	0.548	0.538	0.522	0.515	0.541	0.531
10	123349324	A	T	2.161	0.047	0.046	0.046	0.049	0.039	0.043	0.039	0.040	0.044	0.044	0.040	0.041
11	433617	T	C	2.605	0.799	0.795	0.806	0.793	0.807	0.796	0.802	0.794	0.801	0.790	0.816	0.792
11	803017	A	G	2.848	0.528	0.520	0.517	0.519	0.533	0.526	0.527	0.521	0.503	0.532	0.530	0.533
11	1895708	C	A	2.526	0.396	0.391	0.393	0.391	0.380	0.376	0.375	0.374	0.378	0.357	0.380	0.383
11	18664241	T	G	2.850	0.700	0.727	0.709	0.730	0.713	0.739	0.715	0.737	0.730	0.730	0.710	0.734
11	42844441	C	T	2.630	0.337	0.325	0.334	0.331	0.326	0.323	0.332	0.325	0.327	0.330	0.338	0.335
11	44368892	G	A	2.824	0.544	0.552	0.545	0.548	0.550	0.554	0.550	0.557	0.547	0.555	0.544	0.548
11	46318032	C	G	2.529	0.055	0.066	0.056	0.065	0.057	0.066	0.055	0.064	0.061	0.060	0.060	0.068
11	65553492	C	A	2.839	0.184	0.189	0.186	0.186	0.198	0.193	0.195	0.191	0.193	0.204	0.194	0.191
11	65572431	G	A	2.627	0.480	0.486	0.485	0.486	0.465	0.482	0.471	0.480	0.471	0.481	0.477	0.486
11	69328130	A	T	2.608	0.223	0.217	0.218	0.215	0.204	0.204	0.203	0.201	0.227	0.204	0.214	0.198
11	69330983	G	A	3.027	0.125	0.124	0.120	0.125	0.145	0.149	0.149	0.148	0.138	0.154	0.137	0.139
11	69331418	C	T	3.304	0.077	0.073	0.073	0.075	0.096	0.095	0.097	0.094	0.085	0.100	0.080	0.082
11	103614438	T	G	2.759	0.657	0.658	0.653	0.657	0.657	0.660	0.659	0.659	0.652	0.657	0.659	0.663
11	108267402	C	CA	2.712	0.395	0.416	0.406	0.411	0.414	0.426	0.407	0.422	0.425	0.441	0.416	0.431
11	111696440	T	C	2.615	0.622	0.623	0.622	0.620	0.614	0.619	0.614	0.618	0.607	0.622	0.611	0.613
11	116727936	A	T	2.608	0.195	0.197	0.195	0.202	0.191	0.196	0.194	0.198	0.182	0.183	0.194	0.192
11	122966626	A	G	2.618	0.275	0.292	0.271	0.291	0.270	0.288	0.270	0.286	0.264	0.299	0.269	0.293
11	129243417	T	G	2.578	0.855	0.858	0.858	0.861	0.853	0.854	0.855	0.854	0.844	0.860	0.852	0.858
11	129461016	A	G	2.847	0.584	0.601	0.593	0.598	0.611	0.616	0.600	0.611	0.608	0.609	0.596	0.606

12	293626	A	G	2.832	0.407	0.367	0.407	0.374	0.413	0.374	0.414	0.375	0.409	0.357	0.401	0.381
12	14413931	G	C	2.856	0.259	0.259	0.257	0.261	0.268	0.275	0.266	0.271	0.282	0.277	0.273	0.281
12	28149568	C	T	2.560	0.123	0.116	0.122	0.120	0.101	0.102	0.107	0.104	0.104	0.103	0.112	0.109
12	28174817	C	T	2.504	0.242	0.237	0.240	0.245	0.215	0.219	0.221	0.222	0.221	0.231	0.219	0.225
12	28347382	C	T	2.584	0.222	0.212	0.220	0.216	0.202	0.200	0.208	0.201	0.207	0.207	0.218	0.215
12	29140260	G	A	2.906	0.918	0.913	0.915	0.913	0.924	0.923	0.918	0.918	0.926	0.918	0.927	0.921
12	57146069	T	G	2.570	0.102	0.105	0.105	0.103	0.095	0.104	0.099	0.101	0.102	0.095	0.098	0.102
12	70798355	A	T	2.852	0.184	0.183	0.176	0.182	0.190	0.182	0.181	0.184	0.179	0.188	0.192	0.182
12	83064195	G	GA	2.914	0.101	0.100	0.102	0.099	0.106	0.105	0.105	0.104	0.097	0.113	0.109	0.103
12	85004551	C	T	2.816	0.487	0.495	0.496	0.495	0.506	0.512	0.498	0.508	0.517	0.501	0.496	0.501
12	96027759	A	G	2.502	0.301	0.295	0.300	0.297	0.284	0.284	0.278	0.279	0.288	0.273	0.282	0.285
12	103097887	C	T	2.875	0.113	0.119	0.113	0.118	0.115	0.117	0.120	0.122	0.111	0.111	0.119	0.121
12	111600134	G	T	2.603	0.369	0.363	0.370	0.371	0.364	0.367	0.364	0.368	0.354	0.387	0.356	0.365
12	115108136	T	C	2.851	0.263	0.270	0.255	0.258	0.265	0.273	0.267	0.272	0.274	0.279	0.267	0.284
12	115796577	A	G	2.607	0.184	0.198	0.192	0.194	0.187	0.195	0.183	0.190	0.196	0.200	0.189	0.197
12	115835836	T	C	2.514	0.415	0.420	0.418	0.419	0.396	0.392	0.399	0.400	0.393	0.380	0.403	0.402
12	120832146	C	T	2.866	0.154	0.156	0.155	0.159	0.159	0.166	0.160	0.162	0.142	0.159	0.160	0.163
13	32839990	G	A	2.839	0.017	0.018	0.018	0.019	0.020	0.023	0.020	0.020	0.019	0.017	0.021	0.024
13	32972626	A	T	3.700	0.008	0.009	0.008	0.008	0.012	0.014	0.010	0.010	0.014	0.010	0.012	0.011
13	43501356	A	G	2.866	0.834	0.830	0.827	0.830	0.837	0.832	0.834	0.836	0.842	0.839	0.831	0.829
13	73806982	T	C	2.815	0.327	0.322	0.319	0.318	0.330	0.321	0.325	0.318	0.314	0.329	0.322	0.313
13	73960952	A	G	2.831	0.769	0.765	0.764	0.763	0.766	0.767	0.772	0.768	0.766	0.761	0.766	0.760
14	37128564	C	A	2.533	0.209	0.209	0.212	0.211	0.199	0.201	0.194	0.199	0.177	0.209	0.204	0.201

14	37228504	C	T	2.829	0.408	0.447	0.414	0.442	0.418	0.451	0.423	0.451	0.428	0.459	0.415	0.447
14	68660428	T	C	2.595	0.842	0.836	0.844	0.837	0.830	0.819	0.833	0.824	0.823	0.819	0.830	0.824
14	68979835	T	C	2.492	0.262	0.262	0.265	0.260	0.250	0.237	0.243	0.241	0.272	0.234	0.256	0.246
14	91751788	TC	T	2.826	0.695	0.695	0.693	0.692	0.702	0.708	0.702	0.697	0.683	0.714	0.698	0.704
14	91841069	A	G	2.865	0.342	0.351	0.339	0.344	0.361	0.363	0.349	0.351	0.377	0.361	0.354	0.365
14	93070286	C	T	2.570	0.163	0.170	0.177	0.173	0.167	0.164	0.166	0.163	0.161	0.159	0.172	0.167
14	105213978	T	G	2.831	0.457	0.458	0.459	0.458	0.468	0.473	0.471	0.469	0.479	0.465	0.468	0.465
15	46680811	C	A	2.273	0.012	0.011	0.011	0.011	0.009	0.010	0.011	0.010	0.010	0.008	0.009	0.011
15	50694306	A	G	2.609	0.350	0.337	0.345	0.341	0.337	0.337	0.339	0.338	0.319	0.342	0.331	0.329
15	66630569	G	A	2.622	0.653	0.645	0.652	0.643	0.641	0.629	0.644	0.632	0.644	0.641	0.658	0.637
15	67457698	A	G	2.949	0.051	0.050	0.052	0.051	0.055	0.052	0.057	0.055	0.057	0.053	0.058	0.053
15	75750383	T	C	2.610	0.247	0.262	0.249	0.257	0.250	0.260	0.242	0.257	0.243	0.268	0.246	0.258
15	91512267	G	T	2.567	0.133	0.136	0.134	0.135	0.127	0.123	0.128	0.126	0.130	0.125	0.124	0.123
15	100905819	A	C	2.563	0.110	0.107	0.108	0.109	0.104	0.106	0.104	0.107	0.098	0.110	0.105	0.111
16	4008542	CAAAAA	C	2.632	0.834	0.820	0.836	0.822	0.834	0.816	0.833	0.817	0.833	0.811	0.832	0.810
16	4106788	C	A	2.639	0.228	0.264	0.227	0.268	0.222	0.260	0.223	0.261	0.228	0.265	0.221	0.254
16	6963972	C	G	2.818	0.774	0.786	0.773	0.785	0.780	0.790	0.776	0.784	0.784	0.776	0.784	0.793
16	10706580	G	A	2.531	0.063	0.070	0.064	0.068	0.060	0.066	0.063	0.066	0.063	0.067	0.062	0.068
16	23007047	G	T	3.094	0.024	0.022	0.024	0.024	0.025	0.026	0.027	0.025	0.024	0.025	0.024	0.024
16	52538825	C	A	3.070	0.254	0.263	0.246	0.252	0.298	0.308	0.293	0.297	0.304	0.303	0.269	0.292
16	52599188	C	T	3.043	0.240	0.249	0.232	0.236	0.283	0.291	0.278	0.283	0.291	0.287	0.257	0.275
16	53809123	C	T	2.540	0.409	0.416	0.412	0.418	0.390	0.401	0.397	0.407	0.374	0.394	0.402	0.416
16	53861139	C	T	2.629	0.755	0.767	0.763	0.759	0.756	0.757	0.752	0.755	0.766	0.755	0.759	0.759

16	53861592	G	A	2.630	0.346	0.372	0.361	0.363	0.351	0.362	0.349	0.362	0.354	0.358	0.367	0.361
16	54682064	G	A	2.854	0.495	0.490	0.491	0.486	0.502	0.505	0.504	0.496	0.512	0.483	0.503	0.495
16	80648296	A	G	2.967	0.223	0.228	0.225	0.229	0.243	0.248	0.241	0.245	0.237	0.231	0.235	0.240
16	85145977	T	C	2.662	0.469	0.490	0.469	0.484	0.468	0.487	0.466	0.488	0.459	0.480	0.465	0.484
16	87086492	T	C	2.597	0.245	0.256	0.247	0.258	0.232	0.254	0.238	0.252	0.232	0.260	0.243	0.251
17	29168077	G	T	2.572	0.257	0.261	0.264	0.259	0.254	0.256	0.251	0.254	0.255	0.259	0.254	0.259
17	39251123	T	C	2.954	0.058	0.069	0.060	0.067	0.065	0.071	0.063	0.071	0.065	0.069	0.064	0.066
17	40127060	T	C	2.766	0.042	0.060	0.043	0.056	0.044	0.060	0.041	0.057	0.040	0.057	0.044	0.056
17	40485239	G	T	2.572	0.086	0.087	0.087	0.087	0.083	0.080	0.086	0.082	0.072	0.078	0.088	0.084
17	40744470	G	A	3.399	0.010	0.013	0.010	0.013	0.014	0.015	0.012	0.015	0.013	0.012	0.013	0.013
17	43212339	C	CT	2.843	0.204	0.224	0.202	0.226	0.215	0.238	0.210	0.236	0.211	0.217	0.204	0.237
17	44283858	G	A	2.579	0.177	0.190	0.185	0.188	0.180	0.187	0.175	0.187	0.174	0.188	0.186	0.184
17	53209774	A	C	2.519	0.294	0.295	0.302	0.303	0.280	0.285	0.283	0.289	0.315	0.288	0.299	0.295
17	77781725	A	G	2.614	0.505	0.504	0.505	0.506	0.494	0.497	0.492	0.495	0.499	0.506	0.500	0.499
18	11696613	C	T	2.619	0.143	0.138	0.145	0.135	0.144	0.136	0.139	0.136	0.149	0.128	0.139	0.136
18	20634253	C	T	2.610	0.639	0.640	0.637	0.641	0.626	0.632	0.627	0.632	0.630	0.621	0.629	0.638
18	24125857	T	C	2.816	0.418	0.422	0.420	0.422	0.432	0.432	0.429	0.434	0.449	0.437	0.427	0.435
18	24337424	C	G	2.848	0.617	0.622	0.624	0.622	0.638	0.639	0.633	0.632	0.674	0.645	0.638	0.628
18	24518050	AT	A	2.565	0.284	0.277	0.293	0.280	0.282	0.263	0.278	0.265	0.278	0.276	0.278	0.262
18	25407513	C	G	2.831	0.713	0.717	0.707	0.713	0.716	0.715	0.719	0.716	0.713	0.714	0.718	0.712
18	29981526	G	A	2.459	0.049	0.047	0.047	0.048	0.042	0.040	0.044	0.042	0.035	0.035	0.042	0.040
18	42411803	G	C	2.499	0.072	0.074	0.072	0.072	0.069	0.069	0.065	0.067	0.072	0.065	0.073	0.073
18	42888797	T	C	2.579	0.345	0.352	0.351	0.351	0.338	0.338	0.339	0.339	0.336	0.335	0.346	0.344

19	13249921	G	T	3.005	0.034	0.052	0.037	0.052	0.040	0.052	0.039	0.055	0.037	0.059	0.039	0.056
19	17393925	C	A	2.825	0.295	0.298	0.297	0.294	0.306	0.306	0.303	0.298	0.320	0.288	0.301	0.291
19	18569492	C	T	2.536	0.351	0.353	0.351	0.350	0.328	0.333	0.335	0.332	0.337	0.349	0.330	0.330
19	19517054	C	CGGGCG	2.842	0.375	0.355	0.368	0.356	0.372	0.364	0.376	0.362	0.384	0.374	0.379	0.360
19	44283031	T	C	2.898	0.356	0.351	0.352	0.351	0.369	0.372	0.367	0.370	0.366	0.370	0.365	0.358
19	46166073	T	C	2.624	0.619	0.603	0.609	0.606	0.605	0.597	0.608	0.600	0.607	0.592	0.610	0.604
19	55816678	C	T	2.624	0.358	0.362	0.368	0.363	0.360	0.358	0.356	0.356	0.366	0.369	0.353	0.364
20	5948227	G	A	2.942	0.060	0.060	0.062	0.065	0.066	0.067	0.066	0.069	0.065	0.064	0.072	0.066
20	11379842	T	C	2.969	0.946	0.948	0.945	0.948	0.952	0.955	0.949	0.951	0.941	0.947	0.951	0.952
20	41613706	C	G	2.807	0.785	0.794	0.787	0.791	0.791	0.795	0.799	0.794	0.792	0.796	0.799	0.795
20	52296849	G	A	2.843	0.236	0.242	0.239	0.238	0.246	0.251	0.248	0.250	0.245	0.261	0.247	0.249
21	16364756	T	G	2.906	0.172	0.172	0.171	0.174	0.174	0.186	0.176	0.182	0.165	0.194	0.177	0.179
21	16566350	A	G	2.890	0.089	0.090	0.087	0.087	0.091	0.093	0.097	0.092	0.074	0.087	0.088	0.083
21	16574455	C	A	2.539	0.319	0.314	0.318	0.316	0.303	0.302	0.301	0.300	0.307	0.296	0.305	0.310
21	47762932	G	A	3.002	0.038	0.034	0.034	0.036	0.037	0.038	0.039	0.038	0.033	0.033	0.032	0.032
22	19766137	C	T	2.622	0.367	0.385	0.369	0.375	0.371	0.377	0.361	0.377	0.370	0.377	0.374	0.381
22	29121087	A	G	3.326	NA	0.005	NA	0.005	NA	0.006	NA	0.006	NA	0.003	NA	0.005
22	29135543	G	A	2.908	0.091	0.087	0.089	0.088	0.095	0.088	0.098	0.090	0.097	0.090	0.082	0.088
22	29203724	C	T	3.161	0.019	0.021	0.021	0.021	0.028	0.028	0.025	0.023	0.025	0.029	0.026	0.025
22	29551872	A	G	2.322	0.983	0.985	0.979	0.984	0.976	0.981	0.977	0.982	0.979	0.979	0.976	0.981
22	38583315	AAAAG	AAAAGAAAG	2.596	0.272	0.285	0.276	0.277	0.273	0.272	0.269	0.273	0.275	0.265	0.271	0.273
22	39343916	T	A	2.834	0.255	0.254	0.245	0.256	0.253	0.256	0.257	0.258	0.258	0.258	0.246	0.255
22	40904707	CT	C	3.070	0.108	0.109	0.109	0.108	0.123	0.121	0.121	0.120	0.108	0.115	0.120	0.120

22	43433100	C	T	2.565	0.127	0.114	0.123	0.117	0.120	0.110	0.114	0.112	0.107	0.102	0.105	0.111
22	45319953	G	A	2.682	0.438	0.420	0.436	0.419	0.430	0.412	0.432	0.411	0.425	0.423	0.436	0.423
22	46283297	G	A	2.934	0.113	0.112	0.114	0.111	0.118	0.116	0.118	0.116	0.114	0.114	0.118	0.111

Supplementary Table S3. Mean and standard deviation of polygenic risk score in non-breast cancer controls from Europeans and Asians, before exclusions of studies with high missing rates for Gail model risk factors.

Region/ country	n	Mean	Standard deviation
**Region**			
European	96,726	-0.412	0.621
Asian	13,580	0.197	0.556
**European population**			
Australia	4,231	-0.437	0.62
Belarus	344	-0.409	0.676
Belgium	1,887	-0.444	0.601
Canada	2,402	-0.425	0.633
Denmark	5,057	-0.468	0.602
Finland	865	-0.452	0.61
France	1,543	-0.424	0.621
Germany	7,123	-0.432	0.615
Greece	309	-0.292	0.607
Ireland	86	-0.446	0.611
Israel	719	-0.377	0.644
Italy	1,723	-0.34	0.619
Netherlands	2,600	-0.4	0.631
Norway	258	-0.368	0.595
Poland	2,684	-0.424	0.628
Republic of North Macedonia	88	-0.28	0.591
Russia	115	-0.336	0.688
Spain	2,227	-0.386	0.619
Sweden	16,853	-0.447	0.619
UK	20,355	-0.401	0.623
USA	25,257	-0.383	0.621
**Asian population**			
Canada	252	0.291	0.573
China	1,871	0.178	0.544
Hong Kong	531	0.199	0.581
Japan	2,162	0.223	0.548
Korea	3,201	0.275	0.551
Malaysia	1,858	0.092	0.566
Singapore	932	0.152	0.554
Taiwan	641	0.232	0.555
Thailand	1,080	0.114	0.537
USA	1,051	0.212	0.553

Supplementary Table S4. Demographic and risk factors differences between included and excluded individuals.

	European				Asian			
	**Included and excluded, n=196,414**	**Included, n=161,849 (82%)**	**Excluded, n=34,565 (18%)**		**Included and excluded, n=25,440**	**Included, n=18,549 (73%)**	**Excluded, n=6,891 (27%)**	
**Median age (at interview/ diagnosis), years (IQR)**	56 (48 to 64)	57 (49 to 65)	54 (45 to 63)	<0.001	50 (44 to 58)	50 (44 to 58)	50 (44 to 57)	0.062
**Age at menarche, n (%)**								
<12 years	23,742 (12)	22,417 (14)	1,325 (4)	<0.001	1,543 (6)	1,155 (6)	388 (6)	<0.001
12 to 14 years	72,071 (37)	68,265 (42)	3,806 (11)		8,159 (32)	6,684 (36)	1,475 (21)	
≥14 years	50,362 (26)	47,060 (29)	3,302 (10)		10,978 (43)	8,974 (48)	2,004 (29)	
Unknown	50,239 (26)	24,107 (15)	26,132 (76)		4,760 (19)	1,736 (9)	3,024 (44)	
**Age at first full term pregnancy, n (%)**								
Nulliparous	22,988 (12)	20,608 (13)	2,380 (7)	<0.001	2,850 (11)	2,399 (13)	451 (7)	<0.001
<20 years	12,620 (6)	12,359 (8)	261 (1)		736 (3)	707 (4)	29 (0)	
20 to 24 years	47,710 (24)	46,689 (29)	1,021 (3)		4,267 (17)	3,893 (21)	374 (5)	
25 to 29 years	38,960 (20)	38,099 (24)	861 (2)		6,635 (26)	6,159 (33)	476 (7)	
≥30 years	20,284 (10)	19,846 (12)	438 (1)		2,955 (12)	2,859 (15)	96 (1)	
Unknown	53,852 (27)	24,248 (15)	29,604 (86)		7,997 (31)	2,532 (14)	5,465 (79)	
**Number of first-degree family history of breast cancer, n (%)**								
No	106,831 (54)	98,537 (61)	8,294 (24)	<0.001	16,449 (65)	15,389 (83)	1,060 (15)	<0.001
1	19,065 (10)	16,719 (10)	2,346 (7)		1,455 (6)	1,427 (8)	28 (0)	
≥2	3,447 (2)	3,160 (2)	287 (1)		169 (1)	169 (1)	0 (0)	
Unknown	67,071 (34)	43,433 (27)	23,638 (68)		7,367 (29)	1,564 (8)	5,803 (84)	
**Number of breast biopsy, n (%)**								
No	4,207 (2)	4,207 (3)	0 (0)		0 (0)	0 (0)	0 (0)	
1	3,641 (2)	3,641 (2)	0 (0)		0 (0)	0 (0)	0 (0)	
≥2	2,073 (1)	2,073 (1)	0 (0)		0 (0)	0 (0)	0 (0)	
Unknown	186,493 (95)	151,928 (94)	34,565 (100)		25,440 (100)	18,549 (100)	6,891 (100)	
**Atypical hyperplasia, n (%)**								
No	4,207 (2)	4,207 (3)	0 (0)		0 (0)	0 (0)	0 (0)	
Yes	62 (0)	62 (0)	0 (0)		0 (0)	0 (0)	0 (0)	
Unknown	192,145 (98)	157,580 (97)	34,565 (100)		25,440 (100)	18,549 (100)	6,891 (100)	
**Median 5-year absolute risk by the Gail model (IQR)**	1.154 (0.833 to 1.597)	1.248 (0.910 to 1.699)	0.842 (0.575 to 1.120)	<0.001	0.513 (0.382 to 0.730)	0.609 (0.447 to 0.793)	0.413 (0.350 to 0.477)	<0.001
**Protein truncating variants (9 Genes)**								
No	61,433 (31)	53,803 (33)	7,630 (22)	0.538	3,457 (14)	3,457 (19)	0 (0)	
Yes	2,937 (1)	2,584 (2)	353 (1)		160 (1)	160 (1)	0 (0)	
Unknown	132,044 (67)	105,462 (65)	26,582 (77)		21,823 (86)	14,932 (81)	6,891 (100)	
**Polygenic risk score (PRS)**	-0.245 (-0.676 to 0.189)	-0.247 (-0.677 to 0.186)	-0.237 (-0.673 to 0.201)	0.002	0.287 (-0.087 to 0.676)	0.279 (-0.097 to 0.675)	0.308 (-0.056 to 0.678)	0.002
**Median 5-year absolute risk by PRS (IQR)**	0.821 (0.525 to 1.264)	0.829 (0.536 to 1.273)	0.777 (0.479 to 1.223)	<0.001	0.691 (0.430 to 1.069)	0.681 (0.419 to 1.063)	0.716 (0.463 to 1.088)	<0.001

Supplementary Table S5. Demographic and risk factors differences between included (Yes) and excluded (No) individuals of European-ancestry aged 30 to 49 years.

	Control			Invasive			DCIS		
	Yes, n=17,082	No, n=5,264	P	Yes, n=23,224	No, n=6,866		Yes, n=2,342	No, n=484	P
**Median age (at interview/ diagnosis), years (IQR)**	44 (41 to 47)	42 (37 to 46)	<0.001	44 (40 to 47)	42 (38 to 46)	<0.001	45 (42 to 47)	44 (40 to 47)	0.003
**Age at menarche, n (%)**									
<12	2,531 (15)	124 (2)	0.636	3,150 (14)	266 (4)	<0.001	404 (17)	68 (14)	<0.001
12 to 14	7,731 (45)	358 (7)		9,603 (41)	756 (11)		1,105 (47)	94 (19)	
≥14	4,486 (26)	225 (4)		5,328 (23)	526 (8)		631 (27)	57 (12)	
Unknown	2,334 (14)	4,557 (87)		5,143 (22)	5,318 (77)		202 (9)	265 (55)	
**Age at first full term pregnancy, n (%)**									
Nulliparous	2,588 (15)	583 (11)	<0.001	3,737 (16)	301 (4)	<0.001	452 (19)	43 (9)	<0.001
<20	1,031 (6)	21 (0)		1,322 (6)	29 (0)		122 (5)	0 (0)	
20 to 24	3,866 (23)	87 (2)		4,285 (18)	84 (1)		491 (21)	3 (1)	
25 to 29	4,432 (26)	113 (2)		5,008 (22)	137 (2)		590 (25)	5 (1)	
≥30	3,083 (18)	62 (1)		3,902 (17)	129 (2)		476 (20)	5 (1)	
Unknown	2,082 (12)	4,398 (84)		4,970 (21)	6,186 (90)		211 (9)	428 (88)	
**Number of first degree family history of breast cancer, n (%)**									
No	11,340 (66)	1,357 (26)	0.078	14,098 (61)	2,064 (30)	<0.001	905 (39)	61 (13)	0.002
1	1,295 (8)	135 (3)		3,370 (15)	770 (11)		348 (15)	47 (10)	
≥2	137 (1)	9 (0)		537 (2)	86 (1)		80 (3)	10 (2)	
Unknown	4,310 (25)	3,763 (71)		5,219 (22)	3,946 (57)		1,009 (43)	366 (76)	
**Number of breast biopsy, n (%)**									
No	301 (2)	0 (0)		1,310 (6)	0 (0)		43 (2)	0 (0)	
1	74 (0)	0 (0)		784 (3)	0 (0)		60 (3)	0 (0)	
≥2	23 (0)	0 (0)		532 (2)	0 (0)		42 (2)	0 (0)	
Unknown	16,684 (98)	5,264 (100)		20,598 (89)	6,866 (100)		2,197 (94)	484 (100)	
**Atypical hyperplasia, n (%)**									
No	301 (2)	0 (0)		1,310 (6)	0 (0)		43 (2)	0 (0)	
Yes	1 (0)	0 (0)		13 (0)	0 (0)		1 (0)	0 (0)	
Unknown	16,780 (98)	5,264 (100)		21,901 (94)	6,866 (100)		2,298 (98)	484 (100)	
**Median 5-year absolute risk by the Gail model (IQR)**	0.746 (0.555 to 0.948)	0.467 (0.260 to 0.587)	<0.001	0.739 (0.522 to 1.035)	0.502 (0.324 to 0.613)	<0.001	0.860 (0.652 to 1.126)	0.562 (0.433 to 0.652)	<0.001
**Protein truncating variants (9 Genes)**									
No	4,687 (27)	1,029 (20)	<0.001	7,748 (33)	1,201 (17)	0.687	406 (17)	40 (8)	0.055
Yes	173 (1)	13 (0)		808 (3)	131 (2)		21 (1)	6 (1)	
Unknown	12,222 (72)	4,222 (80)		14,668 (63)	5,534 (81)		1,915 (82)	438 (90)	
**Polygenic risk score (PRS)**	-0.426 (-0.842 to -0.010)	-0.436 (-0.841 to -0.033)	0.437	-0.058 (-0.484 to 0.367)	-0.036 (-0.459 to 0.371)	0.066	-0.077 (-0.484 to 0.328)	-0.053 (-0.485 to 0.376)	0.335
**Median 5-year absolute risk by PRS (IQR)**	0.499 (0.314 to 0.769)	0.416 (0.238 to 0.678)	<0.001	0.683 (0.415 to 1.068)	0.620 (0.368 to 1.000)	<0.001	0.718 (0.477 to 1.068)	0.712 (0.451 to 1.063)	0.207

Supplementary Table S6. Demographic and risk factors differences between included (Yes) and excluded (No) individuals of European-ancestry aged 50 to 80 years.

	Control			Invasive			DCIS		
	Yes, n=51,458	No, n=9,570	P	Yes, n=60,461	No, n=11,318		Yes, n=7,282	No, n=1,063	P
**Median age (at interview/ diagnosis), years (IQR)**	61 (55 to 66)	61 (55 to 68)	<0.001	61 (56 to 67)	60 (54 to 67)	<0.001	59 (54 to 65)	57 (52 to 63)	<0.001
**Age at menarche, n (%)**									
<12	7,294 (14)	300 (3)	<0.001	7,709 (13)	437 (4)	<0.001	1,329 (18)	130 (12)	0.439
12 to 14	22,512 (44)	795 (8)		23,873 (39)	1,505 (13)		3,441 (47)	298 (28)	
≥14	16,647 (32)	787 (8)		17,841 (30)	1,527 (13)		2,127 (29)	180 (17)	
Unknown	5,005 (10)	7,688 (80)		11,038 (18)	7,849 (69)		385 (5)	455 (43)	
**Age at first full term pregnancy, n (%)**									
Nulliparous	5,731 (11)	805 (8)	<0.001	7,032 (12)	526 (5)	<0.001	1,068 (15)	122 (11)	<0.001
<20	4,396 (9)	57 (1)		4,816 (8)	152 (1)		672 (9)	2 (0)	
20 to 24	17,997 (35)	268 (3)		17,642 (29)	557 (5)		2,408 (33)	22 (2)	
25 to 29	13,111 (25)	195 (2)		13,166 (22)	387 (3)		1,792 (25)	24 (2)	
≥30	5,404 (11)	67 (1)		6,071 (10)	170 (2)		910 (12)	5 (0)	
Unknown	4,819 (9)	8,178 (85)		11,734 (19)	9,526 (84)		432 (6)	888 (84)	
**Number of first degree family history of breast cancer, n (%)**									
No	34,289 (67)	1,223 (13)	<0.001	34,805 (58)	3,457 (31)	<0.001	3,100 (43)	132 (12)	0.462
1	4,053 (8)	98 (1)		6,886 (11)	1,256 (11)		767 (11)	40 (4)	
≥2	654 (1)	18 (0)		1,527 (3)	152 (1)		225 (3)	12 (1)	
Unknown	12,462 (24)	8,231 (86)		17,243 (29)	6,453 (57)		3,190 (44)	879 (83)	
**Number of breast biopsy, n (%)**									
No	629 (1)	0 (0)		1,871 (3)	0 (0)		53 (1)	0 (0)	
1	203 (0)	0 (0)		2,364 (4)	0 (0)		156 (2)	0 (0)	
≥2	80 (0)	0 (0)		1,290 (2)	0 (0)		106 (1)	0 (0)	
Unknown	50,546 (98)	9,570 (100)		54,936 (91)	11,318 (100)		6,967 (96)	1,063 (100)	
**Atypical hyperplasia, n (%)**									
No	629 (1)	0 (0)		1,871 (3)	0 (0)		53 (1)	0 (0)	
Yes	4 (0)	0 (0)		36 (0)	0 (0)		7 (0)	0 (0)	
Unknown	50,825 (99)	9,570 (100)		58,554 (97)	11,318 (100)		7,222 (99)	1,063 (100)	
**Median 5-year absolute risk by the Gail model (IQR)**	1.404 (1.128 to 1.749)	1.032 (0.808 to 1.147)	<0.001	1.428 (1.109 to 1.883)	1.060 (0.842 to 1.161)	<0.001	1.411 (1.149 to 1.811)	0.998 (0.773 to 1.145)	<0.001
**Protein truncating variants (9 Genes)**									
No	19,528 (38)	1,561 (16)	0.381	20,178 (33)	3,581 (32)	0.071	1,256 (17)	218 (21)	0.425
Yes	410 (1)	27 (0)		1,119 (2)	170 (2)		53 (1)	6 (1)	
Unknown	31,520 (61)	7,982 (83)		39,164 (65)	7,567 (67)		5,973 (82)	839 (79)	
**Polygenic risk score (PRS)**	-0.459 (-0.870 to -0.046)	-0.459 (-0.868 to -0.045)	0.645	-0.107 (-0.517 to 0.309)	-0.083 (-0.496 to 0.346)	<0.001	-0.170 (-0.572 to 0.248)	-0.177 (-0.607 to 0.238)	0.488
**Median 5-year absolute risk by PRS (IQR)**	0.763 (0.519 to 1.130)	0.768 (0.523 to 1.148)	0.142	1.055 (0.718 to 1.568)	1.069 (0.727 to 1.585)	0.05	0.971 (0.670 to 1.448)	0.945 (0.646 to 1.419)	0.132

Supplementary Table S7. Demographic and risk factors differences between included (Yes) and excluded (No) individuals of Asian-ancestry aged 30 to 49 years. No DCIS were excluded.

	Control			Invasive			DCIS
	Yes, n=3,822	No, n=1,831	P	Yes, n=4,634	No, n=1,539	P	Yes, n=500
**Median age (at interview/ diagnosis), years (IQR)**	44 (40 to 47)	44 (41 to 47)	<0.001	43 (38 to 47)	44 (40 to 46)	<0.001	43 (38 to 47)
**Age at menarche, n (%)**							
<12	323 (8)	101 (6)	0.225	377 (8)	60 (4)	<0.001	38 (8)
12 to 14	1,523 (40)	504 (28)		1,774 (38)	206 (13)		185 (37)
≥14	1,747 (46)	639 (35)		1,897 (41)	319 (21)		220 (44)
Unknown	229 (6)	587 (32)		586 (13)	954 (62)		57 (11)
**Age at first full term pregnancy, n (%)**							
Nulliparous	629 (16)	123 (7)	<0.001	791 (17)	105 (7)	<0.001	108 (22)
<20	134 (4)	8 (0)		127 (3)	0 (0)		13 (3)
20 to 24	684 (18)	160 (9)		707 (15)	1 (0)		40 (8)
25 to 29	1,423 (37)	302 (16)		1,578 (34)	0 (0)		142 (28)
≥30	625 (16)	65 (4)		838 (18)	0 (0)		81 (16)
Unknown	327 (9)	1,173 (64)		593 (13)	1,433 (93)		116 (23)
**Number of first degree family history of breast cancer, n (%)**							
No	3,389 (89)	303 (17)	0.249	3,858 (83)	278 (18)	<0.001	360 (72)
1	207 (5)	16 (1)		434 (9)	0 (0)		63 (13)
≥2	28 (1)	0 (0)		44 (1)	0 (0)		6 (1)
Unknown	198 (5)	1,512 (83)		298 (6)	1,261 (82)		71 (14)
**Number of breast biopsy, n (%)**							
No	0 (0)	0 (0)		0 (0)	0 (0)		0 (0)
1	0 (0)	0 (0)		0 (0)	0 (0)		0 (0)
≥2	0 (0)	0 (0)		0 (0)	0 (0)		0 (0)
Unknown	3,822 (100)	1,831 (100)		4,634 (100)	1,539 (100)		500 (100)
**Atypical hyperplasia, n (%)**							
No	0 (0)	0 (0)		0 (0)	0 (0)		0 (0)
Yes	0 (0)	0 (0)		0 (0)	0 (0)		0 (0)
Unknown	3,822 (100)	1,831 (100)		4,634 (100)	1,539 (100)		500 (100)
**Median 5-year absolute risk by the Gail model (IQR)**	0.501 (0.344 to 0.652)	0.351 (0.296 to 0.408)	<0.001	0.466 (0.294 to 0.654)	0.322 (0.217 to 0.351)	<0.001	0.446 (0.269 to 0.647)
**Protein truncating variants (9 Genes)**							
No	751 (20)	0 (0)		1,305 (28)	0 (0)		184 (37)
Yes	18 (0)	0 (0)		95 (2)	0 (0)		5 (1)
Unknown	3,053 (80)	1,831 (100)		3,234 (70)	1,539 (100)		311 (62)
**Polygenic risk score (PRS)**	0.181 (-0.169 to 0.544)	0.234 (-0.127 to 0.595)	0.003	0.393 (-0.002 to 0.785)	0.452 (0.101 to 0.806)	<0.001	0.414 (-0.008 to 0.799)
**Median 5-year absolute risk by PRS (IQR)**	0.489 (0.295 to 0.743)	0.541 (0.362 to 0.818)	<0.001	0.547 (0.311 to 0.906)	0.638 (0.409 to 0.976)	<0.001	0.532 (0.335 to 0.899)

Supplementary Table S8. Demographic and risk factors differences between included (Yes) and excluded (No) individuals of Asian-ancestry aged 50 to 80 years.

	Control			Invasive			DCIS		
	Yes, n=4,525	No, n=2,147	P	Yes, n=4,588	No, n=1,369	P	Yes, n=480	No, n=5	P
**Median age (at interview/ diagnosis), years (IQR)**	57 (53 to 63)	57 (53 to 63)	0.305	57 (53 to 64)	57 (53 to 63)	0.565	56 (52 to 62)	57 (52 to 72)	0.718
**Age at menarche, n (%)**									
<12	185 (4)	152 (7)	<0.001	209 (5)	75 (5)	<0.001	23 (5)	0 (0)	0.231
12 to 14	1,557 (34)	496 (23)		1,504 (33)	266 (19)		141 (29)	3 (60)	
≥14	2,574 (57)	680 (32)		2,289 (50)	365 (27)		247 (51)	1 (20)	
Unknown	209 (5)	819 (38)		586 (13)	663 (48)		69 (14)	1 (20)	
**Age at first full term pregnancy, n (%)**									
Nulliparous	376 (8)	115 (5)	<0.001	445 (10)	107 (8)	<0.001	50 (10)	1 (20)	0.157
<20	182 (4)	21 (1)		235 (5)	0 (0)		16 (3)	0 (0)	
20 to 24	1,264 (28)	213 (10)		1,103 (24)	0 (0)		95 (20)	0 (0)	
25 to 29	1,413 (31)	174 (8)		1,452 (32)	0 (0)		151 (31)	0 (0)	
≥30	524 (12)	31 (1)		716 (16)	0 (0)		75 (16)	0 (0)	
Unknown	766 (17)	1,593 (74)		637 (14)	1,262 (92)		93 (19)	4 (80)	
**Number of first degree family history of breast cancer, n (%)**									
No	3,792 (84)	285 (13)	0.131	3,642 (79)	194 (14)	<0.001	348 (72)	0 (0)	
1	230 (5)	12 (1)		442 (10)	0 (0)		51 (11)	0 (0)	
≥2	35 (1)	0 (0)		49 (1)	0 (0)		7 (1)	0 (0)	
Unknown	468 (10)	1,850 (86)		455 (10)	1,175 (86)		74 (15)	5 (100)	
**Number of breast biopsy, n (%)**									
No	0 (0)	0 (0)		0 (0)	0 (0)		0 (0)	0 (0)	
1	0 (0)	0 (0)		0 (0)	0 (0)		0 (0)	0 (0)	
≥2	0 (0)	0 (0)		0 (0)	0 (0)		0 (0)	0 (0)	
Unknown	4,525 (100)	2,147 (100)		4,588 (100)	1,369 (100)		480 (100)	5 (100)	
**Atypical hyperplasia, n (%)**									
No	0 (0)	0 (0)		0 (0)	0 (0)		0 (0)	0 (0)	
Yes	0 (0)	0 (0)		0 (0)	0 (0)		0 (0)	0 (0)	
Unknown	4,525 (100)	2,147 (100)		4,588 (100)	1,369 (100)		480 (100)	5 (100)	
**Median 5-year absolute risk by the Gail model (IQR)**	0.678 (0.549 to 0.828)	0.465 (0.438 to 0.537)	<0.001	0.759 (0.589 to 0.882)	0.450 (0.424 to 0.477)	<0.001	0.759 (0.565 to 0.938)	0.462 (0.411 to 0.494)	0.041
**Protein truncating variants (9 Genes)**									
No	340 (8)	0 (0)		744 (16)	0 (0)		133 (28)	0 (0)	
Yes	6 (0)	0 (0)		34 (1)	0 (0)		2 (0)	0 (0)	
Unknown	4,179 (92)	2,147 (100)		3,810 (83)	1,369 (100)		345 (72)	5 (100)	
**Polygenic risk score (PRS)**	0.152 (-0.215 to 0.523)	0.214 (-0.164 to 0.551)	0.002	0.357 (-0.018 to 0.737)	0.399 (0.057 to 0.802)	<0.001	0.484 (0.116 to 0.842)	0.572 (0.421 to 0.731)	0.89
**Median 5-year absolute risk by PRS (IQR)**	0.748 (0.509 to 1.113)	0.788 (0.529 to 1.143)	0.004	0.917 (0.623 to 1.374)	0.974 (0.659 to 1.471)	<0.001	1.057 (0.720 to 1.559)	0.883 (0.876 to 1.476)	0.835

Supplementary Table S9. Sensitivity analysis of the discriminatory ability of the Gail model, assuming that missing values correspond to the highest risk category rather than the reference category. Five-year absolute risk was estimated using the R package BCRA and applied to predict the risk of invasive breast cancer. AUC: Area under the receiver operator curve, CI: Confidence interval, AgeMen: Age at menarche, Age1st: Age at first live birth, N_Rels: Number of first-degree relatives with breast cancer, N_Biop: Number of prior biopsy. *Original model: the model using the conventional approach of assigning missing values to the lowest risk group

Ancestry	Age group, years	Variables where missing values were assumed to correspond to the highest risk category	AUC (95%CI)
European	<50	AgeMen	0.484 (0.479 to 0.490)
	<50	Age1st	0.550 (0.544 to 0.555)
	<50	N_Rels	0.585 (0.579 to 0.590)
	<50	N_Biop	0.538 (0.532 to 0.543)
	<50	All	0.627 (0.621 to 0.632)
	<50	**Original model*	*0.493 (0.487 to 0.499)*
	≥50	AgeMen	0.529 (0.526 to 0.533)
	≥50	Age1st	0.578 (0.574 to 0.581)
	≥50	N_Rels	0.534 (0.530 to 0.537)
	≥50	N_Biop	0.506 (0.502 to 0.509)
	≥50	All	0.561 (0.557 to 0.564)
	≥50	**Original model*	*0.517 (0.514 to 0.520)*
Asian	<50	AgeMen	0.518 (0.506 to 0.530)
	<50	Age1st	0.499 (0.486 to 0.511)
	<50	N_Rels	0.514 (0.502 to 0.526)
	<50	N_Biop	0.523 (0.511 to 0.535)
	<50	All	0.507 (0.494 to 0.519)
	<50	**Original model*	*0.523 (0.511 to 0.535)*
	≥50	AgeMen	0.565 (0.553 to 0.576)
	≥50	Age1st	0.533 (0.522 to 0.545)
	≥50	N_Rels	0.558 (0.546 to 0.570)
	≥50	N_Biop	0.554 (0.543 to 0.566)
	≥50	All	0.531 (0.520 to 0.543)
	≥50	**Original model*	*0.554 (0.543 to 0.566)*

Supplementary Table S10. Sensitivity analysis of the discriminatory ability of the Gail model, assuming that missing values correspond to the highest risk category rather than the reference category. Five-year absolute risk was estimated using the R package BCRA and applied to predict the risk of DCIS. AUC: Area under the receiver operator curve, CI: Confidence interval, AgeMen: Age at menarche, Age1st: Age at first live birth, N_Rels: Number of first-degree relatives with breast cancer, N_Biop: Number of prior biopsy. *Original model: the model using the conventional approach of assigning missing values to the lowest risk group

Ancestry	Age group, years	Variables where missing values were assumed to correspond to the highest risk category	AUC (95%CI)
European	<50	AgeMen	0.607 (0.594 to 0.619)
	<50	Age1st	0.596 (0.584 to 0.608)
	<50	N_Rels	0.548 (0.536 to 0.561)
	<50	N_Biop	0.582 (0.570 to 0.595)
	<50	All	0.521 (0.509 to 0.533)
	<50	**Original model*	*0.610 (0.597 to 0.622)*
	≥50	AgeMen	0.513 (0.506 to 0.521)
	≥50	Age1st	0.503 (0.496 to 0.511)
	≥50	N_Rels	0.528 (0.522 to 0.535)
	≥50	N_Biop	0.489 (0.482 to 0.496)
	≥50	All	0.525 (0.518 to 0.531)
	≥50	**Original model*	*0.519 (0.512 to 0.526)*
Asian	<50	AgeMen	0.531 (0.503 to 0.560)
	<50	Age1st	0.538 (0.509 to 0.567)
	<50	N_Rels	0.544 (0.515 to 0.572)
	<50	N_Biop	0.533 (0.505 to 0.562)
	<50	All	0.589 (0.560 to 0.619)
	<50	**Original model*	*0.533 (0.505 to 0.562)*
	≥50	AgeMen	0.553 (0.524 to 0.582)
	≥50	Age1st	0.570 (0.543 to 0.597)
	≥50	N_Rels	0.624 (0.599 to 0.649)
	≥50	N_Biop	0.542 (0.513 to 0.572)
	≥50	All	0.599 (0.571 to 0.627)
	≥50	**Original model*	*0.542 (0.513 to 0.572)*

Supplementary Table S11. Association between five-year absolute breast cancer risk (modeled as a continuous variable, estimated using the polygenic risk score (PRS) versus the Gail model) and the development of invasive breast cancer or ductal carcinoma in situ (DCIS), stratified by ancestry and age groups, using only population-based controls. OR: odds ratios; CI: confidence
intervals; AUC: area under the curve. ORs and AUCs are estimated separately in age-stratified logistic regression models (<50y and ≥50y). P-values for interaction (risk score × Age) were obtained from separate logistic regression models that included the risk score, age (treated as a continuous variable), and their interaction term; the interaction test assesses statistical heterogeneity of the risk-score effect by age.

	All ages		<50y		≥50y	
	**OR (95% CI)**	**AUC (95% CI)**	**OR (95% CI)**	**AUC (95% CI)**	**OR (95% CI)**	**AUC (95% CI)**
**Invasive**						
European, n=126,587						
PRS	1.98 (1.94 to 2.02)	0.633 (0.629 to 0.636)	2.45 (2.32 to 2.59)	0.618 (0.611 to 0.624)	2.12 (2.07 to 2.17)	0.654 (0.650 to 0.657)
P interaction (PRS X Age)	<0.001					
Gail	1.01 (0.99 to 1.02)	0.514 (0.510 to 0.517)	1.09 (1.05 to 1.14)	0.518 (0.512 to 0.525)	1.05 (1.04 to 1.07)	0.499 (0.495 to 0.503)
P interaction (Gail X Age)	0.111					
PRS and Gail combined		0.635 (0.632 to 0.638)		0.621 (0.615 to 0.628)		0.654 (0.650 to 0.657)
PRS	2.03 (1.99 to 2.07)		2.56 (2.42 to 2.72)		2.12 (2.07 to 2.17)	
Gail	0.91 (0.90 to 0.93)		0.88 (0.84 to 0.92)		1.01 (1.00 to 1.03)	
Asian, n=12,500						
PRS	1.40 (1.31 to 1.50)	0.554 (0.542 to 0.565)	1.42 (1.24 to 1.62)	0.536 (0.520 to 0.553)	1.69 (1.54 to 1.86)	0.601 (0.586 to 0.616)
P interaction (PRS X Age)	0.033					
Gail	1.68 (1.48 to 1.90)	0.538 (0.527 to 0.548)	1.06 (0.86 to 1.30)	0.521 (0.505 to 0.538)	4.70 (3.77 to 5.86)	0.629 (0.615 to 0.644)
P interaction (Gail X Age)	<0.001					
PRS and Gail combined		0.565 (0.554 to 0.575)		0.542 (0.525 to 0.558)		0.656 (0.641 to 0.670)
PRS	1.32 (1.23 to 1.42)		1.47 (1.28 to 1.69)		1.64 (1.49 to 1.80)	
Gail	1.45 (1.28 to 1.65)		0.86 (0.69 to 1.06)		4.50 (3.61 to 5.62)	
**DCIS**						
European, n=52,526						
PRS	1.65 (1.60 to 1.70)	0.623 (0.617 to 0.629)	2.61 (2.39 to 2.85)	0.653 (0.640 to 0.665)	1.59 (1.54 to 1.64)	0.620 (0.613 to 0.627)
P interaction (PRS X Age)	<0.001					
Gail	1.09 (1.06 to 1.12)	0.514 (0.508 to 0.520)	1.73 (1.59 to 1.89)	0.580 (0.567 to 0.593)	1.06 (1.03 to 1.09)	0.499 (0.492 to 0.507)
P interaction (Gail X Age)	<0.001					
PRS and Gail combined		0.623 (0.616 to 0.629)		0.656 (0.644 to 0.668)		0.620 (0.613 to 0.627)
PRS	1.65 (1.60 to 1.70)		2.42 (2.21 to 2.65)		1.59 (1.53 to 1.64)	
Gail	1.02 (0.99 to 1.04)		1.45 (1.32 to 1.58)		1.02 (0.99 to 1.06)	
Asian, n=4,258						
PRS	1.62 (1.45 to 1.80)	0.576 (0.555 to 0.598)	1.50 (1.22 to 1.84)	0.541 (0.510 to 0.571)	2.01 (1.74 to 2.31)	0.655 (0.628 to 0.683)
P interaction (PRS X Age)	0.021					
Gail	1.85 (1.49 to 2.31)	0.522 (0.499 to 0.544)	1.14 (0.78 to 1.65)	0.532 (0.501 to 0.563)	3.78 (2.80 to 5.10)	0.610 (0.580 to 0.640)
P interaction (Gail X Age)	<0.001					
PRS and Gail combined		0.584 (0.561 to 0.606)		0.542 (0.511 to 0.572)		0.695 (0.669 to 0.722)
PRS	1.55 (1.39 to 1.73)		1.52 (1.23 to 1.87)		1.98 (1.72 to 2.29)	
Gail	1.57 (1.25 to 1.97)		0.92 (0.62 to 1.37)		3.67 (2.70 to 4.99)	

Supplementary Table S12. Distribution of Venn diagram segments across ancestry groups, age groups, and risk thresholds.

Group	Status	Threshold	PRS only	PRS + Gail	Gail only	PRS + FH	All	Gail + FH	FH only	All PRS	All Gail	All FH	AUC_{Gail}	AUC_{PRS}	n
Asian, <50y	DCIS	2.5	6	0	0	3	0	0	78	9	0	81	0.500	0.507	500
Asian, ≥50y	DCIS	2.5	30	0	0	7	0	2	63	37	2	72	0.502	0.526	480
European, <50y	DCIS	2.5	41	0	6	12	3	52	432	56	61	499	0.510	0.509	2342
European, ≥50y	DCIS	2.5	308	2	51	53	68	657	648	431	778	1426	0.520	0.516	7282
Asian, <50y	Invasive	2.5	34	0	0	7	0	0	508	41	0	515	0.500	0.502	4634
Asian, ≥50y	Invasive	2.5	207	0	0	38	0	12	509	245	12	559	0.501	0.514	4588
European, <50y	Invasive	2.5	379	1	79	108	23	458	3696	511	561	4285	0.509	0.508	23224
European, ≥50y	Invasive	2.5	3645	56	619	448	628	5456	4439	4777	6759	10971	0.522	0.525	60461
Asian, <50y	Control	2.5	18	0	0	1	0	0	239	19	0	240	NA	NA	3822
Asian, ≥50y	Control	2.5	102	0	0	9	1	4	270	112	5	284	NA	NA	4525
European, <50y	Control	2.5	71	0	4	14	3	99	1729	88	106	1845	NA	NA	17082
European, ≥50y	Control	2.5	1165	7	185	108	166	3090	3374	1446	3448	6738	NA	NA	51458
Asian, <50y	DCIS	2.4	6	0	0	3	0	0	78	9	0	81	0.500	0.506	500
Asian, ≥50y	DCIS	2.4	37	0	0	7	0	2	63	44	2	72	0.501	0.531	480
European, <50y	DCIS	2.4	44	0	10	13	5	59	422	62	74	499	0.512	0.510	2342
European, ≥50y	DCIS	2.4	349	4	80	56	78	686	606	487	848	1426	0.520	0.517	7282
Asian, <50y	Invasive	2.4	39	0	0	9	0	0	506	48	0	515	0.500	0.502	4634
Asian, ≥50y	Invasive	2.4	239	0	0	38	4	28	489	281	32	559	0.502	0.516	4588
European, <50y	Invasive	2.4	429	1	107	123	31	494	3637	584	633	4285	0.510	0.510	23224
European, ≥50y	Invasive	2.4	4021	97	936	472	741	5680	4078	5331	7454	10971	0.524	0.528	60461
Asian, <50y	Control	2.4	20	0	0	1	0	0	239	21	0	240	NA	NA	3822
Asian, ≥50y	Control	2.4	120	0	0	8	2	10	264	130	12	284	NA	NA	4525
European, <50y	Control	2.4	79	0	7	17	4	105	1719	100	116	1845	NA	NA	17082
European, ≥50y	Control	2.4	1318	21	403	122	191	3286	3139	1652	3901	6738	NA	NA	51458
Asian, <50y	DCIS	2.3	6	0	0	3	0	0	78	9	0	81	0.500	0.505	500
Asian, ≥50y	DCIS	2.3	42	0	0	7	0	3	62	49	3	72	0.501	0.535	480
European, <50y	DCIS	2.3	48	0	10	13	5	62	419	66	77	499	0.513	0.510	2342
European, ≥50y	DCIS	2.3	404	6	127	60	94	719	553	564	946	1426	0.521	0.520	7282
Asian, <50y	Invasive	2.3	56	0	0	10	0	0	505	66	0	515	0.500	0.504	4634

Asian, ≥50y	Invasive	2.3	264	0	0	41	5	44	469	310	49	559	0.503	0.518	4588
European, <50y	Invasive	2.3	499	4	119	135	36	524	3590	674	683	4285	0.511	0.511	23224
European, ≥50y	Invasive	2.3	4465	157	1408	471	855	5995	3650	5948	8415	10971	0.525	0.531	60461
Asian, <50y	Control	2.3	26	0	0	1	0	0	239	27	0	240	NA	NA	3822
Asian, ≥50y	Control	2.3	135	0	0	9	2	16	257	146	18	284	NA	NA	4525
European, <50y	Control	2.3	100	0	7	20	4	111	1710	124	122	1845	NA	NA	17082
European, ≥50y	Control	2.3	1492	40	799	135	223	3507	2873	1890	4569	6738	NA	NA	51458
Asian, <50y	DCIS	2.2	9	0	0	3	0	0	78	12	0	81	0.500	0.508	500
Asian, ≥50y	DCIS	2.2	47	0	0	8	0	5	59	55	5	72	0.502	0.538	480
European, <50y	DCIS	2.2	58	0	14	15	7	64	413	80	85	499	0.514	0.512	2342
European, ≥50y	DCIS	2.2	434	12	164	65	107	744	510	618	1027	1426	0.521	0.521	7282
Asian, <50y	Invasive	2.2	70	0	0	12	0	0	503	82	0	515	0.500	0.505	4634
Asian, ≥50y	Invasive	2.2	310	0	0	46	6	56	451	362	62	559	0.504	0.520	4588
European, <50y	Invasive	2.2	589	7	150	153	42	562	3528	791	761	4285	0.513	0.512	23224
European, ≥50y	Invasive	2.2	4978	257	1879	485	988	6165	3333	6708	9289	10971	0.527	0.534	60461
Asian, <50y	Control	2.2	29	0	0	1	0	0	239	30	0	240	NA	NA	3822
Asian, ≥50y	Control	2.2	157	0	0	12	3	23	246	172	26	284	NA	NA	4525
European, <50y	Control	2.2	129	0	9	25	5	115	1700	159	129	1845	NA	NA	17082
European, ≥50y	Control	2.2	1709	61	1119	148	268	3674	2648	2186	5122	6738	NA	NA	51458
Asian, <50y	DCIS	2.1	10	0	0	4	0	0	77	14	0	81	0.500	0.509	500
Asian, ≥50y	DCIS	2.1	57	0	0	9	0	6	57	66	6	72	0.503	0.547	480
European, <50y	DCIS	2.1	67	0	15	15	8	70	406	90	93	499	0.516	0.514	2342
European, ≥50y	DCIS	2.1	489	32	256	67	127	778	454	715	1193	1426	0.521	0.525	7282
Asian, <50y	Invasive	2.1	86	0	0	14	0	0	501	100	0	515	0.500	0.506	4634
Asian, ≥50y	Invasive	2.1	363	0	0	49	9	70	431	421	79	559	0.505	0.524	4588
European, <50y	Invasive	2.1	693	11	158	180	49	571	3485	933	789	4285	0.513	0.515	23224
European, ≥50y	Invasive	2.1	5487	429	2822	486	1157	6368	2960	7559	10776	10971	0.528	0.538	60461
Asian, <50y	Control	2.1	34	0	0	1	0	0	239	35	0	240	NA	NA	3822
Asian, ≥50y	Control	2.1	183	0	0	12	3	30	239	198	33	284	NA	NA	4525
European, <50y	Control	2.1	153	0	11	28	6	120	1691	187	137	1845	NA	NA	17082
European, ≥50y	Control	2.1	1922	125	1965	148	332	3834	2424	2527	6256	6738	NA	NA	51458
Asian, <50y	DCIS	2	12	0	0	5	0	0	76	17	0	81	0.500	0.512	500
Asian, ≥50y	DCIS	2	60	0	0	11	0	6	55	71	6	72	0.501	0.549	480

European, <50y	DCIS	2	86	0	20	20	9	78	392	115	107	499	0.518	0.518	2342
European, ≥50y	DCIS	2	562	46	308	58	158	793	417	824	1305	1426	0.519	0.528	7282
Asian, <50y	Invasive	2	109	0	0	18	0	4	493	127	4	515	0.500	0.508	4634
Asian, ≥50y	Invasive	2	418	0	0	57	9	89	404	484	98	559	0.506	0.527	4588
European, <50y	Invasive	2	822	16	190	204	67	603	3411	1109	876	4285	0.514	0.517	23224
European, ≥50y	Invasive	2	6109	606	3429	481	1356	6555	2579	8552	11946	10971	0.528	0.542	60461
Asian, <50y	Control	2	41	0	0	1	0	1	238	42	1	240	NA	NA	3822
Asian, ≥50y	Control	2	215	0	0	12	3	43	226	230	46	284	NA	NA	4525
European, <50y	Control	2	186	0	12	32	9	133	1671	227	154	1845	NA	NA	17082
European, ≥50y	Control	2	2166	194	2634	158	387	4029	2164	2905	7244	6738	NA	NA	51458
Asian, <50y	DCIS	1.9	16	0	0	5	0	1	75	21	1	81	0.501	0.514	500
Asian, ≥50y	DCIS	1.9	69	0	0	10	2	9	51	81	11	72	0.504	0.554	480
European, <50y	DCIS	1.9	104	1	22	24	10	90	375	139	123	499	0.520	0.522	2342
European, ≥50y	DCIS	1.9	624	85	503	60	187	842	337	956	1617	1426	0.521	0.533	7282
Asian, <50y	Invasive	1.9	133	0	0	27	1	17	470	161	18	515	0.500	0.511	4634
Asian, ≥50y	Invasive	1.9	465	0	0	63	12	105	379	540	117	559	0.505	0.528	4588
European, <50y	Invasive	1.9	964	22	219	239	81	686	3279	1306	1008	4285	0.516	0.520	23224
European, ≥50y	Invasive	1.9	6610	1004	5130	468	1638	6776	2089	9720	14548	10971	0.530	0.548	60461
Asian, <50y	Control	1.9	50	0	0	1	0	13	226	51	13	240	NA	NA	3822
Asian, ≥50y	Control	1.9	259	0	0	12	6	63	203	277	69	284	NA	NA	4525
European, <50y	Control	1.9	228	0	13	34	10	175	1626	272	198	1845	NA	NA	17082
European, ≥50y	Control	1.9	2371	340	4261	169	461	4254	1854	3341	9316	6738	NA	NA	51458
Asian, <50y	DCIS	1.8	18	0	0	6	1	5	69	25	6	81	0.503	0.516	500
Asian, ≥50y	DCIS	1.8	77	0	0	9	4	16	43	90	20	72	0.511	0.556	480
European, <50y	DCIS	1.8	119	3	24	22	12	112	353	156	151	499	0.524	0.523	2342
European, ≥50y	DCIS	1.8	701	132	639	60	221	852	293	1114	1844	1426	0.518	0.538	7282
Asian, <50y	Invasive	1.8	152	0	0	28	3	24	460	183	27	515	0.500	0.510	4634
Asian, ≥50y	Invasive	1.8	548	0	0	64	23	142	330	635	165	559	0.508	0.531	4588
European, <50y	Invasive	1.8	1139	28	241	261	117	842	3065	1545	1228	4285	0.518	0.523	23224
European, ≥50y	Invasive	1.8	7262	1453	6419	493	1903	6792	1783	11111	16567	10971	0.529	0.554	60461
Asian, <50y	Control	1.8	70	0	0	1	0	21	218	71	21	240	NA	NA	3822
Asian, ≥50y	Control	1.8	324	0	0	14	7	81	182	345	88	284	NA	NA	4525
European, <50y	Control	1.8	295	0	15	36	17	256	1536	348	288	1845	NA	NA	17082

European, ≥50y	Control	1.8	2677	515	5711	169	549	4378	1642	3910	11153	6738	NA	NA	51458
Asian, <50y	DCIS	1.7	19	0	0	6	1	6	68	26	7	81	0.503	0.515	500
Asian, ≥50y	DCIS	1.7	83	0	0	7	8	25	32	98	33	72	0.520	0.558	480
European, <50y	DCIS	1.7	147	3	30	22	17	161	299	189	211	499	0.532	0.528	2342
European, ≥50y	DCIS	1.7	730	225	914	58	259	872	237	1272	2270	1426	0.512	0.542	7282
Asian, <50y	Invasive	1.7	196	0	0	31	4	43	437	231	47	515	0.501	0.514	4634
Asian, ≥50y	Invasive	1.7	613	0	0	60	39	183	277	712	222	559	0.510	0.533	4588
European, <50y	Invasive	1.7	1370	38	284	256	194	1132	2703	1858	1648	4285	0.522	0.527	23224
European, ≥50y	Invasive	1.7	7488	2450	9140	456	2245	6869	1401	12639	20704	10971	0.527	0.559	60461
Asian, <50y	Control	1.7	80	0	0	1	0	29	210	81	29	240	NA	NA	3822
Asian, ≥50y	Control	1.7	375	0	0	11	14	117	142	400	131	284	NA	NA	4525
European, <50y	Control	1.7	370	0	16	37	28	412	1368	435	456	1845	NA	NA	17082
European, ≥50y	Control	1.7	2900	934	8712	176	650	4498	1414	4660	14794	6738	NA	NA	51458
Asian, <50y	DCIS	1.6	21	0	0	6	1	6	68	28	7	81	0.503	0.513	500
Asian, ≥50y	DCIS	1.6	96	0	0	8	10	26	28	114	36	72	0.521	0.566	480
European, <50y	DCIS	1.6	174	3	36	22	26	202	249	225	267	499	0.538	0.532	2342
European, ≥50y	DCIS	1.6	785	308	1073	64	300	862	200	1457	2543	1426	0.508	0.546	7282
Asian, <50y	Invasive	1.6	241	0	0	34	5	52	424	280	57	515	0.502	0.516	4634
Asian, ≥50y	Invasive	1.6	706	0	0	63	53	215	228	822	268	559	0.512	0.537	4588
European, <50y	Invasive	1.6	1650	48	313	242	301	1408	2334	2241	2070	4285	0.526	0.533	23224
European, ≥50y	Invasive	1.6	8078	3357	10410	456	2612	6706	1197	14503	23085	10971	0.525	0.566	60461
Asian, <50y	Control	1.6	110	0	0	1	1	30	208	112	31	240	NA	NA	3822
Asian, ≥50y	Control	1.6	446	0	0	12	22	131	119	480	153	284	NA	NA	4525
European, <50y	Control	1.6	453	1	20	35	42	588	1180	531	651	1845	NA	NA	17082
European, ≥50y	Control	1.6	3210	1379	10429	183	780	4521	1254	5552	17109	6738	NA	NA	51458
Asian, <50y	DCIS	1.5	26	0	0	6	1	7	67	33	8	81	0.503	0.516	500
Asian, ≥50y	DCIS	1.5	114	0	0	6	14	27	25	134	41	72	0.524	0.575	480
European, <50y	DCIS	1.5	204	6	40	19	38	230	212	267	314	499	0.543	0.537	2342
European, ≥50y	DCIS	1.5	806	472	1488	56	356	876	138	1690	3192	1426	0.507	0.552	7282
Asian, <50y	Invasive	1.5	290	0	0	38	6	58	413	334	64	515	0.502	0.519	4634
Asian, ≥50y	Invasive	1.5	795	0	0	69	61	225	204	925	286	559	0.512	0.537	4588
European, <50y	Invasive	1.5	1960	72	349	196	436	1643	2010	2664	2500	4285	0.529	0.538	23224
European, ≥50y	Invasive	1.5	8111	4996	13091	432	3017	6633	889	16556	27737	10971	0.517	0.573	60461

Asian, <50y	Control	1.5	131	0	0	1	1	40	198	133	41	240	NA	NA	3822
Asian, ≥50y	Control	1.5	541	0	0	13	26	144	101	580	170	284	NA	NA	4525
European, <50y	Control	1.5	574	1	25	37	61	749	998	673	836	1845	NA	NA	17082
European, ≥50y	Control	1.5	3363	2121	14144	183	954	4605	996	6621	21824	6738	NA	NA	51458
Asian, <50y	DCIS	1.4	36	0	0	9	3	12	57	48	15	81	0.506	0.526	500
Asian, ≥50y	DCIS	1.4	129	0	0	7	14	30	21	150	44	72	0.526	0.581	480
European, <50y	DCIS	1.4	233	11	47	21	52	246	180	317	356	499	0.547	0.543	2342
European, ≥50y	DCIS	1.4	805	680	1828	56	425	843	102	1966	3776	1426	0.505	0.558	7282
Asian, <50y	Invasive	1.4	357	0	0	44	15	91	365	416	106	515	0.502	0.523	4634
Asian, ≥50y	Invasive	1.4	949	0	0	67	78	238	176	1094	316	559	0.514	0.544	4588
European, <50y	Invasive	1.4	2381	103	429	187	566	1800	1732	3237	2898	4285	0.533	0.545	23224
European, ≥50y	Invasive	1.4	8172	6997	14998	370	3545	6341	715	19084	31881	10971	0.509	0.580	60461
Asian, <50y	Control	1.4	155	0	0	4	7	65	164	166	72	240	NA	NA	3822
Asian, ≥50y	Control	1.4	631	0	0	13	35	146	90	679	181	284	NA	NA	4525
European, <50y	Control	1.4	716	4	61	38	91	837	879	849	993	1845	NA	NA	17082
European, ≥50y	Control	1.4	3409	3220	17174	182	1157	4607	792	7968	26158	6738	NA	NA	51458
Asian, <50y	DCIS	1.3	48	0	0	8	5	21	47	61	26	81	0.513	0.532	500
Asian, ≥50y	DCIS	1.3	157	0	0	8	16	32	16	181	48	72	0.526	0.599	480
European, <50y	DCIS	1.3	268	13	55	22	67	256	154	370	391	499	0.550	0.548	2342
European, ≥50y	DCIS	1.3	764	964	2138	49	502	808	67	2279	4412	1426	0.503	0.565	7282
Asian, <50y	Invasive	1.3	434	0	0	36	35	141	303	505	176	515	0.506	0.526	4634
Asian, ≥50y	Invasive	1.3	1108	2	3	56	110	262	131	1276	377	559	0.517	0.550	4588
European, <50y	Invasive	1.3	2846	158	529	167	719	1879	1520	3890	3285	4285	0.537	0.552	23224
European, ≥50y	Invasive	1.3	7914	9379	16633	331	4086	6023	531	21710	36121	10971	0.501	0.588	60461
Asian, <50y	Control	1.3	204	0	0	3	11	89	137	218	100	240	NA	NA	3822
Asian, ≥50y	Control	1.3	742	4	3	10	50	161	63	806	218	284	NA	NA	4525
European, <50y	Control	1.3	902	12	126	47	113	904	781	1074	1155	1845	NA	NA	17082
European, ≥50y	Control	1.3	3235	4639	20270	161	1416	4570	591	9451	30895	6738	NA	NA	51458
Asian, <50y	DCIS	1.2	59	0	0	9	7	23	42	75	30	81	0.516	0.539	500
Asian, ≥50y	DCIS	1.2	180	2	4	7	18	35	12	207	59	72	0.532	0.610	480
European, <50y	DCIS	1.2	302	32	101	26	81	257	135	441	471	499	0.552	0.554	2342
European, ≥50y	DCIS	1.2	689	1342	2355	34	584	767	41	2649	5048	1426	0.507	0.572	7282
Asian, <50y	Invasive	1.2	524	0	0	38	52	147	278	614	199	515	0.507	0.531	4634

Asian, ≥50y	Invasive	1.2	1303	10	17	53	150	261	95	1516	438	559	0.519	0.560	4588
European, <50y	Invasive	1.2	3240	311	900	167	863	1950	1305	4581	4024	4285	0.538	0.559	23224
European, ≥50y	Invasive	1.2	7705	12158	17592	293	4728	5583	367	24884	40061	10971	0.509	0.596	60461
Asian, <50y	Control	1.2	254	0	0	5	14	95	126	273	109	240	NA	NA	3822
Asian, ≥50y	Control	1.2	873	9	23	9	62	170	43	953	264	284	NA	NA	4525
European, <50y	Control	1.2	1094	67	506	52	156	944	693	1369	1673	1845	NA	NA	17082
European, ≥50y	Control	1.2	3126	6286	22521	153	1715	4466	404	11280	34988	6738	NA	NA	51458
Asian, <50y	DCIS	1.1	68	0	0	11	7	27	36	86	34	81	0.518	0.539	500
Asian, ≥50y	DCIS	1.1	191	10	12	8	20	34	10	229	76	72	0.541	0.610	480
European, <50y	DCIS	1.1	337	78	172	25	108	253	113	548	611	499	0.559	0.565	2342
European, ≥50y	DCIS	1.1	525	1811	2606	21	682	707	16	3039	5806	1426	0.507	0.576	7282
Asian, <50y	Invasive	1.1	652	0	0	40	69	152	254	761	221	515	0.508	0.535	4634
Asian, ≥50y	Invasive	1.1	1451	63	101	51	181	245	82	1746	590	559	0.526	0.562	4588
European, <50y	Invasive	1.1	3654	612	1372	178	1045	1899	1163	5489	4928	4285	0.535	0.566	23224
European, ≥50y	Invasive	1.1	5987	16782	19115	189	5510	5078	194	28468	46485	10971	0.507	0.603	60461
Asian, <50y	Control	1.1	335	0	0	4	18	102	116	357	120	240	NA	NA	3822
Asian, ≥50y	Control	1.1	1037	43	71	11	74	159	40	1165	347	284	NA	NA	4525
European, <50y	Control	1.1	1339	181	1059	55	209	977	604	1784	2426	1845	NA	NA	17082
European, ≥50y	Control	1.1	2537	8838	25104	118	2134	4255	231	13627	40331	6738	NA	NA	51458
Asian, <50y	DCIS	1	83	0	0	9	11	29	32	103	40	81	0.522	0.541	500
Asian, ≥50y	DCIS	1	203	20	24	6	26	31	9	255	101	72	0.545	0.610	480
European, <50y	DCIS	1	357	148	280	23	134	251	91	662	813	499	0.569	0.573	2342
European, ≥50y	DCIS	1	401	2301	2615	15	776	625	10	3493	6317	1426	0.504	0.580	7282
Asian, <50y	Invasive	1	834	0	0	39	97	169	210	970	266	515	0.511	0.543	4634
Asian, ≥50y	Invasive	1	1579	185	237	40	234	226	59	2038	882	559	0.536	0.567	4588
European, <50y	Invasive	1	3925	1204	1966	164	1252	1893	976	6545	6315	4285	0.531	0.573	23224
European, ≥50y	Invasive	1	4609	21400	18819	144	6288	4420	119	32441	50927	10971	0.509	0.608	60461
Asian, <50y	Control	1	448	0	0	2	25	114	99	475	139	240	NA	NA	3822
Asian, ≥50y	Control	1	1199	106	197	10	89	154	31	1404	546	284	NA	NA	4525
European, <50y	Control	1	1576	431	1846	57	268	1026	494	2332	3571	1845	NA	NA	17082
European, ≥50y	Control	1	1993	11833	25873	82	2592	3956	108	16500	44254	6738	NA	NA	51458
Asian, <50y	DCIS	0.9	101	0	1	8	16	29	28	125	46	81	0.525	0.545	500
Asian, ≥50y	DCIS	0.9	212	38	30	7	28	31	6	285	127	72	0.549	0.609	480

European, <50y	DCIS	0.9	353	273	411	23	172	237	67	821	1093	499	0.575	0.587	2342
European, ≥50y	DCIS	0.9	250	2880	2440	6	899	517	4	4035	6736	1426	0.504	0.583	7282
Asian, <50y	Invasive	0.9	1006	3	22	41	119	168	187	1169	312	515	0.513	0.546	4634
Asian, ≥50y	Invasive	0.9	1732	312	293	29	282	210	38	2355	1097	559	0.537	0.569	4588
European, <50y	Invasive	0.9	4019	2202	2712	144	1524	1812	805	7889	8250	4285	0.519	0.581	23224
European, ≥50y	Invasive	0.9	3322	26277	17266	105	7110	3702	54	36814	54355	10971	0.509	0.610	60461
Asian, <50y	Control	0.9	573	3	12	3	34	108	95	613	157	240	NA	NA	3822
Asian, ≥50y	Control	0.9	1385	198	288	4	114	151	15	1701	751	284	NA	NA	4525
European, <50y	Control	0.9	1638	979	3028	54	351	1048	392	3022	5406	1845	NA	NA	17082
European, ≥50y	Control	0.9	1429	15404	25083	43	3093	3564	38	19969	47144	6738	NA	NA	51458
Asian, <50y	DCIS	0.8	117	9	10	9	21	28	23	156	68	81	0.519	0.548	500
Asian, ≥50y	DCIS	0.8	192	89	43	8	35	25	4	324	192	72	0.545	0.610	480
European, <50y	DCIS	0.8	309	466	457	20	203	229	47	998	1355	499	0.575	0.598	2342
European, ≥50y	DCIS	0.8	120	3533	2079	0	1018	407	1	4671	7037	1426	0.505	0.587	7282
Asian, <50y	Invasive	0.8	1099	122	153	43	148	166	158	1412	589	515	0.514	0.544	4634
Asian, ≥50y	Invasive	0.8	1602	791	495	20	336	177	26	2749	1799	559	0.541	0.572	4588
European, <50y	Invasive	0.8	3914	3524	3235	129	1842	1664	650	9409	10265	4285	0.507	0.587	23224
European, ≥50y	Invasive	0.8	2045	31500	14648	52	7930	2966	23	41527	57044	10971	0.507	0.610	60461
Asian, <50y	Control	0.8	707	72	150	3	47	109	81	829	378	240	NA	NA	3822
Asian, ≥50y	Control	0.8	1368	545	592	7	138	131	8	2058	1406	284	NA	NA	4525
European, <50y	Control	0.8	1607	1779	4068	62	486	989	308	3934	7322	1845	NA	NA	17082
European, ≥50y	Control	0.8	904	19481	23035	17	3628	3083	10	24030	49227	6738	NA	NA	51458
Asian, <50y	DCIS	0.7	135	20	16	8	25	28	20	188	89	81	0.510	0.548	500
Asian, ≥50y	DCIS	0.7	158	157	46	6	46	19	1	367	268	72	0.537	0.609	480
European, <50y	DCIS	0.7	251	688	501	16	258	194	31	1213	1641	499	0.571	0.609	2342
European, ≥50y	DCIS	0.7	40	4128	1647	0	1116	310	0	5284	7201	1426	0.503	0.584	7282
Asian, <50y	Invasive	0.7	1262	252	218	26	202	164	123	1742	836	515	0.511	0.548	4634
Asian, ≥50y	Invasive	0.7	1343	1396	643	15	396	143	5	3150	2578	559	0.539	0.570	4588
European, <50y	Invasive	0.7	3696	5295	3463	115	2177	1501	492	11283	12436	4285	0.512	0.593	23224
European, ≥50y	Invasive	0.7	979	36495	11506	22	8749	2192	8	46245	58942	10971	0.504	0.603	60461
Asian, <50y	Control	0.7	826	172	258	1	68	104	67	1067	602	240	NA	NA	3822
Asian, ≥50y	Control	0.7	1231	1079	833	4	161	115	4	2475	2188	284	NA	NA	4525
European, <50y	Control	0.7	1446	2973	5011	47	642	931	225	5108	9557	1845	NA	NA	17082

European, ≥50y	Control	0.7	378	24125	19725	2	4233	2501	2	28738	50584	6738	NA	NA	51458
Asian, <50y	DCIS	0.6	112	75	44	7	29	31	14	223	179	81	0.514	0.539	500
Asian, ≥50y	DCIS	0.6	119	218	46	4	53	15	0	394	332	72	0.526	0.585	480
European, <50y	DCIS	0.6	173	962	455	8	320	148	23	1463	1885	499	0.559	0.619	2342
European, ≥50y	DCIS	0.6	0	4649	1207	0	1238	188	0	5887	7282	1426	0.500	0.573	7282
Asian, <50y	Invasive	0.6	948	866	468	20	249	150	96	2083	1733	515	0.506	0.541	4634
Asian, ≥50y	Invasive	0.6	1021	2062	621	7	441	110	1	3531	3234	559	0.532	0.560	4588
European, <50y	Invasive	0.6	3038	7623	3492	73	2552	1277	383	13286	14944	4285	0.521	0.593	23224
European, ≥50y	Invasive	0.6	0	41244	8246	0	9511	1460	0	50755	60461	10971	0.500	0.588	60461
Asian, <50y	Control	0.6	649	672	617	1	83	101	55	1405	1473	240	NA	NA	3822
Asian, ≥50y	Control	0.6	1003	1742	875	4	192	87	1	2941	2896	284	NA	NA	4525
European, <50y	Control	0.6	1185	4545	5512	35	841	825	144	6606	11723	1845	NA	NA	17082
European, ≥50y	Control	0.6	0	29239	15481	0	4883	1855	0	34122	51458	6738	NA	NA	51458
Asian, <50y	DCIS	0.5	102	116	45	6	41	25	9	265	227	81	0.524	0.524	500
Asian, ≥50y	DCIS	0.5	92	267	38	2	57	13	0	418	375	72	0.508	0.555	480
European, <50y	DCIS	0.5	82	1238	367	4	372	113	10	1696	2090	499	0.542	0.613	2342
European, ≥50y	DCIS	0.5	0	5110	746	0	1323	103	0	6433	7282	1426	0.500	0.556	7282
Asian, <50y	Invasive	0.5	962	1246	454	14	303	129	69	2525	2132	515	0.521	0.531	4634
Asian, ≥50y	Invasive	0.5	674	2765	470	2	491	66	0	3932	3792	559	0.514	0.548	4588
European, <50y	Invasive	0.5	1587	10900	3183	43	2962	1016	264	15492	18061	4285	0.516	0.585	23224
European, ≥50y	Invasive	0.5	0	44582	4908	0	10158	813	0	54740	60461	10971	0.500	0.567	60461
Asian, <50y	Control	0.5	636	1099	626	1	106	88	45	1842	1919	240	NA	NA	3822
Asian, ≥50y	Control	0.5	648	2562	767	1	231	52	0	3442	3612	284	NA	NA	4525
European, <50y	Control	0.5	575	6822	5269	19	1089	643	94	8505	13823	1845	NA	NA	17082
European, ≥50y	Control	0.5	0	34142	10578	0	5562	1176	0	39704	51458	6738	NA	NA	51458

Supplementary Table S13. P-values for model calibration performance from the predtools package in R, assessed using: (A) mean calibration (predicted vs outcome event rate), (B) model-based receiver operating characteristic
(mROC)/ROC equality, and (U) unified calibration testing both A and B. p-values were computed based on the reference population (European, ≥50y), for all groups (European, <50y; Asian, ≥50y; Asian, <50y), and for both polygenic risk score (PRS) and the Gail model. A small p-value for each of the test indicates: (A) miscalibration of average predicted risk, (B) miscalibration in distribution of predicted risk, and (U) overall miscalibration.

		**Gail model**	
Groups	A	B	U
Asian, <50y	<2.2E-16	<2.2E-16	<2.2E-16
Asian, ≥50y	3.00E-05	0.00032	1.85E-07
European, <50y	<2.2E-16	<2.2E-16	<2.2E-16

		**PRS model**	
Groups	A	B	U
Asian, <50y	<2.2E-16	<2.2E-16	<2.2E-16
Asian, ≥50y	9.00E-05	0.00025	4.37E-07
European, <50y	<2.2E-16	<2.2E-16	<2.2E-16

Supplementary Table S14. Discriminatory ability of risk factor combinations in the Gail model, corresponding to **Figure 3**. Five-year absolute risk was calculated using the R package "BCRA" and used to predict the risk of invasive breast cancer case-control. AUC: Area under the receiver operator curve, CI: Confidence interval, AgeMen: Age at menarche, Age1st: Age at first live birth, N_Rels: Number of first-degree relatives with breast cancer, N_Biop: Number of prior biopsies.

Ancestry	Age group, years	Variables included in model	AUC (95%CI)
European	<50	AgeMen	0.515 (0.509 to 0.521)
	<50	Age1st	0.539 (0.533 to 0.544)
	<50	N_Rels	0.530 (0.525 to 0.536)
	<50	N_Biop	0.511 (0.505 to 0.517)
	<50	AgeMen + Age1st	0.541 (0.536 to 0.547)
	<50	AgeMen + N_Rels	0.524 (0.518 to 0.529)
	<50	AgeMen + N_Biop	0.504 (0.498 to 0.510)
	<50	Age1st + N_Rels	0.505 (0.499 to 0.511)
	<50	Age1st + N_Biop	0.521 (0.516 to 0.527)
	<50	N_Rels + N_Biop	0.545 (0.540 to 0.551)
	<50	AgeMen + Age1st + N_Rels	0.508 (0.502 to 0.513)
	<50	AgeMen + Age1st + N_Biop	0.524 (0.518 to 0.530)
	<50	AgeMen + N_Rels + N_Biop	0.539 (0.533 to 0.544)
	<50	Age1st + N_Rels + N_Biop	0.490 (0.485 to 0.496)
	<50	AgeMen + Age1st + N_Rels + N_Biop	0.493 (0.487 to 0.499)
	<50	Full_model	0.493 (0.487 to 0.499)
	≥50	AgeMen	0.509 (0.505 to 0.512)
	≥50	Age1st	0.516 (0.513 to 0.520)
	≥50	N_Rels	0.543 (0.540 to 0.547)
	≥50	N_Biop	0.542 (0.539 to 0.546)
	≥50	AgeMen + Age1st	0.522 (0.519 to 0.526)
	≥50	AgeMen + N_Rels	0.532 (0.529 to 0.536)
	≥50	AgeMen + N_Biop	0.530 (0.526 to 0.533)
	≥50	Age1st + N_Rels	0.510 (0.507 to 0.514)
	≥50	Age1st + N_Biop	0.501 (0.497 to 0.504)
	≥50	N_Rels + N_Biop	0.559 (0.555 to 0.562)
	≥50	AgeMen + Age1st + N_Rels	0.505 (0.501 to 0.508)
	≥50	AgeMen + Age1st + N_Biop	0.507 (0.504 to 0.510)
	≥50	AgeMen + N_Rels + N_Biop	0.548 (0.544 to 0.551)
	≥50	Age1st + N_Rels + N_Biop	0.522 (0.519 to 0.526)
	≥50	AgeMen + Age1st + N_Rels + N_Biop	0.517 (0.514 to 0.520)
	≥50	Full_model	0.517 (0.514 to 0.520)
Asian	<50	AgeMen	0.536 (0.524 to 0.548)
	<50	Age1st	0.543 (0.530 to 0.555)
	<50	N_Rels	0.486 (0.473 to 0.498)
	<50	N_Biop	0.535 (0.522 to 0.547)
	<50	AgeMen + Age1st	0.542 (0.530 to 0.554)
	<50	AgeMen + N_Rels	0.514 (0.502 to 0.526)
	<50	AgeMen + N_Biop	0.536 (0.524 to 0.548)
	<50	Age1st + N_Rels	0.524 (0.511 to 0.536)
	<50	Age1st + N_Biop	0.543 (0.530 to 0.555)

<50	N_Rels + N_Biop	0.486 (0.473 to 0.498)
<50	AgeMen + Age1st + N_Rels	0.523 (0.511 to 0.535)
<50	AgeMen + Age1st + N_Biop	0.542 (0.530 to 0.554)
<50	AgeMen + N_Rels + N_Biop	0.514 (0.502 to 0.526)
<50	Age1st + N_Rels + N_Biop	0.524 (0.511 to 0.536)
<50	AgeMen + Age1st + N_Rels + N_Biop	0.523 (0.511 to 0.535)
<50	Full_model	0.523 (0.511 to 0.535)
≥50	AgeMen	0.510 (0.498 to 0.522)
≥50	Age1st	0.537 (0.525 to 0.549)
≥50	N_Rels	0.534 (0.522 to 0.545)
≥50	N_Biop	0.512 (0.500 to 0.523)
≥50	AgeMen + Age1st	0.536 (0.524 to 0.548)
≥50	AgeMen + N_Rels	0.531 (0.519 to 0.543)
≥50	AgeMen + N_Biop	0.510 (0.498 to 0.522)
≥50	Age1st + N_Rels	0.556 (0.544 to 0.568)
≥50	Age1st + N_Biop	0.537 (0.525 to 0.549)
≥50	N_Rels + N_Biop	0.534 (0.522 to 0.545)
≥50	AgeMen + Age1st + N_Rels	0.554 (0.543 to 0.566)
≥50	AgeMen + Age1st + N_Biop	0.536 (0.524 to 0.548)
≥50	AgeMen + N_Rels + N_Biop	0.531 (0.519 to 0.543)
≥50	Age1st + N_Rels + N_Biop	0.556 (0.544 to 0.568)
≥50	AgeMen + Age1st + N_Rels + N_Biop	0.554 (0.543 to 0.566)
≥50	Full_model	0.554 (0.543 to 0.566)

Supplementary Figure S1. Flowchart of individuals selected.

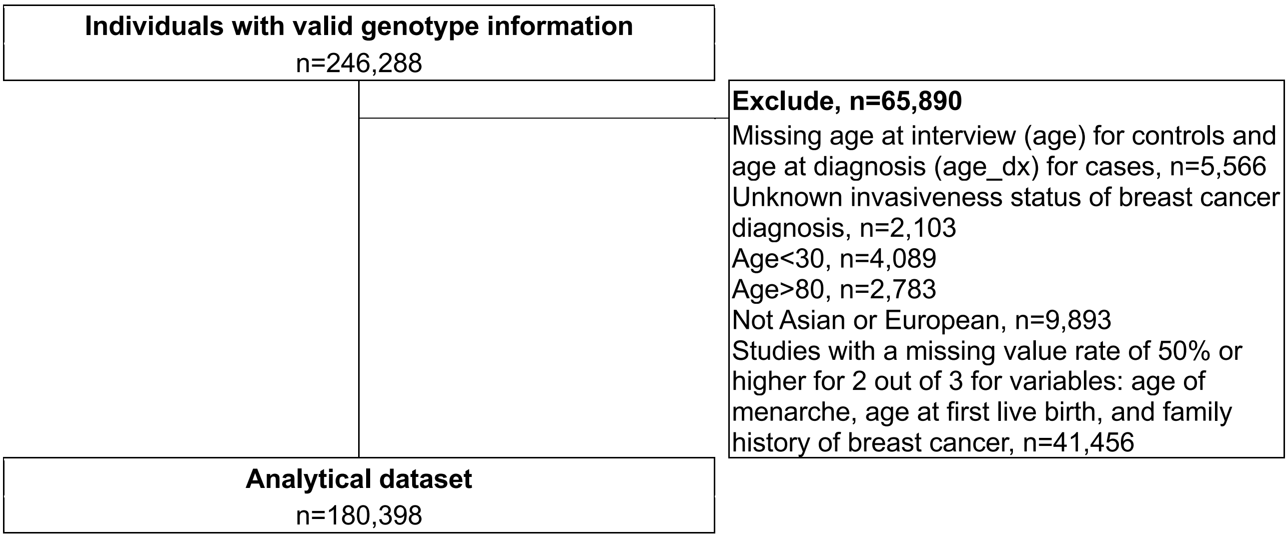

Supplementary Figure S2. Distribution of polygenic risk scores (scoresum) and five-year absolute risk by country, for individuals of European-ancestry.

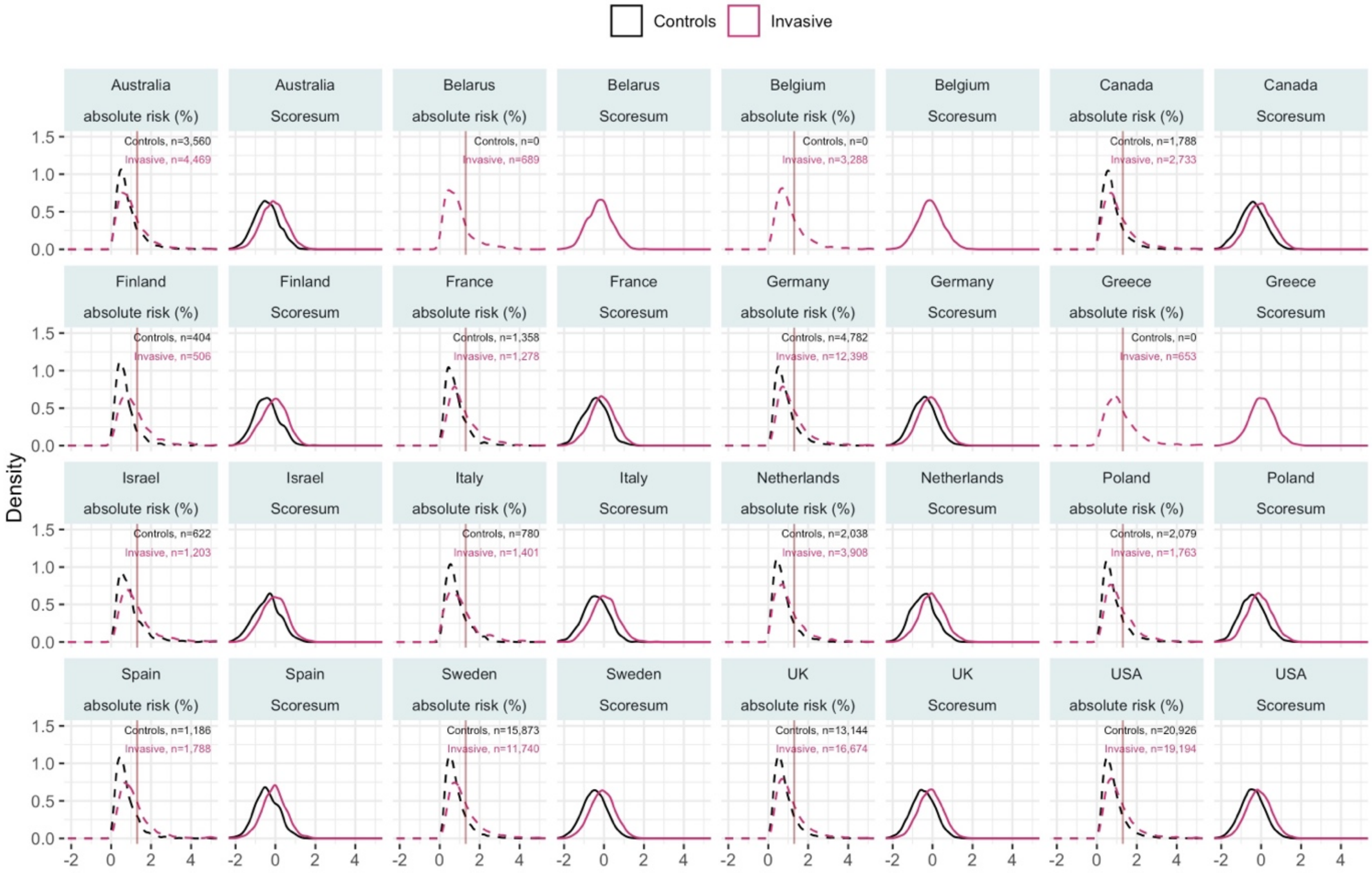

Supplementary Figure S3. Distribution of polygenic risk scores (scoresum) and 5-year absolute risk by country, for individuals of Asian-ancestry.

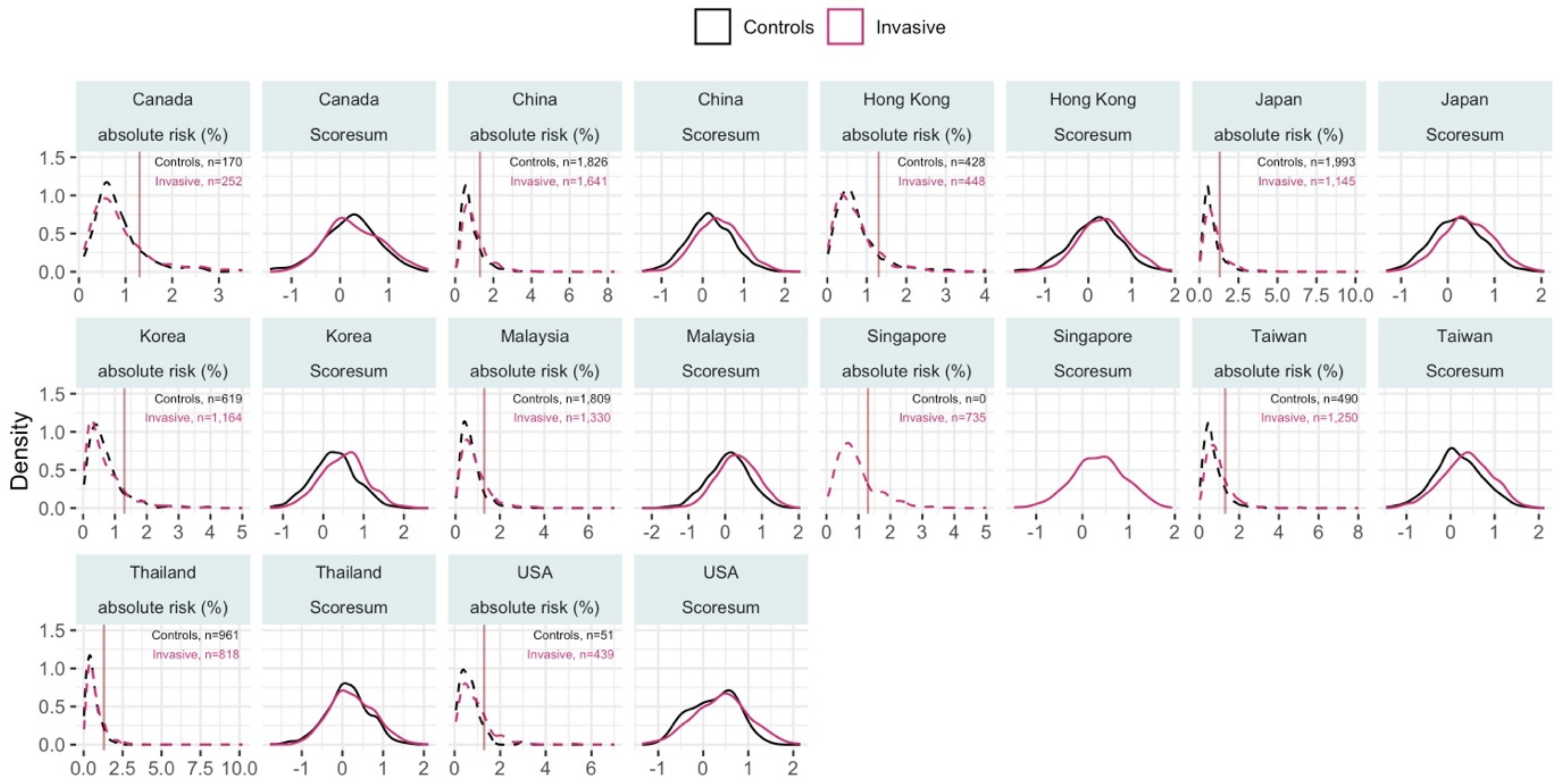

Supplementary Figure S4. Sensitivity analysis of the discriminatory ability of the Gail model, assuming that missing values correspond to the highest risk category rather than the reference category. Five-year absolute risk was estimated using the R package BCRA and applied to predict the risk of invasive breast cancer in the case-control dataset. Dots represent models in which missing values for each risk factor were assigned to the highest risk group, while "X" denotes the model using the conventional approach of
assigning missing values to [illegible]

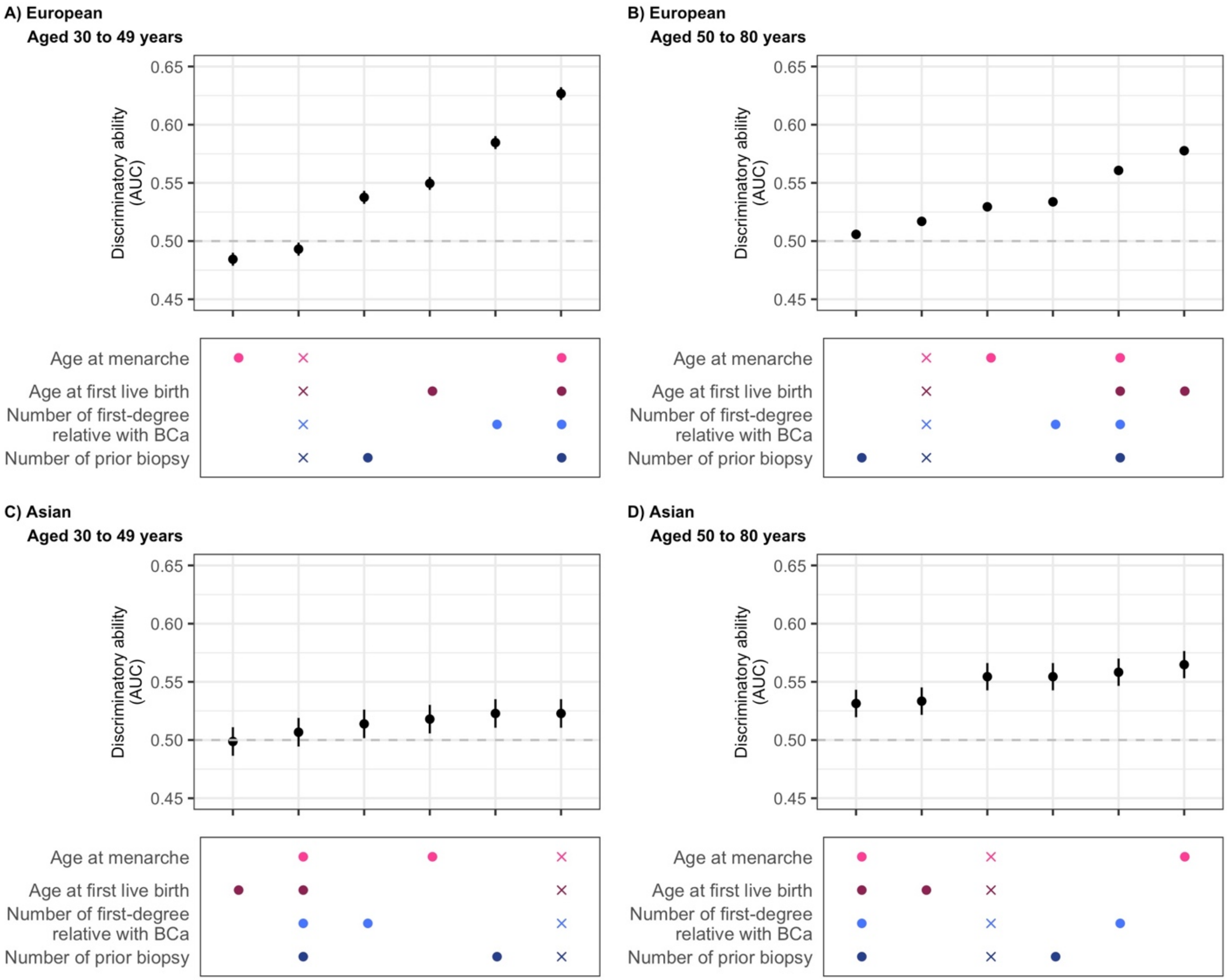

Supplementary Figure S5. Sensitivity analysis of the discriminatory ability of the Gail model, assuming that missing values correspond to the highest risk category rather than the reference category. Five-year absolute risk was estimated using the R package BCRA and applied to predict the risk of DCIS in the case-control dataset. Dots represent models in which missing values for each risk factor were assigned to the highest risk group, while “X” denotes the model that using the conventional approach of assigning
missing values to the lowest risk group. European: women of European ancestry; Asian: women of Asian ancestry.

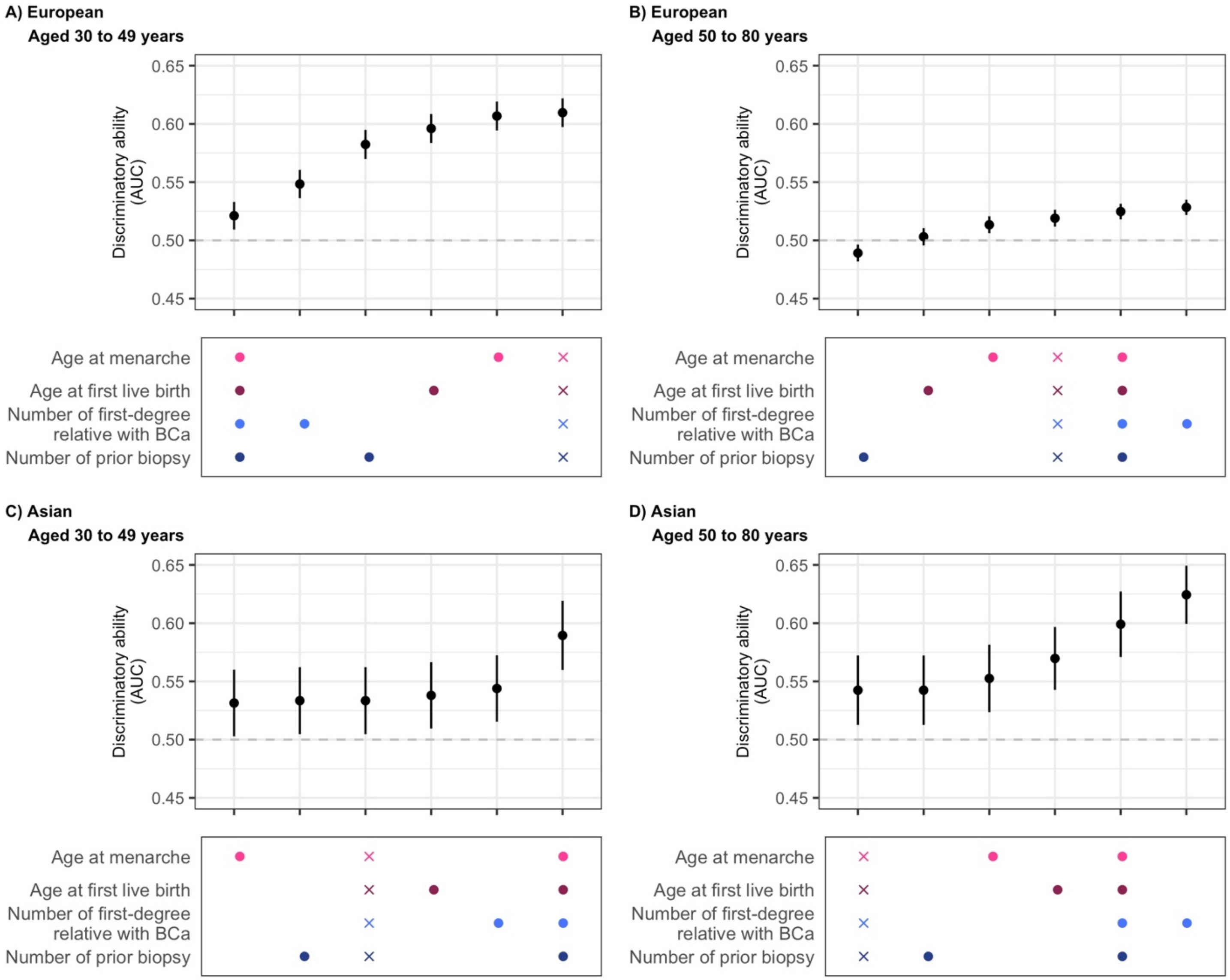

Supplementary Figure S6. Proportion of individuals identified as high-risk by the polygenic risk score (PRS), the Gail model, and family history (FH) at varying absolute risk thresholds for PRS and Gail. Each line represents one identification method (black: PRS only; grey: Gail only; orange: FH only).

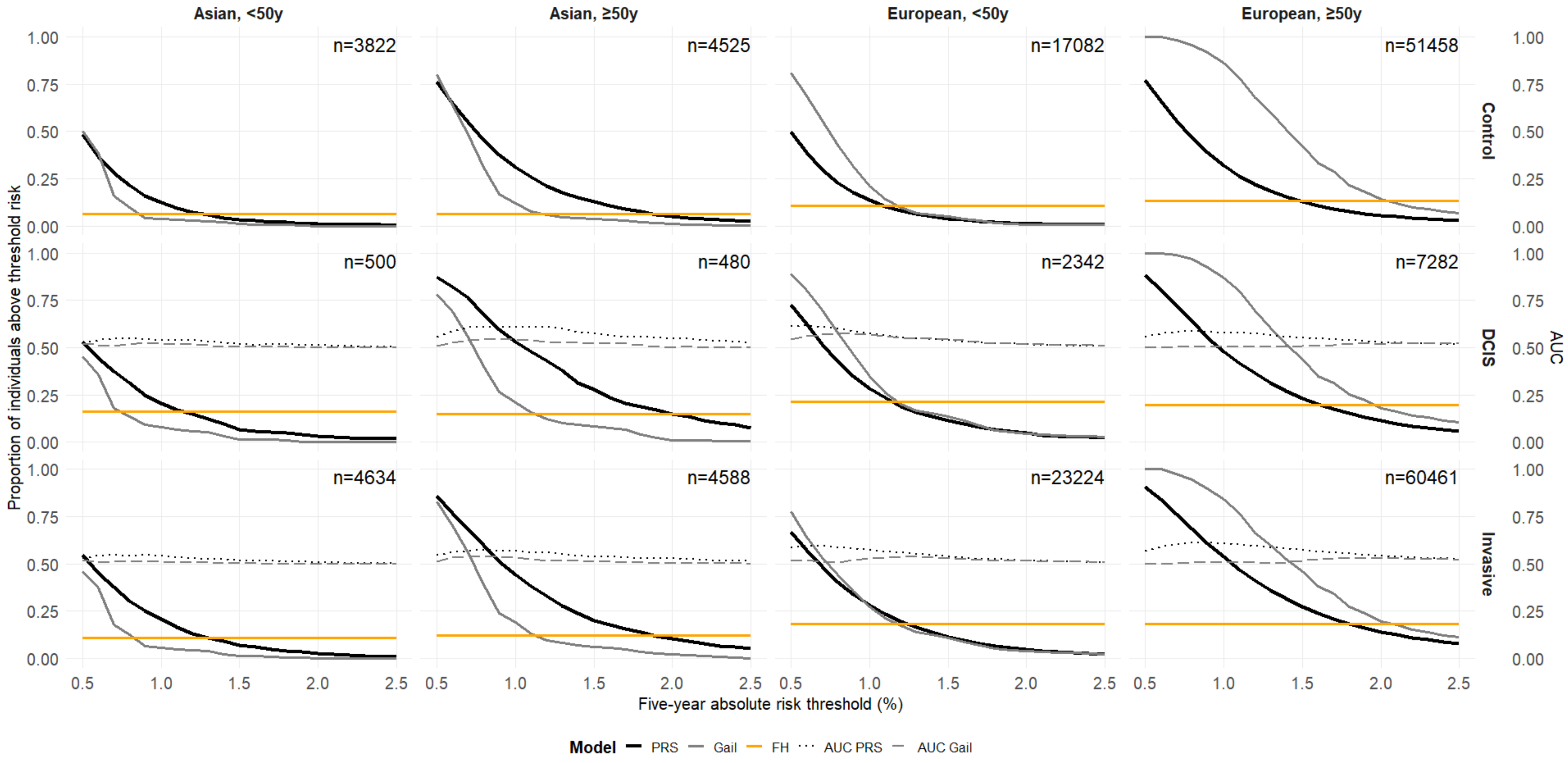

Supplementary Figure S7. Proportion of individuals identified as high-risk by multiple factors across varying absolute-risk thresholds. PRS: polygenic risk score; Gail: the Gail model; FH: family history. These values match the overlapping regions from the corresponding Venn diagrams for each threshold.

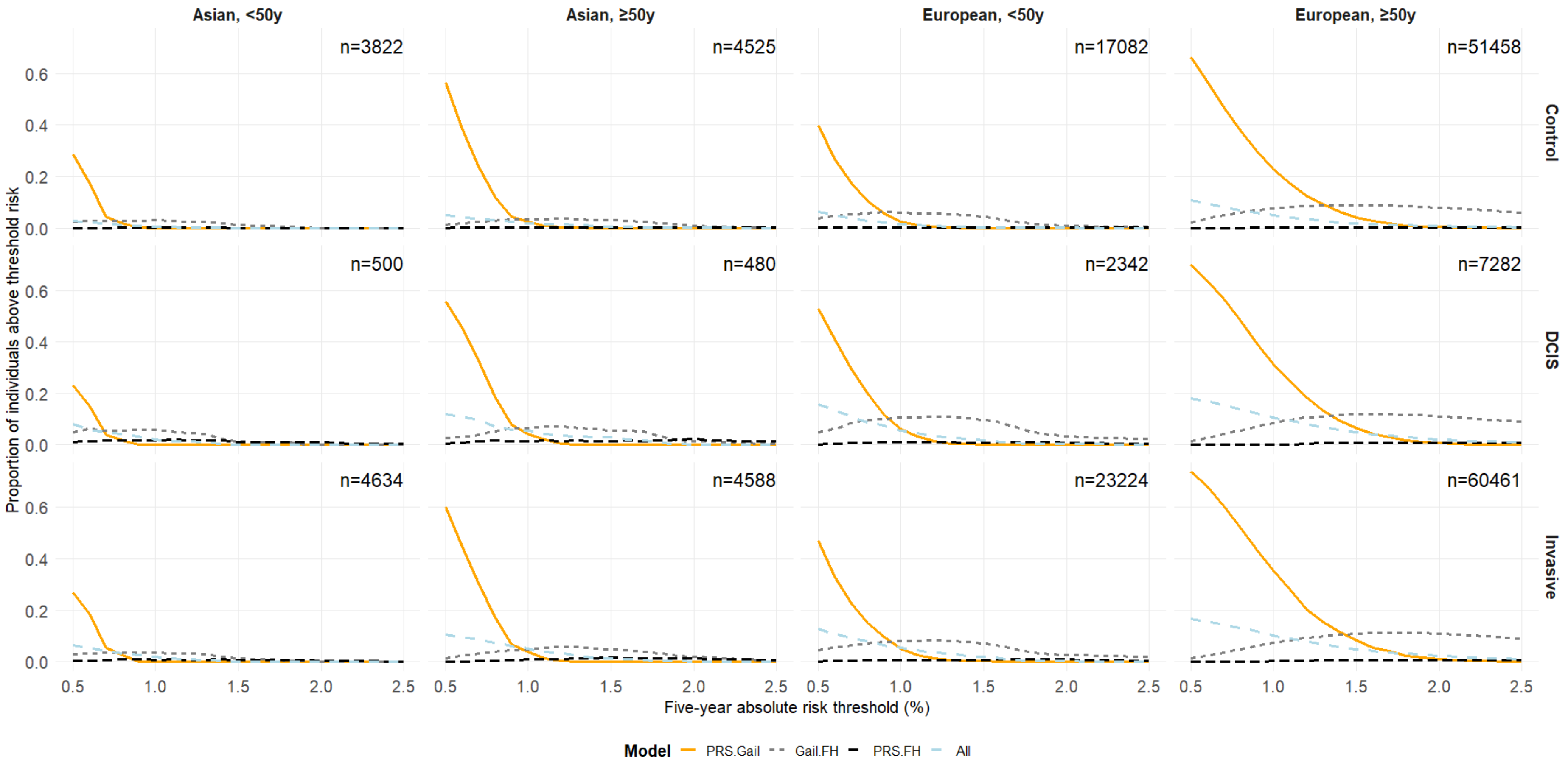

Supplementary Figure S8. Proportion of individuals identified as at high risk by the breast cancer polygenic risk score (PRS) and the Gail model (GAIL), by country and age. The proportion of individuals identified as high risk by both criteria is indicated in grey. PRS: 5-year absolute risk using polygenic risk score ≥1·66%. GAIL: 5-year absolute risk using the Gail model ≥1.66%. Numbers adjacent to the bars represent the number of high-risk individuals identified by respective risk tools. European: women of European-ancestry; Asian: women of Asian-ancestry.

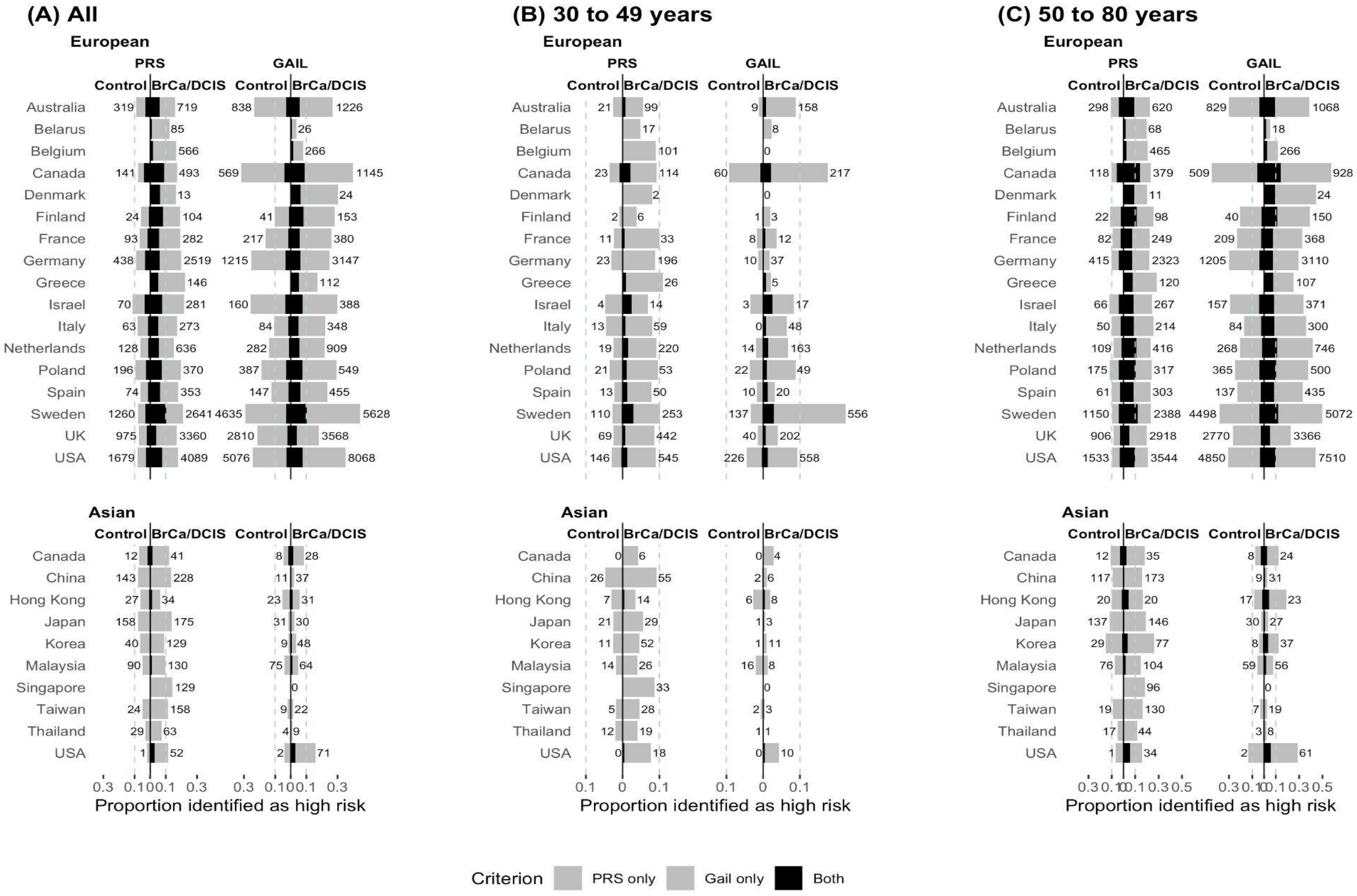

BCAC Funding and Acknowledgments

Funding

This work was supported by Cancer Research UK grant: PPRPGM-Nov20\100002 and by core funding from the NIHR Cambridge Biomedical Research Centre (NIHR203312) [*]. *The views expressed are those of the author(s) and not necessarily those of the NIHR or the Department of Health and Social Care. Additional funding for BCAC is provided by the Confluence project which is funded with intramural funds from the National Cancer Institute Intramural Research Program, National Institutes of Health, the European Union's Horizon 2020 Research and Innovation Programme (grant numbers 634935 for BRIDGES), and the PERSPECTIVE I&I project, funded by the Government of Canada through Genome Canada and the Canadian Institutes of Health Research, the Ministère de l'Économie et de l'Innovation du Québec through Genome Québec, the Quebec Breast Cancer Foundation.

Genotyping of the OncoArray was funded by the NIH Grant U19 CA148065, and Cancer Research UK Grant C1287/A16563 and the PERSPECTIVE project supported by the Government of Canada through Genome Canada and the Canadian Institutes of Health Research (grant GPH-129344) and, the Ministère de l'Économie, Science et Innovation du Québec through Genome Québec and the PSRSIIRI-701 grant, and the Quebec Breast Cancer Foundation. Funding for iCOGS came from: the European Community's Seventh Framework Programme under grant agreement n° 223175 (HEALTH-F2-2009-223175) (COGS), Cancer Research UK (C1287/A10118, C1287/A10710, C12292/A11174, C1281/A12014, C5047/A8384, C5047/A15007, C5047/A10692, C8197/A16565), the National Institutes of Health (CA128978) and Post-Cancer GWAS initiative (1U19 CA148537, 1U19 CA148065 and 1U19 CA148112 - the GAME-ON initiative), the Department of Defence (W81XWH-10-1-0341), the Canadian Institutes of Health Research (CIHR) for the CIHR Team in Familial Risks of Breast Cancer, and Komen Foundation for the Cure, the Breast Cancer Research Foundation, and the Ovarian Cancer Research Fund.

The BRIDGES panel sequencing was supported by the European Union Horizon 2020 research and innovation program BRIDGES (grant number, 634935) and the Wellcome Trust (v203477/Z/16/Z). The ABCFS was also supported by the National Health and Medical Research Council of Australia, the New South Wales Cancer Council, the Victorian Health Promotion Foundation (Australia) and the Victorian Breast Cancer Research Consortium. J.L.H. is a National Health and Medical Research Council (NHMRC) Senior Principal Research Fellow. M.C.S. is a NHMRC Senior Research Fellow. The ABCS study was supported by the Dutch Cancer Society [grants NKI 2007-3839; 2009 4363] and an institutional grant of the Dutch Cancer Society and of the Dutch Ministry of Health, Welfare and Sport. The Australian Breast Cancer Tissue Bank (ABCTB) was supported by the National Health and Medical Research Council of Australia, The Cancer Institute NSW and the National Breast Cancer Foundation. The ACP study is funded by the Breast Cancer Research Trust, UK. KM and AL are supported by the NIHR Manchester Biomedical Research Centre, the Allan Turing Institute under the EPSRC grant EP/N510129/1. The AHS study is supported by the intramural

research program of the National Institutes of Health, the National Cancer Institute (grant number Z01-CP010119), and the National Institute of Environmental Health Sciences (grant number Z01-ES049030). The work of the BBCC was partly funded by ELAN-Fond of the University Hospital of Erlangen. The BBCS is funded by Cancer Research UK and Breast Cancer Now and acknowledges NHS funding to the NIHR Biomedical Research Centre, and the National Cancer Research Network (NCRN). The BCEES was funded by the National Health and Medical Research Council, Australia and the Cancer Council Western Australia (JS). For the BCFR-NY, BCFR-PA, BCFR-UT this work was supported by grant UM1 CA164920 from the National Cancer Institute. This work was supported by grant U01 CA164920 from the USA National Cancer Institute. The content of this manuscript does not necessarily reflect the views or policies of the National Cancer Institute or any of the collaborating centers in the Breast Cancer Family Registry (BCFR), nor does mention of trade names, commercial products, or organizations imply endorsement by the US Government or the BCFR. The BCINIS study is supported in part by the Breast Cancer Research Foundation (BCRF). The BREast Oncology GAlician Network (BREOGAN) is funded by Acción Estratégica de Salud del Instituto de Salud Carlos III FIS PI12/02125/Cofinanciado and FEDER PI17/00918/Cofinanciado FEDER; Acción Estratégica de Salud del Instituto de Salud Carlos III FIS Intrasalud (PI13/01136); Programa Grupos Emergentes, Cancer Genetics Unit, Instituto de Investigacion Biomedica Galicia Sur. Xerencia de Xestion Integrada de Vigo-SERGAS, Instituto de Salud Carlos III, Spain; Grant 10CSA012E, Consellería de Industria Programa Sectorial de Investigación Aplicada, PEME I + D e I + D Suma del Plan Gallego de Investigación, Desarrollo e Innovación Tecnológica de la Consellería de Industria de la Xunta de Galicia, Spain; Grant EC11-192. Fomento de la Investigación Clínica Independiente, Ministerio de Sanidad, Servicios Sociales e Igualdad, Spain; and Grant FEDER-Innterconecta. Ministerio de Economia y Competitividad, Xunta de Galicia, Spain. CBCS is funded by the Canadian Cancer Society (grant # 313404) and the Canadian Institutes of Health Research. CCGP is supported by funding from the University of Crete. The CECILE study was supported by Fondation de France, Institut National du Cancer (INCa), Ligue Nationale contre le Cancer, Agence Nationale de Sécurité Sanitaire, de l'Alimentation, de l'Environnement et du Travail (ANSES), Agence Nationale de la Recherche (ANR). The CGPS was supported by the Chief Physician Johan Boserup and Lise Boserup Fund, the Danish Medical Research Council, and Herlev and Gentofte Hospital. The American Cancer Society funds the creation, maintenance, and updating of the CPS-II cohort. The California Teachers Study (CTS) and the research reported in this publication were supported by the National Cancer Institute of the National Institutes of Health under award number U01-CA199277; P30-CA033572; P30-CA023100; UM1-CA164917; and R01-CA077398. The content is solely the responsibility of the authors and does not necessarily represent the official views of the National Cancer Institute or the National Institutes of Health. The collection of cancer incidence data used in the California Teachers Study was supported by the California Department of Public Health pursuant to California Health and Safety Code Section 103885; Centers for Disease Control and Prevention's National Program of Cancer Registries, under cooperative agreement 5NU58DP006344; the National Cancer Institute's Surveillance, Epidemiology and End Results Program under contract HHSN261201800032I awarded to the University of California, San Francisco, contract

HHSN261201800015I awarded to the University of Southern California, and contract HHSN261201800009I awarded to the Public Health Institute. The opinions, findings, and conclusions expressed herein are those of the author(s) and do not necessarily reflect the official views of the State of California, Department of Public Health, the National Cancer Institute, the National Institutes of Health, the Centers for Disease Control and Prevention or their Contractors and Subcontractors, or the Regents of the University of California, or any of its programs. The University of Westminster curates the DietCompLyf database funded by Against Breast Cancer Registered Charity No. 1121258 and the NCRN. The coordination of EPIC is financially supported by the European Commission (DG-SANCO) and the International Agency for Research on Cancer. The national cohorts are supported by: Ligue Contre le Cancer, Institut Gustave Roussy, Mutuelle Générale de l'Education Nationale, Institut National de la Santé et de la Recherche Médicale (INSERM) (France); German Cancer Aid, German Cancer Research Center (DKFZ), Federal Ministry of Education and Research (BMBF) (Germany); the Hellenic Health Foundation, the Stavros Niarchos Foundation (Greece); Fondazione AIRC per la Ricerca sul Cancro ETS and National Research Council (Italy); Dutch Ministry of Public Health, Welfare and Sports (VWS), Netherlands Cancer Registry (NKR), LK Research Funds, Dutch Prevention Funds, Dutch ZON (Zorg Onderzoek Nederland), World Cancer Research Fund (WCRF), Statistics Netherlands (The Netherlands); Health Research Fund (FIS), PI13/00061 to Granada, PI13/01162 to EPIC-Murcia, Regional Governments of Andalucía, Asturias, Basque Country, Murcia and Navarra, ISCIII RETIC (RD06/0020) (Spain); Cancer Research UK (14136 to EPIC-Norfolk; C570/A16491 and C8221/A19170 to EPIC-Oxford), Medical Research Council (1000143 to EPIC-Norfolk, MR/M012190/1 to EPIC-Oxford) (United Kingdom). The ESTHER study was supported by a grant from the Baden Württemberg Ministry of Science, Research and Arts. Additional cases were recruited in the context of the VERDI study, which was supported by a grant from the German Cancer Aid (Deutsche Krebshilfe). FHRISK and PROCAS are funded from NIHR grant PGfAR 0707-10031. DGE, AH and WGN are supported by the NIHR Manchester Biomedical Research Centre (IS-BRC-1215-20007). The GENICA was funded by the Federal Ministry of Education and Research (BMBF) Germany grants 01KW9975/5, 01KW9976/8, 01KW9977/0 and 01KW0114, the Robert Bosch Foundation, Stuttgart, Deutsches Krebsforschungszentrum (DKFZ), Heidelberg, the Institute for Prevention and Occupational Medicine of the German Social Accident Insurance, Institute of the Ruhr University Bochum (IPA), Bochum, as well as the Department of Internal Medicine, Johanniter GmbH Bonn, Johanniter Krankenhaus, Bonn, Germany. The GESBC was supported by the Deutsche Krebshilfe e. V. [70492] and the German Cancer Research Center (DKFZ). The HABCS study was supported by German Research Foundation (DFG Do761/15-1), the Claudia von Schilling Foundation for Breast Cancer Research, by the Lower Saxonian Cancer Society, and by the Rudolf Bartling Foundation. The HERPACC was supported by MEXT Kakenhi (No. 170150181 and 26253041) from the Ministry of Education, Science, Sports, Culture and Technology of Japan, by a Grant-in-Aid for the Third Term Comprehensive 10-Year Strategy for Cancer Control from Ministry Health, Labour and Welfare of Japan, by Health and Labour Sciences Research Grants for Research on Applying Health Technology from Ministry Health, Labour and Welfare of Japan, by National Cancer Center Research and Development Fund, and "Practical Research for Innovative Cancer Control (15ck0106177h0001

and 20ck0106553)" from Japan Agency for Medical Research and development, AMED, and Cancer Bio Bank Aichi. The HMBCS was supported by the German Research Foundation (DFG Do761/15-1), a grant from the Friends of Hannover Medical School, and by the Rudolf Bartling Foundation. ICICLE was supported by Breast Cancer Now, CRUK and Biomedical Research Centre at Guy's and St Thomas' NHS Foundation Trust and King's College London. Financial support for KARBAC was provided through the regional agreement on medical training and clinical research (ALF) between Stockholm County Council and Karolinska Institutet, the Swedish Cancer Society, The Gustav V Jubilee foundation and Bert von Kantzows foundation. The KARMA study was supported by Märit and Hans Rausings Initiative Against Breast Cancer. The KBCP was financially supported by the special Government Funding (VTR) of Kuopio University Hospital grants, Cancer Fund of North Savo, the Finnish Cancer Organizations, and by the strategic funding of the University of Eastern Finland. kConFab is supported by a grant from the National Breast Cancer Foundation, and previously by the National Health and Medical Research Council (NHMRC), the Queensland Cancer Fund, the Cancer Councils of New South Wales, Victoria, Tasmania and South Australia, and the Cancer Foundation of Western Australia. Financial support for the AOCS was provided by the United States Army Medical Research and Materiel Command [DAMD17-01-1-0729], Cancer Council Victoria, Queensland Cancer Fund, Cancer Council New South Wales, Cancer Council South Australia, The Cancer Foundation of Western Australia, Cancer Council Tasmania and the National Health and Medical Research Council of Australia (NHMRC; 400413, 400281, 199600). G.C.T. and P.W. are supported by the NHMRC. RB was a Cancer Institute NSW Clinical Research Fellow. The KOHBRA study was supported by a grant from the National R&D Program for Cancer Control, Ministry of Health & Welfare, Republic of Korea (0720450; 1020350; 1420190) and the National R&D Program for Cancer Control through the National Cancer Center (NCC) funded by the Ministry of Health & Welfare, Republic of Korea (HA21C0140). LMBC is supported by the 'Stichting tegen Kanker'. DL is supported by the FWO. The MABCS study is funded by the Research Centre for Genetic Engineering and Biotechnology "Georgi D. Efremov", MASA. The MARIE study was supported by the Deutsche Krebshilfe e.V. [70-2892-BR I, 106332, 108253, 108419, 110826, 110828], the Hamburg Cancer Society, the German Cancer Research Center (DKFZ) and the Federal Ministry of Education and Research (BMBF) Germany [01KH0402]. The MCBCS was supported by the NIH grants R35CA253187, R01CA192393, R01CA116167, R01CA176785 a NIH Specialized Program of Research Excellence (SPORE) in Breast Cancer [P50CA116201], and the Breast Cancer Research Foundation. The Melbourne Collaborative Cohort Study (MCCS) cohort recruitment was funded by VicHealth and Cancer Council Victoria. The MCCS was further augmented by Australian National Health and Medical Research Council grants 209057, 396414 and 1074383 and by infrastructure provided by Cancer Council Victoria. Cases and their vital status were ascertained through the Victorian Cancer Registry. The MEC was supported by NIH grants CA63464, CA54281, CA098758, CA132839 and CA164973. The MISS study was supported by funding from ERC-2011-294576 Advanced grant, Swedish Cancer Society CAN 2018/675, Swedish Research Council, Local hospital funds, Berta Kamprad Foundation FBKS 2021-19, Gunnar Nilsson. The MMHS study was supported by NIH grants CA97396, CA128931, CA116201, CA140286 and CA177150. MSKCC is supported by

grants from the Breast Cancer Research Foundation and Robert and Kate Niehaus Clinical Cancer Genetics Initiative. MyBrCa is funded by research grants from the Wellcome Trust (v203477/Z/16/Z), the Malaysian Ministry of Higher Education (UM.C/HIR/MOHE/06, RP046B-15HTM), Yayasan Sime Darby LPGA Tournament and Cancer Research Malaysia. The NBCS has received funding from the K.G. Jebsen Centre for Breast Cancer Research; the Research Council of Norway grant 193387/V50 (to A-L Børresen-Dale and V.N. Kristensen) and grant 193387/H10 (to A-L Børresen-Dale and V.N. Kristensen), South Eastern Norway Health Authority (grant 39346 to A-L Børresen-Dale) and the Norwegian Cancer Society (to A-L Børresen-Dale and V.N. Kristensen). The NBHS was supported by NIH grant R01CA100374. Biological sample preparation was conducted the Survey and Biospecimen Shared Resource, which is supported by P30 CA68485. The Carolina Breast Cancer Study (NCBCS) was funded by Komen Foundation, the National Cancer Institute (P50 CA058223, U54 CA156733, U01 CA179715), and the North Carolina University Cancer Research Fund. The NGOBCS was supported by the National Cancer Center Research and Development Fund (Japan). The NHS was supported by NIH grants P01 CA87969, UM1 CA186107, and U19 CA148065. The NHS2 was supported by NIH grants UM1 CA176726 and U19 CA148065. The ORIGO study was supported by the Dutch Cancer Society (RUL 1997-1505) and the Biobanking and Biomolecular Resources Research Infrastructure (BBMRI-NL CP16). The PBCS was funded by Intramural Research Funds of the National Cancer Institute, Department of Health and Human Services, USA. Genotyping for PLCO was supported by the Intramural Research Program of the National Institutes of Health, NCI, Division of Cancer Epidemiology and Genetics. The PLCO is supported by the Intramural Research Program of the Division of Cancer Epidemiology and Genetics and supported by contracts from the Division of Cancer Prevention, National Cancer Institute, National Institutes of Health. The SASBAC study was supported by funding from the Agency for Science, Technology and Research of Singapore (A*STAR), the US National Institute of Health (NIH) and the Susan G. Komen Breast Cancer Foundation. The SBCGS was supported primarily by NIH grants R01CA64277, R01CA148667, UMCA182910, and R37CA70867. Biological sample preparation was conducted the Survey and Biospecimen Shared Resource, which is supported by P30 CA68485. The scientific development and funding of this project were, in part, supported by the Genetic Associations and Mechanisms in Oncology (GAME-ON) Network U19 CA148065. The SBCS was supported by Sheffield Experimental Cancer Medicine Centre and Breast Cancer Now Tissue Bank. SEARCH was funded by Cancer Research UK [C490/A10124, C490/A16561] and supported by the UK National Institute for Health Research Biomedical Research Centre at the University of Cambridge. SEBCS was supported by the BRL (Basic Research Laboratory) program through the National Research Foundation of Korea funded by the Ministry of Education, Science and Technology (2012-0000347). SGBCC is funded by the National Research Foundation Singapore, NUS start-up Grant, National University Cancer Institute Singapore (NCIS) Centre Grant, Breast Cancer Prevention Programme, Asian Breast Cancer Research Fund and the NMRC Clinician Scientist Award (SI Category). Population-based controls were from the Multi-Ethnic Cohort (MEC) funded by grants from the Ministry of Health, Singapore, National University of Singapore and National University Health System, Singapore. The Sister Study (SISTER) is supported by the Intramural Research Program of the NIH, National Institute of

Environmental Health Sciences (Z01-ES044005 and Z01-ES049033). The Two Sister Study (2SISTER) was supported by the Intramural Research Program of the NIH, National Institute of Environmental Health Sciences (Z01-ES044005 and Z01-ES102245), and, also by a grant from Susan G. Komen for the Cure, grant FAS0703856. SKKDKFZS is supported by the DKFZ. SMC is part of the Swedish Infrastructure for Medical Population-based Life-course and Environmental Research (SIMPLER), which receives funding from the Swedish Research Council (grants VR 2017-00644, VR 2021-00160). The study is supported by the Swedish Cancer Foundation grant CF 20 0864. The TWBCS is supported by the Taiwan Biobank project of the Institute of Biomedical Sciences, Academia Sinica, Taiwan. UBCS was supported by funding from National Cancer Institute (NCI) grant R01 CA163353 (to N.J. Camp) and the Women's Cancer Center at the Huntsman Cancer Institute (HCI). Data collection for UBCS was supported by the Utah Population Database (UPDB) and Utah Cancer Registry (UCR). The UPDB is supported by HCI, the University of Utah, and NCI grant P30 CA2014. The UCR is additionally funded by the NCI's SEER Program, HHSN261201800016I, and the US Center for Disease Control and Prevention's National Program of Cancer Registries (NU58DP007131). The UCIBCS component of this research was supported by the NIH [CA58860, CA92044] and the Lon V Smith Foundation [LVS39420]. The UKBGS is funded by Breast Cancer Now and the Institute of Cancer Research (ICR), London. ICR acknowledges NHS funding to the NIHR Biomedical Research Centre. The USRT Study was funded by Intramural Research Programof the National Cancer Institute, Department of Health and Human Services, USA. This work was also funded by NCI U19 CA148065-01.

Acknowledgements

We thank all the individuals who took part in these studies and all the researchers, clinicians, technicians and administrative staff who have enabled this work to be carried out. ABCFS thank Maggie Angelakos, Judi Maskiell, Gillian Dite. ABCS thanks the Blood bank Sanquin, The Netherlands. ABCTB Investigators: Christine Clarke, Deborah Marsh, Rodney Scott, Robert Baxter, Desmond Yip, Jane Carpenter, Alison Davis, Nirmala Pathmanathan, Peter Simpson, J. Dinny Graham, Mythily Sachchithananthan. Samples are made available to researchers on a non-exclusive basis. The ACP study wishes to thank the participants in the Thai Breast Cancer study. Special thanks also go to the Thai Ministry of Public Health (MOPH), doctors and nurses who helped with the data collection process. Finally, the study would like to thank Dr Prat Boonyawongviroj, the former Permanent Secretary of MOPH and Dr Pornthep Siriwanarungsan, the former Department Director-General of Disease Control who have supported the study throughout. BBCS thanks Eileen Williams, Elaine Ryder-Mills, Kara Sargus. BCEES thanks Allyson Thomson, Christobel Saunders, Jennifer Girschik, Jane Heyworth and Terry Boyle. The BCINIS study would not have been possible without the major contribution of Ms. H. Rennert, and the contributions of Dr. M. Pinchev, Dr. O. Barnet, Dr. N. Gronich, Dr. K. Landsman, Dr. A. Flugelman, Dr. W. Saliba, Dr. E. Liani, Dr. I. Cohen, Dr. S. Kalet, Dr. V. Friedman of the NICCC in Haifa, and all the contributing family medicine, surgery, pathology and oncology teams in all medical institutes in Northern Israel. The BREOGAN study would not have been possible without the contributions of the following: Manuela Gago-Dominguez, Jose Esteban

Castelao, Angel Carracedo, Victor Muñoz Garzón, Alejandro Novo Domínguez, Maria Elena Martinez, Sara Miranda Ponte, Carmen Redondo Marey, Maite Peña Fernández, Manuel Enguix Castelo, Maria Torres, Manuel Calaza (BREOGAN), José Antúnez, Máximo Fraga and the staff of the Department of Pathology and Biobank of the University Hospital Complex of Santiago-CHUS, Instituto de Investigación Sanitaria de Santiago, IDIS, Xerencia de Xestion Integrada de Santiago-SERGAS; Joaquín González-Carreró and the staff of the Department of Pathology and Biobank of University Hospital Complex of Vigo, Instituto de Investigacion Biomedica Galicia Sur, SERGAS, Vigo, Spain. CGPS thanks staff and participants of the Copenhagen General Population Study. For the excellent technical assistance: Dorthe Uldall Andersen, Maria Birna Arnadottir, Anne Bank, Dorthe Kjeldgård Hansen. The Danish Cancer Biobank is acknowledged for providing infrastructure for the collection of blood samples for the cases. The Danish Breast Cancer Cooperative Group (DBCG) are acknowledged for their provision of clinical case data. CNIO-BCS thanks Guillermo Pita, Charo Alonso, Nuria Álvarez, Pilar Zamora, Primitiva Menendez, the Human Genotyping-CEGEN Unit (CNIO). COLBCCC thanks all patients, the physicians Justo G. Olaya, Mauricio Tawil, Lilian Torregrosa, Elias Quintero, Sebastian Quintero, Claudia Ramírez, José J. Caicedo, and Jose F. Robledo, and the technician Michael Gilbert for their contributions and commitment to this study. Investigators from the CPS-II cohort thank the participants and Study Management Group for their invaluable contributions to this research. They also acknowledge the contribution to this study from central cancer registries supported through the Centers for Disease Control and Prevention National Program of Cancer Registries, as well as cancer registries supported by the National Cancer Institute Surveillance Epidemiology and End Results program. The authors would like to thank the California Teachers Study Steering Committee that is responsible for the formation and maintenance of the Study within which this research was conducted. A full list of California Teachers Study (CTS) team members is available at https://www.calteachersstudy.org/team. DIETCOMPLYF thanks the patients, nurses and clinical staff involved in the study. The DietCompLyf study was funded by the charity Against Breast Cancer (Registered Charity Number 1121258) and the NCRN. We thank the participants and the investigators of EPIC (European Prospective Investigation into Cancer and Nutrition). ESTHER thanks Hartwig Ziegler, Sonja Wolf, Volker Hermann, Christa Stegmaier, Katja Butterbach. FHRISK and PROCAS thank NIHR for funding. The GENICA Network: Dr. Margarete Fischer-Bosch-Institute of Clinical Pharmacology, Stuttgart, and University of Tübingen, Germany [Hiltrud Brauch, RH, Wing-Yee Lo], Department of Internal Medicine, Johanniter GmbH Bonn, Johanniter Krankenhaus, Bonn, Germany [YDK, Christian Baisch], Institute of Pathology, University of Bonn, Germany [Hans-Peter Fischer], Molecular Genetics of Breast Cancer, Deutsches Krebsforschungszentrum (DKFZ), Heidelberg, Germany [UH], Institute for Prevention and Occupational Medicine of the German Social Accident Insurance, Institute of the Ruhr University Bochum (IPA), Bochum, Germany [Thomas Brüning, Beate Pesch, Sylvia Rabstein, Anne Lotz]; and Institute of Occupational Medicine and Maritime Medicine, University Medical Center Hamburg-Eppendorf, Germany [Volker Harth]. HKBCS thanks Hong Kong Sanatorium and Hospital, Dr Ellen Li Charitable Foundation, The Kerry Group Kuok Foundation for support. HMBCS thanks Peter Hillemanns, Hans Christiansen and Johann H. Karstens. HUBCS thanks Darya Prokofyeva and

Shamil Gantsev. ICICLE thanks Kelly Kohut, Michele Caneppele, Maria Troy. KARMA and SASBAC thank the Swedish Medical Research Counsel. KBCP thanks Eija Myöhänen. kConFab/AOCS wish to thank Heather Thorne, Eveline Niedermayr, all the kConFab research nurses and staff, the heads and staff of the Family Cancer Clinics, and the Clinical Follow Up Study (which has received funding from the NHMRC, the National Breast Cancer Foundation, Cancer Australia, and the National Institute of Health (USA)) for their contributions to this resource, and the many families who contribute to kConFab. We thank all investigators of the KOHBRA (Korean Hereditary Breast Cancer) Study. LMBC thanks Gilian Peuteman, Thomas Van Brussel, EvyVanderheyden and Kathleen Corthouts. MABCS thanks Milena Jakimovska (RCGEB "Georgi D. Efremov"), Snezhana Smichkoska, Emilija Lazarova, Marina Iljoska (University Clinic of Radiotherapy and Oncology), Katerina Kubelka-Sabit, Dzengis Jasar, Mitko Karadjozov (Adzibadem-Sistina Hospital), Andrej Arsovski and Liljana Stojanovska (Re-Medika Hospital) for their contributions and commitment to this study. MARIE thanks Petra Seibold, Ursula Eilber and Muhabbet Celik. MBCSG (Milan Breast Cancer Study Group): Paolo Radice, Bernard Peissel, Jacopo Azzollini, Erica Rosina, Daniela Zaffaroni, Bernardo Bonanni, Irene Feroce, Mariarosaria Calvello, Aliana Guerrieri Gonzaga, Monica Marabelli, and the personnel of the Cogentech Cancer Genetic Test Laboratory. The MCCSs was made possible by the contribution of many people, including the original investigators, the teams that recruited the participants and continue working on follow-up, and the many thousands of Melbourne residents who continue to participate in the study. The MISS study group acknowledges the former Principal Investigator, Professor Håkan Olsson. We thank the coordinators, the research staff and especially the MMHS participants for their continued collaboration on research studies in breast cancer. MSKCC thanks Marina Corines, Lauren Jacobs. NBHS and SBCGS thank study participants and research staff for their contributions and commitment to the studies. MyBrCa thanks all study participants and all research staff and clinician at Cancer Research Malaysia, University Malaya, and Sime Darby Medical Centre who assisted in recruitment, interviews, sample handling, and data cleaning for their contributions and commitment to the Malaysian Breast Cancer Genetic Study. For NHS and NHS2 the study protocol was approved by the institutional review boards of the Brigham and Women's Hospital and Harvard T.H. Chan School of Public Health, and those of participating registries as required. We would like to thank the participants and staff of the NHS and NHS2 for their valuable contributions as well as the following state cancer registries for their help: AL, AZ, AR, CA, CO, CT, DE, FL, GA, ID, IL, IN, IA, KY, LA, ME, MD, MA, MI, NE, NH, NJ, NY, NC, ND, OH, OK, OR, PA, RI, SC, TN, TX, VA, WA, WY. The authors assume full responsibility for analyses and interpretation of these data. PREFACE thanks Sonja Oeser and Silke Landrith. The RBCS thanks Jannet Blom, Saskia Pelders, Wendy J.C. Prager – van der Smissen, and the Erasmus MC Family Cancer Clinic. SBCS thanks Sue Higham, Helen Cramp, Dan Connley, Ian Brock, Sabapathy Balasubramanian and Malcolm W.R. Reed. We thank the SEARCH and EPIC teams. SGBCC thanks the participants and all research coordinators for their excellent help with recruitment, data and sample collection. SGBCC Investigators: Benita Kiat-Tee Tan, Veronique Kiak Mien Tan, Geok Hoon Lim, Ern Yu Tan, Su-Ming Tan. SKKDKFZS thanks all study participants, clinicians, family doctors, researchers and technicians for their contributions and commitment to this study. We thank the SUCCESS Study teams in Munich,

Duessldorf, Erlangen and Ulm. UBCS thanks all study participants as well as the ascertainment, laboratory, analytics and informatics teams at Huntsman Cancer Institute and Intermountain Healthcare for their important contributions to this study. UCIBCS thanks Irene Masunaka. UKBGS thanks Breast Cancer Now and the Institute of Cancer Research for support and funding of the Generations Study, and the study participants, study staff, and the doctors, nurses and other health care providers and health information sources who have contributed to the study. We acknowledge NHS funding to the Royal Marsden/ICR NIHR Biomedical Research Centre.
